# The Influence of Relationships Within Behavioral Weight Management Interventions: An Overview

**DOI:** 10.1111/obr.70176

**Published:** 2026-06-17

**Authors:** Claire E. Torrens, Pauline Campbell, Scott Findlay, Julie Cowie, Ronan O'Carroll, Pat Hoddinott, Gozde Ozakinci, Barbara Farquharson

**Affiliations:** ^1^ Centre of Health and Community Research (CHeCR Formerly NMAHP‐Research Unit), Faculty of Health Sciences and Sport University of Stirling Stirling UK; ^2^ Department of Nursing, Community and Public Health Glasgow Caledonian University Glasgow UK; ^3^ Division of Sport, Exercise and Health, School of Health and Life Sciences University of the West of Scotland Blantyre UK; ^4^ Yunus Centre for Social Business and Health Glasgow Caledonian University Glasgow UK; ^5^ Division of Psychology, Faculty of Natural Sciences University of Stirling Stirling UK

**Keywords:** obesity, relationships, retention, weight loss

## Abstract

Behavioral weight management interventions have been shown to be effective for weight loss. However, a common challenge is difficulty in engaging with the public, participants, or service‐users across the research continuum. User–provider relationships are key in weight management and may be integral to continuing engagement. This overview aimed to understand the nature of relationships within behavioral weight management interventions delivered in research studies and healthcare settings, how these may influence retention as well as potential relationship barriers and facilitators. Six databases were searched; 20 reviews (27 reports) were included. Most reviews investigated weight management delivered face‐to‐face by healthcare professionals across different settings. The Theoretical Domains Framework guided the synthesis of qualitative and quantitative data regarding user–provider relationships. GRADE CER‐Qual was used to assess the confidence of findings. Thirty‐eight findings were categorized to domains in the framework in addition to two cross‐cutting themes. High confidence evidence demonstrated that the domains of knowledge, skills, social/professional role or identity, environmental context and resources, and behavioral regulation provided challenges as well as enabling relationships in weight management. The central domain underpinning the influence of relationships on retention was social influences, with weight stigma a pervasive theme recurring across domains. Application of a theoretical lens to consider cognitive, affective, social, and environmental influences helped better understand user–provider relationships as a potential mechanism through which retention‐related behaviors in weight management can be driven or deterred. This overview suggests that improving user–provider relationships should be considered a useful retention strategy in weight management programs.

## Summary

- Weight stigma should be recognized as a potential harm experienced by users as well as a reason for disengagement and should therefore be a central consideration in developing weight management programs and retention strategies, including support and training for providers to develop knowledge and skills.
- User–provider relationships can be a positive draw for patients and participants in obesity research and weight management programs. As a determinant of engagement, these relationships are therefore key to intervention success.
- Consideration should be given to the intersection between the mode of delivery, user–provider relationship, and retention when developing behavioral weight management interventions.

**Abbreviations**
AMSTAR‐2: A MeaSurement Tool to Assess systematic Reviews
BCIO: Behaviour Change Intervention Ontology
BED: Binge Eating Disorder
BMI: Body Mass Index
BWMI: behavioral weight management interventions
CALO‐RE: Coventry, Aberdeen & London—Refined taxonomy
CASP: Critical Appraisal Skills Programme Assessment
CDSR: Cochrane Database of Systematic Reviews
CMS: Centers for Medicare & Medicaid Services guidelines
EMBASE: Excerpta Medica Database
EPOC: Effective Practice and Organisation of Care
EPPI: Evidence for Policy and Practice Information
GP: General Practitioner/Family Practitioner
GRADE CERQual: Grades of Recommendation Development, and Evaluation—Confidence in the Evidence from Reviews of Qualitative research
GROOVE: Graphical Representation of Overlap for OVErviews tool
HbA1c: Hemoglobin A1C
iSoQ: interactive Summary of Qualitative Findings
MeSH: Medical Subject Headings
MI: Motivational Interviewing
PCP: Primary Care Practitioners
PRIOR: preferred reporting items for overviews of reviews
PRISMA: Preferred Reporting Items for Systematic review and Meta‐Analysis
PROSPERO: international prospective register of systematic reviews
SDT: self‐determination theory
STAR‐LITE: standardized reporting of adult behavioral weight management interventions to aid evaluation
SMI: Severe Mental Illness
SMS: Short Message Service
TDF: Theoretical Domains Framework

## 1 Introduction

Globally, as many as one in eight people live with obesity [1] making this condition a major public health issue. Several different types of interventions, including surgery, medication, and behavioral modification of diet and activity [2], have been shown to have varying degrees of success for effective weight loss [3]. However, identifying the specific components responsible for effective intervention, particularly in behavioral weight management, has proved challenging [4]. An essential and often overlooked component is the role of the relationship between those providing the intervention (“provider”) and those receiving the intervention (“user”) [5]. We refer to this as the user–provider relationship hereafter.

Bordin (1979) described the user–provider relationship or “working alliance” as central to the change process and generalisable to all “change” settings [6]. In psychotherapy, user–provider relationships have been shown to be a mediator and predictor of therapy outcomes, associated with engagement irrespective of modality and across diagnostic groups [7, 8, 9]. There is some evidence pointing to the importance of user–provider relationships in retention within behavioral weight management interventions (BWMI) in terms of adherence, attendance, and attrition [10, 11, 12, 13]. Research regarding predictors of retention has tended to rely upon baseline and outcome data routinely collected during trials of BWMI [10, 14] making it an underexplored area. There has been inadequate consideration of “how” user–provider relationships could influence treatment, retention, or engagement [8, 15, 16] including within BWMIs [17].

Failing to retain participants can have a financial cost to trials [18]. In addition, disengagement from weight management programs, such as loss to follow‐up or withdrawal, can diminish the quality and reliability of findings through loss of outcome data [19]. The current evidence base regarding user–provider relationships in obesity research is fragmented, and the factors undermining or facilitating these relationships have not been comprehensively synthesized. Only one systematic review has explored the impact of the relationship on weight loss and retention in primary care settings [17]. Based on findings from 11 studies, this review concluded that the relationship between general practitioners and patients was essential to an intervention's success but noted that the components of the relationship were poorly defined or documented. More recently, a scoping review highlighted two barriers directly related to the user–provider relationship [20]. This review found that clinicians intentionally avoided discussing obesity with clients due to worries about stigmatization and experienced challenges from the overlap between personal and professional relationships in rural areas.

Difficulties with engagement, specifically retention, are not unique to BWMI. Consequently, there has been prioritization of research in this area across a wide range of behavioral interventions [16, 21]. A deeper understanding of the factors which underpin effectiveness [18, 22] and facilitate successful engagement (the reasons for participants opting to remain as opposed to leave a program or study) across the research continuum is required [23]. Improved knowledge of user–provider relationships could aid development of appropriate retention strategies maximizing participation across the full duration of a program or study. This would in turn support high‐quality, value‐for‐money research with the potential to improve weight loss outcomes [24].

Therefore, with awareness of the large number of existing review evidence on this topic this overview, or systematic review of reviews, aimed to synthesize: (a) what is currently known about user–provider relationships within studies of BWMIs for people living with obesity (b) the barriers and facilitators of provider‐user relationships in BWMI in different settings, and (c) the impact of these relationships on retention.

## 2 Materials and Methods

### 2.1 Design

An overview (a systematic review of reviews) was conducted using established methodology [25] and reported in accordance with the preferred reporting items for overviews of reviews (PRIOR) statement [26] (see Table S1). A protocol was published a priori in PROSPERO (CRD42021237358) [27]. Key definitions for terminology used throughout this overview are provided in Box 1. Additional relationship definitions and terminology are provided in Table S3.

Box 1 **Key definitions used throughout this overview.**

**Retention:** An inclusive approach guided by the standardized reporting of lifestyle weight management interventions to aid evaluation (STAR‐LITE) core outcomes set [28] was used to investigate continued commitment to a program and/or research study for example: retention, attendance, attrition, completion, loss to follow‐up, drop‐out, withdrawal, adherence, and engagement.

**Systematic review**: Using the EPPI Centre definition [29], included reviews had to use explicit and transparent methods, follow a standard set of stages, be considered accountable, replicable, and updateable.

**User–provider relationship**: Bordin's definition of the therapeutic or working relationship was used conceptualize and assist interpretation of user–provider relationships within BWMIs. This describes “a collaboration for change” comprising three component parts: i) mutual agreement of goals ii) the tasks of each of the partners within the relationship and iii) the bond (i.e., liking, caring, accepting, trusting) between partners necessary to sustain the change process [6, 30].

### 2.2 Eligibility Criteria

The eligibility criteria are summarized in Table 1. Where reports included multiple reviews, we focused only on those which met the inclusion criteria.

**TABLE 1:** Eligibility criteria.

Inclusion criteria	Exclusion criteria
Systematic review (see Box 1) Adults (≥ 18 years) living with obesity (BMI ≥ 30 kg/m^2^) seeking intentional weight loss. Studies of adults living with overweight and obesity were also included if the majority of participants (> 50%) were living with obesity. Individual behavior change interventions delivered remotely or face‐to‐face by researchers or health practitioners. Focused on weight, people living with comorbidities were included if the focus was weight and weight loss during pregnancy. Described user–provider relationships (e.g., client‐practitioner, participant‐researcher, participant‐trial staff) in any setting e.g., research, primary care Provided an explanation of the intervention including the form or manner of intervention delivery (relationship)	Reviews of surgical or pharmacological interventions Populations with or where the focus was on prevention of weight gain in pregnancy. Group‐based behavior change interventions. Focused exclusively on populations with a physical or mental comorbidity, for example, diabetes or eating disorders, such as BED where another clinical marker (e.g., HbA1c) was the primary outcome or focus, not obesity or weight management. Reported on relationships outside of the study e.g., spousal or family

Abbreviations: BED—binge eating disorder; BMI—body mass index; HbA1c—Hemoglobin A1C.

### 2.3 Search Strategy

A comprehensive search strategy (Supporting Information S2) using MeSH headings and free text terms was developed with an information specialist (CC), peer‐reviewed and piloted to ensure identification of relevant studies. Six electronic databases were searched (searched in January 2021; updated targeted search 22nd November 2025):

- MEDLINE (EBSCO)
- Cochrane Database of Systematic Reviews (CDSR) (https://www.cochranelibrary.com),
- Excerpta Medica Database (Embase) (Ovid)
- PsycINFO (Ovid)
- Evidence for policy and practice (EPPI) centre (http://eppi.ioe.ac.uk/)
- International prospective register of systematic reviews (PROSPERO) (https://www.crd.york.ac.uk/prospero/)

Backward and forward citation searches of included studies were also conducted. Searches were limited using an appropriate study design filter for systematic reviews, and to English only publications published from 1998, reflecting changes following adoption of the Human Rights Act (1998) [31] in the UK and reconfiguration of services across the USA, both of which changed health and social care for those living with long‐term conditions [32].

Supplementary searches for gray literature were also conducted in Google Scholar (https://scholar.google.co.uk), limited to the first 50 articles by relevance. Experts in the field were also contacted through the Game of Stones project [33] management and steering groups to identify any additional relevant reports.

### 2.4 Study Selection

Titles, abstracts and full text studies were screened by two independent reviewers (CT, PC). Inter‐rater agreement was calculated using percentage agreement and Cohen's kappa. A third reviewer (BF) was involved in the case of any disagreement for full text screening as well as coding of mapped reviews to the traffic light system described below.

### 2.5 Sampling Strategy

A sampling strategy, adapted from guidance on sampling for qualitative evidence syntheses, from the Cochrane Effective Practice and Organisation of Care (EPOC) group [34] was utilized. This supported identification of comprehensive reviews, which aimed to achieve variation but reduce the volume of data to be synthesized, thereby mitigating the potential threat to the quality of the synthesis. Preliminary data was mapped from reviews including the review author, year, country and aim; type of review; review setting; participant characteristics, intervention description including type of behavior change intervention(s), relationship description, weight‐related outcomes and retention (e.g., attendance, completion, drop‐out/withdrawal, loss to follow‐up, engagement). Reviews were then discussed by two reviewers (CT, PC) according to the mapped data and coded considering the comprehensiveness of their exploration and description of any user–provider relationship using a traffic light system (see Table S4):

- red (very little description of the user–provider relationship presented in the results/discussion),
- amber (some or a reasonable amount of description of the user–provider relationship presented in the results/discussion) and
- green (incorporating a rich description of relationships and comprising the main synthesis of this overview)

### 2.6 Overlap

**FIGURE 1:**
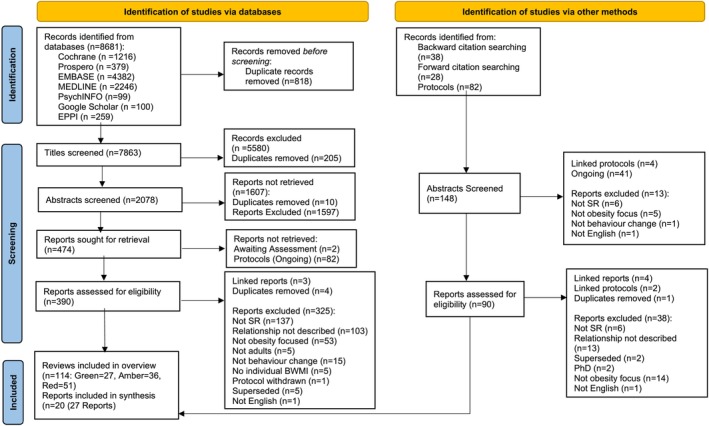
PRISMA flow diagram [36].

Overlap between primary studies in reviews (judged as the proportion of primary studies in one systematic review found in another) was assessed prior to data extraction using the Graphical Representation of Overlap for OVErviews (GROOVE) tool [35]. When multiple papers described the same review or a sub‐set of a review, details were linked or combined. Where there was a more recent update of a systematic review or very high overlap between reviews, older reviews were superseded and excluded (see Figure 1). A visual presentation of overlap of primary studies within systematic reviews can be found in Figure S1.

### 2.7 Data Extraction

Data from the green reviews were extracted by one reviewer (CT) using a pre‐developed data extraction file in excel and cross checked by a second reviewer (PC, JC, SF). Data extraction extended the original mapping with additional information regarding the intervention, described by the review authors, based on an adaption of the STAR‐LITE template [37] including form of delivery, tailoring, how, when and where the intervention was delivered, who delivered it, and behavior change techniques (BCTs) which were coded using the CALO‐RE taxonomy (Table S5) [38]. A more in‐depth description of the user–provider relationship as well as findings in relation to barriers and enablers of the user–provider relationship were also extracted.

### 2.8 Quality Appraisal and Assessment of Confidence

Two reviewers (CT, PC) independently assessed review quality using the AMSTAR 2 (A MeaSurement Tool to Assess systematic Reviews) critical appraisal tool [39] (see Table S6). Criteria from the Critical Appraisal Skills Programme (CASP) qualitative checklist [40] (items: 2, 3, 8, and 9: Is the qualitative methodology appropriate? Was the research design appropriate to address the aims of the research? Is there a clear statement of findings? Was data analysis sufficiently rigorous?) guided quality appraisal for qualitative (e.g., meta‐ethnographies), integrative, or scoping reviews. GRADE CERQual (“Confidence in the Evidence from Reviews of Qualitative research”) [41], supported assessment of confidence in the synthesized findings using the interactive Summary of Qualitative Findings (iSoQ) online tool. Gradings of high, moderate, low or very low were based on: methodological limitations, coherence, adequacy, and relevance.

### 2.9 Synthesis

Synthesis of the quantitative and qualitative evidence was guided by the steps outlined by Hendricks (2021) in their mega‐aggregation of qualitative reviews [42] utilizing the Theoretical Domains Framework (TDF) [43]. The TDF simplifies 33 theories and 128 constructs into 14 domains. Underpinned by psychological theory, providing a theoretical basis for understanding behavior and implementation [43, 44], the TDF has previously been used to consolidate diverse evidence within an overview of self‐care of minor ailments [45]. Extracted relationship data were coded according to the TDF domains and where appropriate were categorized as relationship barriers and/or facilitators (see Table S7). This narrative overview reports on the nature of relationships within BWMIs delivered within intervention studies, including trials, as well as weight management experienced in healthcare settings. We also explore user–provider relationships and their influence on retention and the barriers and facilitators of these relationships.

## 3 Results

### 3.1 Included Reviews

Systematic searches identified 8829 records (original searches: 7292 records; update searches: 1537 records), of which 114 reviews met the eligibility criteria (83% agreement between reviewers; *κ* = 0.526, *p* < 0.001) (see Figure 1). Overall interpretation of overlap according to the GROOVE tool was “slight overlap”; one review [46] was superseded due to high overlap, with no “very high overlap” between reviews (see Figure S1). Following the application of the sampling criteria to 114 reviews, 20 reviews (27 publications) were judged as “green”, 36 reviews were judged as “amber”, and 51 reviews were judged as “red”. Reviews judged as amber or red, with limited description of the relationship, were tabulated and presented in evidence tables (including population, intervention, relationship and retention descriptions) (Table S9).

Of the 20 reviews, three were systematic reviews with meta‐analyses [17, 47, 48], three were quantitative reviews [49, 50, 51], six were qualitative syntheses [44, 52, 53, 54, 55, 56], three were integrative reviews [57, 58, 59], and five were mixed methods reviews [3, 60, 61, 62, 63].

Eleven reviews [3, 17, 47, 48, 50, 51, 56, 59, 60, 61, 63] synthesized evidence from trials of BWMIs including e‐health and telehealth programs incorporating tailoring [61], motivational interviewing (MI) [50] or coaching [59] in addition to weight‐related communication training for primary care doctors [51]. One review [49] investigated observed behavior of primary care providers during weight management consultations. Another review [56] specifically investigated engagement with remotely delivered weight management interventions. Twelve reviews explored user [52, 60, 63], provider [44, 54, 55, 62], or user and provider [3, 53, 57, 58, 60] perspectives regarding weight management programs or delivery, practices, and services in relation to weight management in real‐world settings including experiences of consultations in primary and secondary care. In some cases, the qualitative syntheses were part of a wider mixed methods report which included trials [3, 60], the qualitative data therefore linked to these trials. Further review characteristics are shown in Table 2.

**TABLE 2:** Characteristics of included green reviews.

Author(s), year (setting)	Type of synthesis/(no. studies)	Population/participant(s) description (*n*)	Description of BWMI/identified BCTs identified*	Mode of delivery	Relationship described	Perspective and/or outcome	Retention
Ananthakumar et al. 2020 [52] (Primary Care)	Qualitative. (*n* = 21)	People living with overweight or obesity (*n* = 466)	Brief Intervention: Weight‐related consultation. BCTs: 1,6, 22,32	Face‐to‐face	Doctor–patient	User (thoughts/feelings about consultations and how weight was raised)	Attendance
Avenell et al. 2018 [3, 64] (Any setting)	Mixed methods. (Review 1 RCTs = 131 Review 2 UK interventions = 26 Review 3 Qualitative & mixed methods = 33 Review 4: Economic evaluation = 46)	Adults living with severe obesity. Providers. Review 1: NR Review 2: (*n* = 22,093) Review 3: Participants (*n* = 144). Providers (*n* = 153) Review 4: NA	Lifestyle WMPs. BCTs: 1,5,6,7,8,9,16,22,24,26,29, 33,37	Face‐to‐face Remote: e‐health components	HCP–patient	User and Providers (involved in their care) (views of weight management programs). Weight Change.	Drop‐out, engagement
Dieterich & Demirci 2020 [57] (Setting: NR)	Integrative. (*n* = 14)	Pregnant women living with overweight and obesity (*n* = 1526). HCPs (*n* = 658) (Range: Qual:11–62; Quant:73–1145)	Brief intervention—weight‐related consultation. BCTs: 37	Face‐to‐face	HCP–pregnant women	HCPs (experiences and practices with patients with overweight & obesity)	Response rates to surveys, completion
Henderson 2015 [53] (Primary Care)	Qualitative. (*n* = 9)	Adults with obesity receiving treatment and lay people (*n* = 105). PCPs (*n* = 144)	Obesity‐related primary care services. BCTs: 1,37	Face‐to‐face	PCP–patient	Practitioner and user (perspectives on PC obesity services)	Continuing engagement
Heslehurst et al. 2014 [44] (Setting: NR)	Mixed methods. (*n* = 25)	Pregnant women living with overweight or obesity. HCPs caring for pregnant women (*n* = 3184–1 sample NR)	Brief intervention—weight‐related consultations. BCTs: 6,16,32	Face‐to‐face	HCP–pregnant women	HCPs (perspectives on weight management in pregnancy)	Continuing engagement
Jeffers 2024 [55] (Primary Care)	Qualitative (*n* = 15)	People living with overweight or obesity. HCPs (*n* = 299)	Weight management; eeight‐related consultation BCTs: NS	Face‐to‐face	HCP–patient	Providers' perceptions, and experiences of obesity and its management.	Continuing engagement
Lee 2021 [63] (Community, outpatients)	Mixed methods: Qualitative (*n* = 20) RCTs (*n* = 34)	Adults ≥ 18 years old with SMI living with overweight (BMI 25–29.9 kg/m^2^) or obesity (> 30 kg/m^2^) (Qualitative *n* = 515; RCTs *n* = 4305)	BWMI BCTs: 5,8,12,21,37	Face‐to‐face; online, Hybrid—face‐to‐face and telephone	Researcher–Participant MHCP/HCP–patient	Participant perspectives and experiences; Mean weight change (kg), BMI (kg/m^2^), or % weight change (kg).	Continuing Engagement
McHale et al. 2016 [49] (Primary Care)	Quantitative. (*n* = 20)	Primary care patients living with overweight or obesity. PCPs (*n* = 5798)	Brief interventions—weight‐related consultations. BCTs: 6,8,37	Face‐to‐face	PCP–patient	Weight‐related communication outcomes (e.g., PCP communication behavior e.g., use of MI). Patient weight‐related outcomes (e.g., weight loss, confidence to lose weight, satisfaction)	Continuing engagement
Mold & Forbes 2013 [58] (Community, education secondary care/acute care settings, academic/medical conferences)	Integrative. (*n* = 30)	Patients living with overweight or obesity. HCPs. (*n* = NR)	Weight‐related healthcare interactions BCTs: NS	Face‐to‐face	HCP–patient	Patient or practitioner (experiences or perspectives on obesity (delivery and/or uptake of healthcare services)	Continuing engagement, completion
Obino 2016 [59] (Setting: NR)	Integrative. (*n* = 13)	Adults accessing health coaching (*n* = 6836)	Health coaching for weight loss. BCTs: 5,7,8,16,19,37	Face‐to‐face Remote: Internet & telephone	Health coach–participant	Weight loss	Completion
Patel et al. 2019 [50] (Setting: NR)	Quantitative. (*n* = 16;15 of which were RCTs)	Adults living with overweight or obesity (Mean = 260; Range: 45–1755)	MI for weight loss. BCTs: 37	Face‐to‐face Remote: Internet (email) or Telephone	Lifestyle Coach (including HCPs)‐ Patient	Weight loss outcomes (in kg, lb., or % body weight lost) at baseline & at least 1 follow‐up visit	Retention, continuing engagement
Reading et al. 2020 [51] (Primary, community)	Quantitative. (*n* = 7)	Physicians providing care to adult patients (*n* = 200, range = 14–86). Patients (*n* = 1124, range = 64–574)	Brief Interventions—Weight‐related Consultations. BCTs: 5,37,39	Face‐to‐face Internet: online components	Trainer–physician, Physician–patient	Physician outcomes: uptake of communication skills, knowledge, confidence, self‐efficacy. Patient outcomes: Proximal: motivation, satisfaction. Distal: weight, BP.	Retention
Ryan et al. 2019 [61] (Setting: NR)	Mixed Methods. (*n* = 8)	Participants with overweight or obesity (*n* = 4356)	Computer or human tailored e‐health interventions for weight loss. BCTs: 4,5,6,7,8,12,16,19,25	Remote: e‐health	Remote or human health coach–participant	Weight loss baseline and follow‐up	Continuing wngagement
Samdal et al. 2017 [47] (Any setting)	Meta‐analysis/meta‐regression. (*n* = 48)	Adults with overweight or obesity at risk of developing noncommunicable diseases (*n* = 11,183). Study staff/researchers, healthcare professionals	Interventions to promote change in diet &/or PA. BCTs: 1,5,8,9,10,16,19,22,24,29,37	Face‐to‐face Remote: Internet and telephone	Researcher–participant, HCP–patient	Objective or subjective behavioral measures of PA and/or diet at baseline, short‐term follow‐up (≤ 6 months) and long‐term follow‐up (≥ 12 months) when available.	Attrition, retention
Sturgiss et al. 2021 [17] (Primary Care)	Meta‐analysis. (*n* = 11)	Adults living with obesity (*n* = 3955). PCPs.	Lifestyle interventions (to support improved nutrition, increased physical activity & psychosocial care). BCTs: 5	Face‐to‐face Remote: Internet	PCPs and Patients	Reduction in body weight kg or BMI (%)	Loss to follow‐up, withdrawal
Sutcliffe et al. 2016 [60, 65, 66, 67, 68, 69, 70] (Health sector, community, commercial)	Mixed methods. (*n* = 26 reported over 31 papers). Service‐user studies (*n* = 21), provider studies (*n* = 10), service evaluation (*n* = 15)	Service Users (*n* = 507). Providers (*n* = 234)	Tier 2 WMPs. BCTs: 5,6,8,17,29,35	Face‐to‐face Remote: telephone and Internet (2 studies)	HCP–patient Commercial provider–patient	User (views, perceptions or beliefs about WMPs)	Attendance, continuing engagement
Teixeira et al. 2012 [62] (Primary care, hospitals)	Mixed methods. (*n* = 13)	General public & people living with obesity (*n* = 910). GP/FP (*n* = 2477)	Obesity management by healthcare providers for weight loss. BCTs: 1	Face‐to‐face	General practitioner/family practitioner–patient	Beliefs, attitudes, and practices of GPs/FPs	Response rate, continuing engagement
Wadden et al. 2014 [48] (Primary Care)	Meta‐analysis. (*n* = 12)	Patients living with overweight or obesity (*n* = 3893)	Behavioral counseling/lifestyle intervention, consisting of the following 3 components: diet, physical activity and behavioral. BCTs: 5,7,8	Face‐to‐face Remote: Internet, telephone	Primary care practitioner/trained interventionist–patient	Weight change (kg, BMI, %)	Attrition (% participants who did not contribute an in‐person weight at the end of the study).
Warr et al. 2021 [54] (Primary care)	Qualitative. (*n* = 29)	Adults living with overweight or obesity. GPs, nurses and GP trainees (*n* > 601)	Brief intervention—weight‐related consultations. BCTs: 1,6,16,40	Face‐to‐face	GP/nurse–patient	GPs or nurses (perspectives on discussing weight with patients)	Adherence
Whitehall 2025 [56] (Remote)	Qualitative (*n* = 39)	Adults > 18 years old living with excess weight (adjusted BMI obesity category thresholds inclusive of all ethnic groups (BMI ≥ 27.5 kg/m^2^). Providers of care to adults living with excess weight	Synchronous (real time) interactive remote health Interventions targeting weight loss BCTs: 12,16,19,28	Remote (e.g., telephone, video‐conference, online chat/web forums, interactive text messaging); Hybrid—remote face‐to‐face and remote	HCP–patient; researcher–participant; lifestyle/health coach–participant	Barriers and facilitators to engage with a remotely delivered weight management intervention	Continuing engagement, retention/adherence (duration of usage), automated recorded data including logins data entry, attendance (interaction)

Abbreviations: BCTs—behavior change techniques (*a full list with definitions is provided in Table S5); BMI—body mass index; BP—blood pressure; FP—family practitioner; GP—general practitioner; HCP—healthcare professional; kg—kilograms; MHCP—mental healthcare professional; MI—motivational interviewing; NS—not specified; PA—physical activity; PC—primary care; PCP—primary care practitioner; SMI—severe mental illness; WMP—weight management program.

Weight management was delivered across a wide range of primary care [17, 48, 49, 52, 53, 54, 55, 62], and other (e.g., mixed secondary, community and commercial) settings [3, 44, 47, 51, 58, 60, 63]. Detailed information about the form of delivery (program or consultation duration, number of sessions, length of contact, sessions, or scheduling) were inconsistently reported at review level. Only one review in primary care [48] investigated the intensity of behavioral weight loss counseling aligned to the Centers for Medicare & Medicaid Services (CMS) guidelines (i.e., approximately 14 face‐to‐face, 10‐ to 15‐min sessions over 6 months and a maximum of 22 sessions over 12 months). Two reviews in mixed settings [47, 60] included studies with a longer duration of ≥ 12 weeks [47] describing higher intensity interventions (e.g., 48 sessions, fortnightly over 12 months [60]. User and/or provider perspectives of weight management predominantly described brief interventions, mainly consultations within primary or secondary care with a healthcare provider such as a GP or nurse.

### 3.2 The Nature of Relationships in Weight Management

Most reviews investigated weight management delivered face‐to‐face; five reviews included studies incorporating e‐health and/or tele‐health components [3, 17, 47, 48, 60]. Reviews of interventions delivered remotely [50, 56, 59, 61], using for example text messaging, video‐conference, online chat, the web and/or telephone all incorporated some form of contact with a provider with relationships between health coaches or health counselors and patients. User–provider relationships described in the primary care setting [17, 48, 49, 53, 55, 62] were predominantly between patients and Primary Care Practitioners (PCPs), including GPs, and nurses. One review also described relationships between trained interventionists (e.g., lifestyle coaches, medical assistants, exercise specialists, or dietitians) and patients [48]. In other settings, including secondary care [3, 44, 47, 51, 57, 58, 60, 63] user–provider relationships were again predominantly between healthcare professionals (e.g., GPs, Nurses, Midwives) and patients although Sutcliffe (2015) [60] included community and commercial providers. Avenell (2016) [3] was unique in investigating users with experience of severe obesity (BMI ≥ 35 kg/m^2^) and Samdal (2017) [47] and Lee (2022) [63] both referred to participant‐study staff/researcher relationships in addition to patient‐healthcare professional relationships.

Retention was described in multiple ways across reviews including: completion [50], drop‐out [3, 17, 47, 48], attendance [51, 56, 60], continuing engagement [44, 49, 50, 53, 55, 56, 58, 61, 62, 63], and with reference to adherence to weight management guidance [44, 54, 56, 57]. Only one meta‐analysis of BWMIs in primary care [17] was designed explicitly to investigate the influence of the therapeutic alliance on retention and consider the relationship quality. However, due to the low numbers of included studies and high heterogeneity, the authors were unable to determine the influence of the therapeutic alliance on withdrawal or loss to follow‐up. There was limited focus across reviews upon individual factors, such as equity and gender, known to be important in obesity research [11, 14, 71] and relevant to relationships and retention. Therefore, this was not further explored.

### 3.3 Confidence in Findings

This overview describes 40 findings synthesized from across the included reviews and categorized in accordance with the TDF. Findings fall into three categories related to:

- relationship barriers and facilitators (*n* = 30 findings)
- the influence of relationships on retention (*n* = 8 findings)
- cross‐cutting findings across a number of TDF domains relevant relationships, retention, and mode of delivery (*n* = 2 findings).

**FIGURE 2:**
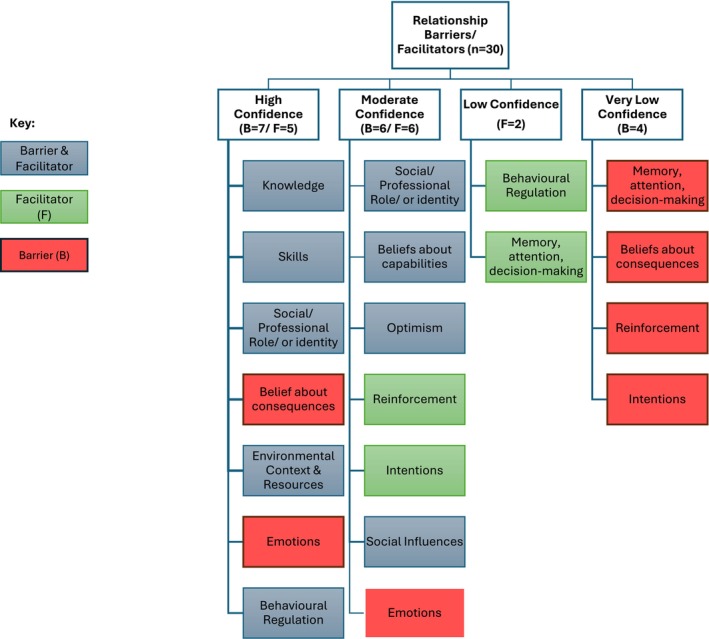
Confidence in overview evidence regarding relationship barriers and facilitators categorized by the TDF.

**FIGURE 3:**
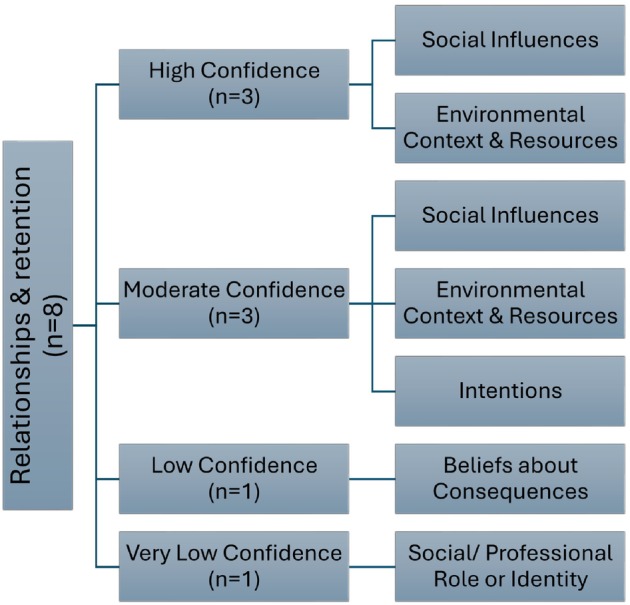
Confidence in overview evidence regarding relationships and retention categorized by the TDF.

A summary of overview findings assessed by GRADE CERQual are provided in Figures 2 and 3. High confidence findings are reported below. Moderate, low, and very low confidence findings are reported in Table S9.

### 3.4 Category 1: Relationship Barriers and Facilitators

In total, 30 findings were related to relationship barriers and/or facilitators (see Figure 2); of these, 12 were considered high confidence findings across seven TDF domains [43] and are described below. All findings aligned to the relationship components of “task” and “bond” [6] with the TDF domains of knowledge, skills, and behavioral regulation also aligning with “goals”.

#### 3.4.1 Knowledge (Barrier and Facilitator)

Providers tended to understand obesity in terms of personal responsibility and negative stereotypes (e.g., lack of will‐power) [44]. They commonly described insufficient knowledge of the wider determinants of obesity, available weight‐related interventions, recommendations or guidelines. A general perception existed that there were few credible resources and inconsistencies in guidance. Providers placed more importance on clinical experience, to guide intervention and information provision rather than guidelines or education. Users indicated that providers made presumptions that all presenting symptoms were weight‐related resulting in users feeling dismissed, anxious or believing they were being punished due to their weight and denied treatments [52]. Gaps in knowledge were related to provider ambivalence, inconsistent practices and reluctance or avoidance of initiating weight discussions and delivering interventions [44, 52, 53, 54, 55, 57, 58, 62].

Providers recognized the need for training to improve knowledge of obesity and its complexity in terms of the broader socioeconomic and psychological determinants. Users wanted a knowledgeable provider who understood obesity and its related comorbidities, such as severe mental health. There was a belief among providers that increased knowledge could enhance outcomes and behavior change, encouraging providers to take responsibility, therefore enabling intervention. Reading (2020) [51] demonstrated that knowledge was improved by training in weight‐related communication. Teixeria (2012) [62] showed that providers with greater knowledge of obesity and experience had less negative attitudes and less difficulty approaching weight management [44, 51, 53, 54, 55, 57, 58, 62, 63].

#### 3.4.2 Skills (Barrier and Facilitator)

Providers described a lack of obesity‐specific and behavior change training. They also discussed not having the necessary interpersonal skills required for weight management. They indicated they were underprepared to provide an empathic and sensitive approach with the appropriate language or tone. They had a perception that their awkwardness could damage rapport. For users, the dearth of these skills among providers had the potential to create negativity, uneasiness, and undermine their motivation to lose weight. Providers, in the absence of formal training, employed various strategies to navigate the perceived challenges of weight‐related interventions, including avoidance (not discussing weight at all), downplaying (minimizing risk), or reframing obesity (linking weight to other health conditions) [3, 49, 51, 52, 53, 54, 55, 57, 58].

Providers recognized the need for obesity‐specific training and improved interpersonal skills in order to provide good quality care and there was motivation and willingness among providers to improve their skills. Training was considered beneficial, improving communication skills, sensitivity, and confidence. Skills training in utilizing the 5A's framework (i.e., Assess, Advise, Agree, Assist, Arrange) or motivational interviewing appeared to enhance physician outcomes. Users wanted providers to be empathic, to listen and respond appropriately. Providers believed using person‐centred and motivational interviewing approaches and good communication skills, incorporating respect, empathy, and nonjudgmental attitudes, improved sensitivity and facilitated behavior change and weight‐related outcomes [44, 47, 48, 49, 51, 52, 53, 54, 55, 57, 58, 60, 63].

#### 3.4.3 Social/Professional Role and Identity (Barrier and Facilitator)

A general lack of consensus and clarity on who takes responsibility for weight management was described by both providers and users. Providers were conflicted by recognizing they were expected to take a role in weight management, by the healthcare system and by users, but also perceiving weight management as an individual's responsibility. Users had often received the message that their weight was their responsibility. Doctors commonly considered weight management was out‐with the scope of their role or they were peripheral, considering others, such as nurses, better placed. Even providers who accepted a level of responsibility did so reluctantly. For some providers (i.e., GPs) “intervening was not worth their time” [54] unless there were comorbidities or high risk. *“Professional ambivalence”* was described from both provider and user perspectives in terms of a disempowered provider with negative, ambiguous and ambivalent beliefs about users and their care resulting in ambivalent users [3, 53, 54, 55, 57, 58, 62].

The qualities and style of providers were important for relationship building. Maintaining a relationship with a professional and gaining encouragement and support from a provider with expertise in weight loss were viewed by users as important. Users valued providers they could trust, and that were open, caring, and nonjudgmental. They expected providers to treat them with dignity and respect. The structure of having existing or ongoing relationships with providers built a strong foundation, motivating users to act and making interactions less difficult [3, 17, 52, 54, 56, 58, 60].

#### 3.4.4 Beliefs About Consequences (Barrier)

Provider's held negative beliefs about the effectiveness of guidelines and weight loss interventions. They described fears regarding initiating and intervening in weight loss, expecting users to react badly, become anxious or defensive, believe they were being blamed or stigmatized, and that intervention would negatively impact their relationships. These negative perceptions were associated with difficulties approaching weight loss and the use of avoidance strategies, such as downplaying the risk or seriousness of obesity when interacting with users [3, 44, 53, 54, 55, 57, 62].

#### 3.4.5 Environmental Context and Resources (Barrier and Facilitator)

Weight management programs which are unable to provide intensive support, in particular in the initial stages, have been shown to be less effective for weight loss [60]. Both providers and users referred to the insufficient time available to address obesity within clinical practice. Limited resources and inappropriate equipment including furniture and scales created uncomfortable, stigmatizing and discriminating environments, presenting barriers to healthcare for people living with obesity and provision of best care by providers. A dearth of resources, in terms of standardized procedures and experienced providers, meant practitioners with limited skills and capabilities often provided weight management or felt constrained in what they could offer [3, 48, 52, 53, 54, 55, 57, 58, 62].

Both providers and users understood the need for a supportive healthcare system that recognizes the complexity of obesity and prioritizes weight management. Opportunities for weight management and consistency in practice were facilitated when appropriate guidance, formalized frameworks, local resources, and care pathways were available within the system. Users valued tools and tailored content, such as handbooks and pedometers, that could support weight loss tasks. In supportive environments providers felt legitimized and empowered in their role. This enhanced consistency in practice while mitigating negative consequences of weight management. Strengthening the capacity for high‐intensity interventions where regular contact is possible, duration longer and/or flexible with the possibility of a gradual reduction in support was considered key to enable bonds and supportive relationships to develop [3, 17, 44, 53, 54, 55, 60, 61, 63].

#### 3.4.6 Emotions (Barrier)

Providers commonly felt awkward, uncomfortable, embarrassed, frustrated, anxious, and powerless in approaching weight management including initiating weight loss discussions. These feelings contributed to the negative attitudes, described by both users and providers, and influenced whether weight‐related conversations occurred and whether appropriate information or advice was provided. Often these negative feelings were related to inadequate resourcing within work settings, their capabilities, and concerns about the negative consequences of weight loss interactions such as stigmatizing users. Providers' negative feelings were considered to affect patient satisfaction with services and had the potential to counter potentially positive interactions achieved through the use of approaches such as Motivational Interviewing [44, 53, 54, 57, 62].

#### 3.4.7 Behavioral Regulation (Barrier and Facilitator)

Weight management is not consistently prioritized by providers, with other activities taking precedence when they are time‐poor. Users consistently reported having only occasional interactions about obesity. Users and providers' views were often misaligned regarding important features of weight management programs. Users' perspectives indicated that psycho‐social components such as changing attitudes and developing supportive relationships were more critical features, whereas dietary advice, physical activity and goal‐setting were of less value. For providers, more standard intervention components were considered important, including weight assessment, nutritional advice, and physical activity, and as a result, were more routinely addressed [3, 44, 49, 52, 54, 60, 62].

The combination of “what” was done and “how” it was done determined the success of BWMIs and how they were valued by users and providers. There was importance in delivering behavior change with professional respect and empathy [47]. Developing self‐regulation skills in users was driven by provider directiveness, feedback, communication style, and supportive relationships. These components of the user–provider relationship were the “scaffolding” for self‐regulation and behavior change, reducing reliance on providers. Both providers and users appreciated having a level of flexibility within interventions. Users in particular valued flexibility in relation to timing, the degree of support, as well as what was offered in terms of diet and exercise [3, 47, 56, 59, 60].

### 3.5 Category 2: The Influence of Relationships on Retention

Eight synthesized findings were specific to the influence of relationships on retention (see Figure 3). Of these, three were high confidence findings, coded to the “task” and “bond” relationship components and are described below.

#### 3.5.1 Social Influences

A common finding from provider and user perspectives was how the experience of negative, ambivalent and stigmatizing interactions with providers (e.g., primary care practitioners such as GPs and Nurses) led to feelings of disempowerment of users and were linked to disengagement with interventions and/or obesity services [44, 53, 54, 58, 63].

Both provider and users' frequently reported that positive, unambiguous user–provider interactions with clear roles, that were friendly, nonjudgmental and encouraging, facilitated the development of bonds and trust, empowering users and acted as “positive pulls” for continued engagement with an intervention [3, 53, 58, 60].

#### 3.5.2 Environmental Context and Resources

The setting, context, and intensity of BWMIs were considered important for continued engagement. Some reviews reported that face‐to‐face interactions with longer duration and higher intensity, with support to attend were more effective for continued engagement. However, for remote interventions increasing access to technology with the need for less travel and reduced cost commonly facilitated continued engagement. There was some indication that providing the opportunity for human contact, through for example interactivity in remote interventions, enhanced the connection, and improved accountability in contrast to support that was provided by only remote methods. User and provider perspectives somewhat differed in what was valued. For providers it was familiarity of environments which were perceived as a draw for women during pregnancy. Whereas users valued novel interventions which were decidedly different from interventions they had tried previously, had relevance, or matched their values or needs [3, 44, 50, 56, 60, 63, 72].

### 3.6 Category 3: Relationships, Retention and Mode of Delivery

Two additional findings were also identified and related to the intersectionality between user–provider relationships, retention (engagement or disengagement), and mode of delivery (see Table S10). The high confidence finding, reported here, rather than coding specifically to one TDF domain, was linked to several TDF domains, namely: skills, knowledge, social influences, belief in capabilities, environmental context and resources, and emotions. Evidence relevant to this finding is also discussed within TDF domains found in the table of lower confidence findings (see Table S9). Two reviews [56, 63] clearly described mode of delivery, although this was also discussed across a number of reviews with interventions delivered face‐to‐face, remotely, and by hybrid methods. The use of technology within remote interventions had both benefits and challenges. Whereas offering the ability to reduce threat for users when compared to face‐to‐face interventions and lessening costs by removing the need to travel, technology was also found to be impractical and difficult for many users. There was dissatisfaction with technology used in interventions and a perceived lack of skills and knowledge, which caused anxiety and embarrassment, a fear of failure, thus leading to disengagement. Engagement with interventions tended to be supported by previous experience, knowledge, and confidence. Human contact, with a level of intensity and face‐to‐face interactions, including when remote delivery was the core mode of delivery, was shown to enhance engagement and was often preferred by users [56, 63].

## 4 Discussion

This overview provides important new insights regarding user–provider relationships from both qualitative and quantitative reviews across different settings. The synthesized findings have produced high confidence evidence, with 12 findings related to the barriers and facilitators of relationships within BWMI and the crucial influence of relationship quality on the success of BWMI in research and healthcare settings, in addition to three high confidence findings regarding the influence of relationships on retention. The parallels between a program‐based and research context in retention‐related behavior (e.g., attendance at assessments, completion of sessions, continuing engagement with/drop‐out from program sessions or trial appointments/assessments) mean that these findings will have importance for trialists, researchers, providers and users.

User–provider relationships have been shown to positively influence outcomes in psychotherapeutic settings [8, 9]. Although underexplored, there is evidence of the importance of relationships in the management of long‐term conditions [73, 74, 75, 76]. Certainly, user–provider relationships are commonly regarded as at the heart of person‐centred care [77] and a key competency within practice standards [77, 78, 79, 80] for healthcare professionals working with a range of healthcare needs. In this overview, user–provider relationships were shown to influence weight management and retention outcomes. There were, however, several inter‐related factors identified in BWMI that challenged and/or facilitated effective relationships.

An under‐resourced, underprepared workforce with insufficient time and a lack of clarity regarding roles, from the perspective of users and providers, could create stigmatizing environments in which user–provider interactions took place. Weight stigma was a pervasive and recurring theme throughout. Users described the stigma they experienced, and providers often reported negative attitudes and beliefs about users. Providers reported an inability to navigate the complexity of weight loss and the associated stigma, resulting in ambivalence, placing users at risk of receiving substandard care.

However, unambiguous user–provider relationships, with clear roles, were facilitated by providers having adequate knowledge and skills supported by appropriate guidance, training, and resources, and the ability to prioritize weight management. The qualities and communication style of providers supported the user–provider bond, reduced stigmatizing environments and encounters, leading to continued engagement.

This overview reflects findings from a previous scoping review of barriers and facilitators of effective weight management [20], where relationships and stigma were reported as barriers. However, the findings here demonstrate that it is the interplay between, the task, goals, and bonds within relationships and key factors, determined by the TDF, such as social influences, environmental context and resources, social/professional role and identity, rather than individual factors alone that are central to successful weight management.

Recent changes in delivery of BWMI from face‐to‐face towards digitalized modes of delivery are reflected in this overview with both challenges and benefits outlined [50, 56, 61]. E‐health interventions have been shown to have poorer engagement, with drop‐out after a short period of time a common challenge [81]. Nevertheless, this overview found evidence that e‐health interventions can offer benefits such as improved access, reducing some of the common engagement difficulties such as travel. There were, however, practical problems with e‐health interventions as well as negative perceptions regarding ease of use and technological fears also reported.

The importance of social influences upon relationships and engagement was demonstrated through the evidence that human contact within remote interventions promoted engagement. This finding is reflected in another recent overview of the effectiveness of e‐health interventions which concluded that those interventions with human contact work better than those that are fully automated [82]. Due consideration should be taken when developing and delivering BWMI to consider the intersection between individual characteristics, mode of delivery, and engagement with potential tailoring to address specific needs.

### 4.1 Strengths and Limitations

This overview used rigorous methodology including use of a “best fit” framework [83] to synthesize of a wide range of qualitative and quantitative review evidence. Taking a broad approach this synthesis also captured user and provider perspectives, to understand relationship barriers and facilitators as well as considering research and real‐world factors influencing a person's decision to remain engaged in a weight‐related program or study.

Similar to previous research, we found limitations within reporting and heterogeneity in the research evidence. There was little explicit conceptualization of relationships, inconsistent measurement and definition of retention, and data relevant to inequalities could not be extracted. This made direct comparisons of relationships and retention unfeasible, and prevented a planned analysis of important characteristics in BWMI such as gender and socio‐economic status [10, 11, 14, 17, 84]. This restricted the ability to understand different populations in terms of relationships and retention and also, importantly, how they might be served better.

### 4.2 Practice Recommendations

- Weight stigma is inherent within weight loss interactions and can result in damaging experiences for users. More work is required to increase provision of effective, nonstigmatizing weight management within healthcare. The implementation of the proposed update of the UK obesity guidelines [85] with greater emphasis on stigma may go some way to support this.
- The importance of relational factors in delivering behavioral interventions [5, 86] should be recognized as a priority in the design and development of future weight management programs.

### 4.3 Research Recommendations

- This overview was underpinned by the broad theoretical domains of the TDF rather than a specific behavior change theory. However, concepts such as autonomy, competence, and relatedness were interwoven throughout provider and user narratives. According to Self‐Determination Theory (SDT) [87] these are central to creating environments which facilitate engagement. Further research regarding relationships in BWMI may benefit from more theory‐informed interventions, underpinned, for example, by SDT.
- Clear, consistent and comprehensive conceptualization and reporting of retention, relationships, adverse events, and important characteristics such as gender and socio‐economic status are required at study and review level in obesity research. More clarity could avoid under‐reporting harms, such as weight‐related stigma, often common in trials [88] and enable further understanding of the influence of relationships in BWMI.
- Improved conceptualization of relationships could be supported through greater utilization of taxonomies and ontologies such as the recently developed Behaviour Change Intervention Ontology (BCIO) across different types of reviews [89].

## 5 Conclusions

The breadth and depth of the review evidence suggests that in weight management interventions, positive user–provider relationships are essential to maximize retention. The TDF enabled a theoretical lens to be applied to consider cognitive, affective, social, and environmental influences to better understand the potential mechanisms which drive or deter retention‐related behaviors in weight management. Future research and implementation of weight management interventions should consider social influences such as weight stigma as an important factor in user–provider relationships and that these relationships are a critical determinant of retention.

## Author Contributions

**Concept and design:** Torrens, Campbell, and Farquharson, O'Carroll. **Searches and Selection:** Torrens, Campbell. **Extraction:** Torrens, Campbell, and Findlay, Cowie. **Risk of Bias:** Torrens, Campbell. **Synthesis:** Torrens, Campbell. **Drafting of the manuscript:** Torrens, Campbell, Farquharson, O'Carroll, Hoddinott, Ozakinci, and Findlay, Cowie.

## Conflicts of Interest

Authors C.E.T. and P.H. were funded by the NIHR (ISRCTN91974895) as Research Fellow and Chief Investigator respectively to deliver the Game of Stones trial, a behavioral text message intervention with endowment financial incentives. C.E.T. is an executive committee member of the UKSBM and this overview forms part of a PhD undertaken by C.E.T. P.T. received CSO research grants for weight management trials and is a panel member of the National Institute for Health Research, School of Primary Care Funding Panel. J.C. and P.C. have received a CSO research grant for a healthy weight and wellbeing intervention with financial incentive. P.C. has also received CSO and NIHR research grants unrelated to obesity research. R.C. has received research grants unrelated to obesity research. B.F. has received research grants from BHF and the Burdett Trust for Nursing unrelated to obesity research. G.O. is President Elect of the European Health Psychology Society. No conflicts of interest to declare for other authors.

## Supporting information


**Table S1:** PRIOR statement.^1^
**Table S2:** Example database search strategy.
**Table S3:** Overview terminology and definitions.
**Table S4:** Traffic light coding of reviews.
**Figure S5:** Overlap between primary studies within reviews.
**Table S6:** CALO‐RE behavior change techniques and definitions.^9^
**Table S7:** AMSTAR‐2 quality appraisal.^10^
**Table S8:** Theoretical Domains Framework (TDF) coding (adapted from Heslehurst, 2014).^11^
**Table S9:** Characteristics amber and red reviews.
**Table S10:** Moderate, low and very low confidence review findings coded to TDF.
**Table S11:** Changes to protocol.

## Data Availability

The data that support the findings of this study are available from the corresponding author upon reasonable request.
