# Supplementary material for: The Influence of Relationships Within Behavioral Weight Management Interventions: An Overview
Source: Obes Rev. 2026 Jun 17;27(9):e70176. doi: 10.1111/obr.70176 (PMC13460655; doi:10.1111/obr.70176)
Supplement: Supplementary file 1 [file OBR-27-e70176-s001.docx]

**Supplementary Files**

The influence of relationships within behavioural weight management interventions: An Overview.

**Authors:** Claire E Torrens_1_, Pauline Campbell_2_, Scott Findlay_3,_ Julie Cowie_4_, , Ronan O’Carroll_5_, Pat Hoddinott_1_, Gozde Ozakinci_5_, Barbara Farquharson_1._

**Departments and Institutions:** 1. CHeCR, Faculty of Health Sciences and Sport, University of Stirling**,** 2. Department of Nursing, Community and Public Health, Glasgow Caledonian University**,** 3. University of the West of Scotland 4. Yunus Centre for Social Business and Health, Glasgow Caledonian University, 5. Division of Psychology, Faculty of Natural Sciences, University of Stirling.

Table S1 PRIOR Statement^1^

Table S2 Example Database Search Strategy

Medline (EBSCO)

1. MH Obesity[explode]

2. TX Obes* OR weight OR “Body Mass Index” OR BMI OR over-eat*

3. 1 OR 2

4. MH Interpersonal relations [explode]

5. TX Relation* OR Alliance OR Rapport

6. TX (Therap* OR Work*) n1 (relation* OR alliance)

7. TX (Weight OR patient-cent#red OR patient-provider) n2 communication

8. TX (Professional n1 Patient) n1 relation*

9. TX (Physician n1 Patient) n1 relation*

10. TX (Nurse n1 Patient) n1 relation*

11. TX (Researcher n1 subject) n1 relation*

12. TX (Researcher n1 Participant) n1 relation*

13. TX (Patient n1 clinician) n1 relation*

14. TX “Interpersonal relation*”

15. TX (Communication OR interpersonal) n2 style

16. 4 OR 5 OR 6 OR 7 OR 8 OR 9 OR 10 OR 11 OR 12 OR 13 OR 14 OR 15

17. MH Weight reduction programs [explode]

18. TX (Weight OR Obesity) n3 ((“Brief Intervention”) OR (Behavio#r n2 change) OR Lifestyle OR manage* OR self-care OR loss OR reduc* OR intervention OR Program*))

19. 17 OR 18

20. 3 AND 16 AND 20

Limited by year (1998-), English language and publication type (review, systematic review and meta-analysis).

Table S3 Overview Terminology and Definitions

| Term | Definition |
| --- | --- |
| Behavioural Weight Management | We use the term BWMI throughout to describe any intervention or programme that was delivered to promote behaviour change in physical activity and/or diet at the individual-level and/ or described behaviour change techniques. These could include brief one-off consultations with for example a healthcare provider, ongoing relationships providing weight management or studies or trials of weight management interventions. |
| Relationships | Our particular interest in relationships relates to developing an understanding of BWMIs beyond what is delivered toward how it is delivered (i.e. the “form of delivery”). Form of delivery has previously been described by Dombrowski et al (2016)^2^ and broadly defines important aspects of intervention delivery in terms of the provider, delivery format, materials, setting, intensity, tailoring and style. These features i.e. intervention setting, mode of delivery and style of delivery have been defined within the more recently developed Behaviour Change Intervention Ontology (BCIO)^3^. The BCIO enables a common descriptive language for key constructs within behaviour change interventions and incorporates separate ontologies for behaviour change techniques (BCT) and mechanisms of action (MoA). However, there is no singular definition for the construct of ‘relationship’ within behaviour change interventions. Therefore, to conceptualise and assist our interpretation of user-provider relationships within BWMIs we have used Bordin’s^4,5^ definition of the therapeutic or working relationship, also known as the working alliance. |
| User-Provider Relationships | To be inclusive of all providers of BWMIs we use the terms user and provider, referring to the person seeking or accessing help (user) and the person providing or giving access to help (provider) enabling relationships within a trial or study setting, for example the researcher-participant relationship, to be considered. |
| Working Relationship | The working relationship or alliance is a familiar concept within psychology and psychotherapy and incorporates various qualities including warmth, trust, rapport and collaboration. These qualities align to the person-centred approach developed by Carl Rogers more specifically the core conditions of empathy, congruence and unconditional positive regard^6^. Using this theoretical backdrop having the ability to understand the world through another person’s eyes, being genuinely involved and accepting and valuing a person are fundamental to relationship building. Bordin considered the working relationship applicable within any change-related relationship and it has been used to understand and evaluate relationships within other health settings such as primary care^7^ as well as in weight management^8^ . Bordin defined working relationships as having 3 component parts: i) mutual agreement of goals sought in the change process ii) the tasks of each of the partners in the relationship and iii) the bond (i.e. liking, caring, accepting, trusting) between partners necessary to sustain the change process. |

Table S4 Traffic Light Coding of Reviews

| Code | Definition |
| --- | --- |
| Red | Very little description of the user-provider relationship presented in the results/ discussion. There may be a reference to staff undertaking a task with participants but no description of the interaction between staff and participants. |
| Amber | Some or a reasonable amount of descriptive data on the user-provider relationship. Some findings reported in the results/ discussion that relate to the interaction and its features between staff and participants. |
| Green | A good amount of detailed in-depth description of the user-provider relationship presented in the results/ discussion. Describes the interaction, its features and form of delivery between staff and participants and may report the context or mechanisms of action. |

Figure S5 Overlap between Primary Studies within Reviews

Table S6 CALO-RE Behaviour Change Techniques and Definitions^9^

| BCT | Definition |
| --- | --- |
| 1. Provide information on consequences of behaviour in general | Information about the relationship between the behaviour and its possible or likely consequences in the general case, usually based on epidemiological data, and not personalised for the individual (contrast with technique 2). |
| 2. Provide information on consequences of behaviour to the individual | Information about the benefits and costs of action or inaction to the individual or tailored to a relevant group based on that individual's characteristics (i.e. demographics, clinical, behavioural or psychological information). This can include any costs/benefits and not necessarily those related to health, e.g. feelings. |
| 3. Provide information about others approval | Involves information about what other people think about the target person's behaviour. It clarifies whether others will like, approve or disapprove of what the person is doing or will do. |
| 4. Provide normative information about others behaviour | Involves providing information about what other people are doing i.e. indicates that a particular behaviour or sequence of behaviours is common or uncommon amongst the population or amongst a specified group presentation of case studies of a few others is not normative information. |
| 5. Goal setting (behaviour) | The person is encouraged to make a behavioural resolution (e.g. take more exercise next week). This is directed towards encouraging people to decide to change or maintain change. |
| 6. Goal setting (outcome) | The person is encouraged to set a general goal that can be achieved by behavioural means but is not defined in terms of behaviour (e.g. to reduce blood pressure or lose/maintain weight), as opposed to a goal based on changing behaviour as such. The goal may be an expected consequence of one or more behaviours, but is not a behaviour per se (see also techniques 5 (Goal setting - behaviour) and 7 (Action planning)). This technique may co-occur with technique 5 if goals for both behaviour and other outcomes are set. |
| 7. Action planning | Involves detailed planning of what the person will do including, as a minimum, when, in which situation and/or where to act. May describe frequency (such as how many times a day/week or duration (e.g. for how long). The exact content of action plans may or may not be described, in this case code as this technique if it is stated that the behaviour is planned contingent to a specific situation or set of situations even if exact details are not present. |
| 8. Barrier identification/problem solving | This presumes having formed an initial plan to change behaviour. The person is prompted to think about potential barriers and identify the ways of overcoming them. Barriers may include competing goals in specified situations. This may be described as “problem solving”. If it is problem solving in relation to the performance of a behaviour, then it counts as an instance of this technique. Examples of barriers may include behavioural, cognitive, emotional, environmental, social and/or physical barriers. |
| 9. Set graded tasks | Breaking down the target behaviour into smaller easier to achieve tasks and enabling the person to build on small successes to achieve target behaviour. This may include increments towards target behaviour or incremental increases from baseline behaviour. |
| 10. Prompt review of behavioural goals | Involves a review or analysis of the extent to which previously set behavioural goals (e.g. take more exercise next week) were achieved. In most cases, this will follow previous goal setting (see technique 5, goal setting-behaviour) and an attempt to act on those goals, followed by a revision or readjustment of goals, and/or means to attain them. |
| 11. Prompt review of outcome goals | Involves a review or analysis of the extent to which previously set outcome goals (e.g. to reduce blood pressure or lose/maintain weight) were achieved. In most cases, this will follow previous goal setting (see technique 6, goal setting-outcome) and an attempt to act on those goals, followed by a revision of goals, and/or means to attain them. |
| 12. Prompt rewards contingent on effort or progress towards behaviour | Involves the person using praise or rewards for attempts at achieving a behavioural goal. This might include efforts made towards achieving the behaviour or progress made in preparatory steps towards the behaviour, but not merely participation in intervention. This can include self-reward. |
| 13. Provide rewards contingent on successful behaviour | Reinforcing successful performance of the specific target behaviour. This can include praise and encouragement as well as material rewards but the reward/incentive must be explicitly linked to the achievement of the specific target behaviour i.e. the person receives the reward if they perform the specified behaviour but not if they do not perform the behaviour. This can include self-reward. Provisions of rewards for completing intervention components or materials are not instances of this technique. References to provision of incentives for being more physically active are not instances of this technique unless information about contingency to the performance of the target behaviour is provided. |
| 14. Shaping | Contingent rewards are first provided for any approximation to the target behaviour e.g. for any increase in physical activity. Then, later, only a more demanding performance, e.g. brisk walking for 10â€‰min on 3 days a week would be rewarded. Thus, this is graded use of contingent rewards over time. |
| 15. Prompting generalisation of a target behaviour | Once behaviour is performed in a particular situation, the person is encouraged or helped to try it in another situation. The idea is to ensure that the behaviour is not tied to one situation but becomes a more integrated part of the person's life that can be performed at a variety of different times and in a variety of contexts. |
| 16. Prompt self-monitoring of behaviour | The person is asked to keep a record of specified behaviour(s) as a method for changing behaviour. This should be an explicitly stated intervention component, as opposed to occurring as part of completing measures for research purposes. This could e.g. take the form of a diary or completing a questionnaire about their behaviour, in terms of type, frequency, duration and/or intensity. Check the distinction between this and techniques 17 (prompt self-monitoring of behavioural outcome). |
| 17. Prompt self-monitoring of behavioural outcome | The person is asked to keep a record of specified measures expected to be influenced by the behaviour change, e.g. blood pressure, blood glucose, weight loss, physical fitness. |
| 18. Prompting focus on past success | Involves instructing the person to think about or list previous successes in performing the behaviour (or parts of it). |
| 19. Provide feedback on performance | This involves providing the participant with data about their own recorded behaviour (e.g. following technique 16 (prompt self-monitoring of behaviour)) or commenting on a person's behavioural performance (e.g. identifying a discrepancy with between behavioural performance and a set goal see techniques 5 (Goal setting - behaviour) and 7 (action planning) or a discrepancy between one's own performance in relation to others note this could also involve technique 28 (Facilitate social comparison). |
| 20. Provide information on where and when to perform the behaviour | Involves telling the person about when and where they might be able to perform the behaviour this e.g. tips on places and times participants can access local exercise classes. This can be in either verbal or written form. |
| 21. Provide instruction on how to perform the behaviour | Involves telling the person how to perform behaviour or preparatory behaviours, either verbally or in written form. Examples of instructions include; how to use gym equipment (without getting on and showing the participant), instruction on suitable clothing, and tips on how to take action Showing a person how to perform a behaviour without verbal instruction would be an instance of technique 22 only. |
| 22. Model/Demonstrate the behaviour | Involves showing the person how to perform a behaviour e.g. through physical or visual demonstrations of behavioural performance, in person or remotely. |
| 23. Teach to use prompts/cues | The person is taught to identify environmental prompts which can be used to remind them to perform the behaviour (or to perform an alternative, incompatible behaviour in the case of behaviours to be reduced). Cues could include times of day, particular contexts or technologies such as mobile phone alerts which prompt them to perform the target behaviour. |
| 24. Environmental restructuring | The person is prompted to alter the environment in ways so that it is more supportive of the target behaviour e.g. altering cues or reinforcers. For example, they might be asked to lock up or throw away or their high calorie snacks or take their running shoes to work. Interventions in which the interveners directly modify environmental variables (e.g. the way food is displayed in shops, provision of sports facilities) are not covered by this taxonomy and should be coded independently. |
| 25. Agree behavioural contract | Must involve written agreement on the performance of an explicitly specified behaviour so that there is a written record of the person's resolution witnessed by another. |
| 26. Prompt practice | Prompt the person to rehearse and repeat the behaviour or preparatory behaviours numerous times. Note this will also include parts of the behaviour e.g. refusal skills in relation to unhealthy snacks. This could be described as “building habits or routines” but is still practice so long as the person is prompted to try the behaviour (or parts of it) during the intervention or practice between intervention sessions, e.g. as “homework”. |
| 27. Use of follow-up prompts | Intervention components are gradually reduced in intensity, duration and frequency over time, e.g. letters or telephone calls instead of face to face and/or provided at longer time intervals. |
| 28. Facilitate social comparison | Involves explicitly drawing attention to others performance to elicit comparisons. |
| 29. Plan social support/social change | Involves prompting the person to plan how to elicit social support from other people to help him/her achieve their target behaviour/outcome. This will include support during interventions e.g. setting up a “buddy” system or other forms of support and following the intervention including support provided by the individuals delivering the intervention, partner, friends and family. |
| 30. Prompt identification as role model/position advocate | Involves focusing on how the person may be an example to others and affect their behaviour, e.g. being a good example to children. Also includes providing opportunities for participants to persuade others of the importance of adopting/changing the behaviour, for example, giving a talk or running a peer-led session. |
| 31. Prompt anticipated regret | Involves inducing expectations of future regret about the performance or non-performance of a behaviour. This includes focusing on how the person will feel in the future and specifically whether they will feel regret or feel sorry that they did or did not take a different course of action. Do not also code instances of this technique as the more generic providing information on consequences (techniques 1 (provide information on consequences of behaviour in general and 2 (provide information on consequences of behaviour to the individual)). |
| 32. Fear arousal | Involves presentation of risk and/or mortality information relevant to the behaviour as emotive images designed to evoke a fearful response (e.g. “smoking kills!” or images of the grim reaper). Do not also code instances of this technique as the more generic providing information on consequences (techniques 1 (provide information on consequences of behaviour in general) and 2 (provide information on consequences of behaviour to the individual)). |
| 33. Prompt self talk | Encourage the person to use talk to themselves (aloud or silently) before and during planned behaviours to encourage, support and maintain action. |
| 34. Prompt use of imagery | Teach the person to imagine successfully performing the behaviour or to imagine finding it easy to perform the behaviour, including component or easy versions of the behaviour. Distinct from recalling instances of previous success without imagery (technique 18 (prompting focus on past success)). |
| 35. Relapse prevention/coping planning | This relates to planning how to maintain behaviour that has been changed. The person is prompted to identify in advance situations in which the changed behaviour may not be maintained and develop strategies to avoid or manage those situations. Contrast with techniques 7 (action planning) and 8 (barrier identification/problem solving) which are about initiating behaviour change. |
| 36. Stress management/emotional control training | This is a set of specific techniques (e.g. progressive relaxation) which do not target the behaviour directly but seek to reduce anxiety and stress to facilitate the performance of the behaviour. It might also include techniques designed to reduce negative emotions or control mood or feelings that may interfere with performance of the behaviour, and/or to increase positive emotions that might help with the performance of the behaviour. |
| 37. Motivational interviewing | This is a clinical method including a specific set of techniques involving prompting the person to engage in change talk in order to minimise resistance and resolve ambivalence to change (includes motivational counselling). |
| 38. Time management | This includes any technique designed to teach a person how to manage their time in order to make time for the behaviour. These techniques are not directed towards performance of target behaviour but rather seek to facilitate it by freeing up times when it could be performed. |
| 39. General communication skills training | This includes any technique directed at general communication skills but not directed towards a particular behaviour change. Often this may include role play and group work focusing on listening skills or assertive skills. |
| 40. Stimulate anticipation of future rewards | Create anticipation of future rewards without necessarily reinforcing behaviour throughout the active period of the intervention. Code this technique when participants are told at the onset that they will be rewarded based on behavioural achievement. |

Table S7 AMSTAR-2 Quality Appraisal^10^

| **Study ID** | **Q1.** | **Q2.** | **Q3.** | **Q4.** | **Q5.** | **Q6.** | **Q7.** | **Q8.** | **Q9.** | **Q10.** | **Q11.** | **Q12.** | **Q13.** | **Q14.** | **Q15.** | **Q16.** |
| --- | --- | --- | --- | --- | --- | --- | --- | --- | --- | --- | --- | --- | --- | --- | --- | --- |
| Ananthakumar 2020 | PARTIAL YES. No comparator group or timeframe re follow-up (Qual Review) Adults aged 18+ with overweight or obesity Clinician Consultation re weight management/ Primary Care WMP No Comparator Description of how excess weight was raised; thoughts and feelings about weight-related discussion | YES. Protocol was pre-registered on PROSPERO (CRD42015026734). | NO | PARTIAL YES. Search strategy in suppl files. Didn't justify publication restrictions. | YES. "two reviewers independently completed two rounds of screening, first at title and abstract and then full text stage." | YES. "Two reviewers independently extracted descriptive data and data relevant to quality of each study using an extraction form" | PARTIAL YES. Provided full text exclusions and reasons in PRISMA diagram. However, didn't provide full list of individual papers excluded. | PARTIAL YES. Was no comparator as Qual review and study design was only Qual studies. | YES. See Table 3 - used JBI checklist | NO. Not reported | No meta-analysis conducted | No meta-analysis conducted | NO. Quality assessment parag: “The studies were generally appropriately conducted (Table 3), although almost none commented on the context of the researcher or impact of the investigator on the study findings, hindering judgements of how the investigators may have influenced the results presented”. Not incorporated into the results of the review. | NO. Qualitative review | No meta-analysis conducted | YES. PA is a co-investigator on an investigator-initiated trial examining the effectiveness of a total diet replacement programme funded by Cambridge Weight Plan. PA has spoken at a clinical symposium on weight management funded by Novo Nordisk. Neither activity led to payments to him personally. All other authors have no other competing interests" |
| Dieterich 2020 | NO. Scoping review so eligibility criteria broad "(1) articles reporting on communication experiences and practices of healthcare providers when counseling overweight or obese pregnant patients, (2) peer reviewed (3) original research articles (4) published from 2008 to 2018 and (5) available in English. Exclusion criteria included articles that: (1) did not address communication practices or experiences of healthcare providers when counseling overweight or obese pregnant women, were (2) not peer-reviewed or original research, (3) were published before 2008 and (4) not available in English" | NO. Not reported | NO. Study designs not clearly reported in the inclusion criteria ("(2) peer reviewed (3) original research articles") | PARTIAL YES. "Searches using The Cumulative Index for Nursing and Allied Health Literature (CINAHL), Ovid, PubMed and Google Scholar electronic databases were conducted to identify relevant articles..., along with reference lists from reviewed studies were incorporated into the search strategy" | NO. Insufficient information ("Both authors (RD and JD) reviewed the full texts of the included articles with a specific focus on study design, sampling, methods, results and limitation") | NO. Insufficient information. Not clear who extracted the data ("Authors RD and JD jointly reviewed the table and individual studies using a modified approach to thematic analysis outlined by Braun and Clarke [29]. Specifically, they familiarized themselves with the data by reading each article and the table multiple times, searched for common topics (themes) addressed across included studies, and named and defined recurrent themes. Both authors discussed the themes and agreed on the final analysis") | PARTIAL YES. Excluded studies with reasons reported in PRISMA but not identified in separate tables | PARTIAL YES. Participant details were limited but evidence table organised by theme | NO. Scoping reviews do not usually conduct ROB. Authors report "Study quality was assessed by examining methodological rigor and the strengths and limitations of each study." No further detail reported. | NO. Not reported | No meta-analysis conducted | No meta-analysis conducted | NO. Indicated synthesis of search results involved: limitations/study quality however only reported limitations in table -quality not incorporated into interpretation. | NO. Not reported. | No meta-analysis conducted | YES. "Declaration of Competing Interest: None. Funding: This research was supported by funds from the Robert Wood Johnson Foundation Future of Nursing Scholars Program" |
| Henderson 2015 | YES. "English language studies published from 1997 to 2012 were included to examine recent perspectives. Only UK-based studies were examined to produce an in-depth understanding of the unique culture and health systems in the UK. Practitioners were those who deliver obesity services in primary care (healthcare and community settings). Patients were of any age.  Ethnographic accounts included semi-structured or unstructured interviews and focus groups, and participant observations. Surveys and structured  interviews were excluded. The desired final sample was one that provided both breadth (geography, participant, and service) and depth (descriptions of context) of topic" | NO. Not reported | YES. Ethnographic accounts  included semi- structured or unstructured interviews and focus groups, and participant observations. The desired final sample was  one that provided both breadth (geography,  participant, and service) and depth  (descriptions of context) of topic. | PARTIAL YES. Limited information about search presented ("PICOS terms were used to conduct keyword  searches: ‚primary care (Population); ‚obesity  services‚ (Intervention); ‚perspectives‚ (Outcomes); and ‚qualitative‚ (Study design). Databases  (MEDLINE¬Æ, Social Sciences Citation Index¬ CINAHL, and Health Management  Information Consortium), key academic journals, and bibliographic references were searched.") | NO. Single author review | NO. Single author review | PARTIAL YES. Excluded studies reported in PRISMA with reasons supplied but not presented in a separate table. | PARTIAL YES. Research design, populations (providers and patients), obesity services and perspectives described in some detail. Some comparison between patient and practitioner perspectives on roles and responsibilities - Box 1. | PARTIAL YES. Insufficient information ("Quality was assessed using the Critical Appraisal Skills Programme method.16") | NO. Not reported | No meta-analysis conducted | No meta-analysis conducted | NO. Just indicated all articles were assessed to be moderate to high quality - not included in interpreting or discussing results of the review. | NO. Meta-ethnography so heterogeneity not investigated. | No meta-analysis conducted | YES. "Competing interests: The author has declared no competing interests Funding: This work was funded with support from  the Wolfson Research Institute for Health  and Wellbeing, at Durham University" |
| Heslehurst 2014 | YES. Study population included HCPs Obesity or weight management relevant to pregnancy Perspectives No Comparator - qual review | NO. Not reported | YES. Primary research studies: research design included qualitative or survey methods (including quantitative or qualitative questionnaires) | YES. The electronic databases searched included AMED (1985‚ÄìJuly 2012), British Nursing Index (1992‚ÄìJuly 2012), CINAHL (1981‚ÄìJuly 2012), EMBASE (1988‚ÄìJuly 2012), MEDLINE (1946‚ÄìJuly 2012) and PsycINFO (1987‚ÄìJuly 2012). S2 MEDLINE search provided. Reference lists searched, Experts in the field of obesity, pregnancy and weight management (based on authors with a track record of publications in these fields) were contacted with requests for information on any studies relevant to the review aim. Relevant organizations contacted with request for information. Conducted 2012 published 2014. | YES. The titles and abstracts of all studies identified in the search were screened by two researchers independently (NH and CF). | YES. "Standardized data extraction templates were developed for qualitative and survey research, incorporating details on study context and design, participants, data analysis methods and main findings. Data extraction was carried out by two researchers independently: CF carried out data extraction for all studies, NH and JR carried out second data extraction (see Supporting Information Table S3 for example of data extractions)." | PARTIAL YES. Excluded studies reported in PRISMA diagram with reasons but cannot locate a table reporting these separately. | PARTIAL YES. Research design described study participants - mixed samples of HCPs, weight-related focus - GWG, maternal obesity, GWG and maternal obesity. | YES. "Standardized quality assessment templates were devel- oped using published quality criteria for qualitative and survey research (32,33). Quality assessment was carried out by four researchers independently: GM carried out quality assessments for all studies, NH and JR carried out second quality assessments. NH and/or JR were authors of four included studies (34‚Äì37), therefore an independent second quality assessment was carried out for these by JL (see Supporting Information Table S4 for full quality assessments). Studies were categorized as very good, good to very good, good or limited" | NO. No indication of how studies were funded. | No meta-analysis conducted | No meta-analysis conducted | NO. Statement: studies were categorized as very good, good to very good, good or limited. The limited quality in the reporting of the qualitative studies primarily related to a lack of detail on the methods employed and interpretation of the results, and in the survey studies a lack of detail on sampling and data analysis. The quality of the reporting of the majority of included studies was good (n = 13). A further five studies were considered to have very good reporting, and one had good to very good reporting. The quality of the reporting of six studies was deemed to be limited (qualitative studies n = 2, survey studies n = 3, and combined qualitative and survey. Quality not incorporated in interpreting/ discussing results. See "Table S4. Quality assessments, ‚Implementation of pregnancy weight management and obesity guidelines: a meta-synthesis of healthcare professionals barriers and facilitators using the Theoretical Domains Framework" | NO. Meta-synthesis so did not assess/ explain heterogeneity | No meta-analysis conducted | YES. "Conflict of interest statement: None." |
| Jeffers 2024 | YES. Population: HCPs Intervention: obesity management Comparator group: N/A Outcomes: perceptions and experiences  The SPIDER framework was chosen due to the qualitative nature of the research question. The SPIDER framework uses the same principals as the well-known PICO (Problem, Intervention, Comparison/Control, Outcome) framework but applies it to qualitative and mixed-method studies (Cooke et al., 2012). | NO. Cannot find reference to published protocol | YES. Only papers reporting qualitative findings using interviews or focus group methodologies were included as these were deemed most appropriate for analysing perceptions and attitudes of the sample population (Moule et al., 2016). Qualitative papers using surveys were excluded as surveys often do not gain adequate depth of understanding from participants compared to focus groups or interviews (Ring et al., 2011; Jones, 2013) | PARTIAL YES. CINAHL and Medline. Limited search strategy - no MeSH headings etc. Did not do any of the following:  -searched the reference lists / bibliographies of included studies - searched trial/study registries - included/consulted content experts in the field - where relevant, searched for grey literature - conducted search within 24 months of completion of the review | NO. "Inclusion criteria were applied to titles, abstracts and full paper. A second researcher screened 10% of titles, abstracts and full papers generated by the search". PRISMA not easy to follow. | NO. Data extraction limited; discussed coding "line by line" | NO. No details of the excluded studies in the PRISMA flowchart | PARTIAL YES. Table 4 summarises the results (aim/ sample/ design and results/themes identified) | PARTIAL YES. They used the CASP qualitative tool but did not provide any reasons for their judgements. | NO. Not described | No meta-analysis conducted | No meta-analysis conducted | NO. Limited ("The papers we identified for inclusion were of sufficiently high quality, illustrating how their data analysis was rigorously conducted and stating how themes or trends were identified and backed up by secondary researchers. The overall high quality of included papers lends cautious support to our synthesised findings and conclusions. However, it must be noted that many papers failed to outline the relationship between participant and researcher, which may contribute to bias in our findings. ") | NO. Not reported | No meta-analysis conducted | YES. "No funding was allocated for this research as it was undertaken as part of a dissertation" |
| Lee 2022 | YES. Eligibility criteria framed using PICO | YES. Protocol was registered in advance and is available in PROSPERO (CRD42020189897 | YES. "Individual or cluster RCTs of any behavioral (i.e., nonpharmacological or bariatric) intervention that aimed to support weight management (i.e., defined as weight maintenance or weight loss) through diet alone or diet and physical activity." | PARTIAL YES. Searched Medline for qual studies. Searched 4 databases for RCTs. No restrictions on qual or RCT search. Ref lists searched. Search strategy provided in appendices within 2 years. Trial registries not searched or grey literature. Content experts not consulted. | NO. Insufficient information "All studies identified were imported into Covidence for screening.30 After duplicates were removed, titles and abstracts were double-screened for eligibility. Discrepancies regarding study inclusion were resolved through discussion" | YES. "Data were double extracted by five researchers using a piloted form." | NO. Reasons for exclusion are reported in the PRISMA but no other table provided. | YES. Detailed in Table 2 and detailed in Appendix E | PARTIAL YES. | NO. Not provided | No meta-analysis conducted | No meta-analysis conducted | NO. Two separate reviews in this paper: one qual and one quant. ROB only conducted for Quant SR. "Sixteen studies were judged to be at high risk of bias.54,55,58,64‚Äì69,71,74,75,78,81‚Äì83 One study was judged to be low risk of bias overall.80 The remaining 17 studies were rated as unclear risk of bias.56,57,59‚Äì63,70,72,73,76,77,79,84‚Äì87 Table 2 lists summary risk of bias scores. Appendix H lists judgments by domain for each study | YES. Insufficient information ("there was marked heterogeneity. between outcomes, which is what we sought to investigate here. We focused on intervention characteristics that specifically addressed barriers that people with SMI have reported when engaging with BWMIs.") | No meta-analysis conducted | YES. "Funding information National Institute for Health Research (NIHR) Senior Investigator, Grant/Award Number: NF- SI-0617-10064; Wellcome Trust, Our Planet Our Health (Livestock, Environment, and People), Grant/Award Numbers: 102176/ B/13/Z, 205212/Z/16/Z; Oxford and Thames Valley NIHR Applied Research Centre (ARC), Grant/Award Number: 200172; Engineering and Physical Sciences Research Council (EPSRC), Grant/Award Number: EP/ R513295/1"..."The authors declare no conflict of interest. " |
| McHale 2016 | YES. Eligibility criteria clearly reported in Table 2 | NO. No indication of written protocol | YES. See Table 2 | PARTIAL YES. A systematic literature search of seven electronic online databases. No date restrictions were placed on the databases and only studies accessible in English were included in the systematic review. Database searches included all papers published up until August 2015. Search terms relating to direct observation methodologies, such as ‚video recording, were intentionally omitted from the search syntax because these terms are not routinely indexed in the electronic  online databases thesauri. Table 1 included search strategy. No trial registries, grey lit and expert not consulted. Searches 2015 published 2016. | NO. "CTM carried out all database searches and screened all citations by title and abstract. The review process adhered to PRISMA guidelines (30). All three reviewers (CTM, AHL and JEC) independently assessed relevant studies at full text level and their independent judgements were combined to determine inter-reviewer reliability" | NO. Insufficient information. Unclear who extracted the data and how many people were involved. | PARTIAL YES. Excluded studies reported in PRISMA with reasons but a list of individual studies was not listed | PARTIAL YES. Population, research design, , outcome described in some detail. All weight-related consultations in primary care. Follow-up periods indicated for cohort studies- 1 or 3 months | PARTIAL YES. A checklist tool, Health Evidence Bulletin Wales, was chosen to assess the quality of studies included in this review (34) due to its  specific design for use with observational research. The checklist contains 12 items that assess bias, design and use of analytical and statistical methods. Cohort studies were scored out of 12, whilst studies with other designs (cross-sectional, comparative case study) were scored out of 11. Scores were converted into percentages to allow comparison. | NO. Not reported | No meta-analysis conducted | No meta-analysis conducted | YES. Section in results discussed the quality assessment and also reported in the study limitations | NO. Did not discuss heterogeneity | No meta-analysis conducted | YES. "Funding: Review was carried out as part of a PhD funded by the University of St Andrews 600th Anniversary Scholarship. Ethical approval: Ethical approval was not required for this systematic review. Conflict of interest: The authors declare no conflict of interest." |
| Mold 2013 | YES. Patients living with obesity or Professionals providing care to patients living with obesity Healthcare services No comparator Experiences of delivery/ uptake of services | NO. No mention of a protocol | NO. Limited details provided for eligibility criteria (" The inclusion criteria were the following: the paper had to describe original research or a review of original research") | PARTIAL YES. "Electronic searches were undertaken using Medline, PubMed, CINAHL and the Social Science Citations. Index and free-text term searches included obesity (and related terms such as body size, body image, body weight, overweight), stigma, bias and discrimination, inequalities and access (and related search terms). Electronic database searches were limited by date ‚Äì including only literature published within an inclusive 20-year period (between February 1990 and February 2010) ‚Äì and by age, focusing on adults only. No limits were set for language or document type. These searches were supplemented with hand searches of key journals and open Internet searches. Citation searches (following key papers and authors) and secondary references were also undertaken" | NO. As such, a low level of suspicion was adopted to ensure maximum initial inclusion. Retrieved studies were initially reviewed by one author (FM), and final inclusion of each paper was discussed between authors (FM & AF). No indication or agreement. Not clear if completed independently. | NO. Indicates what was extracted but not how and by whom. A range of information was extracted from each of the studies including the core features of the studies and their findings related to the professionals and patients perspectives on the experiences of obese patients and the care they receive. | YES. Table 1 is a list of excluded studies with reasons | YESDetails provided across a series of evidence tables (see Tables 2-5) | PARTIAL YES. Each paper was critically appraised for the quality of the research using a broad critical appraisal process that considered the various research designs of each study.18,19 Key considerations at critical appraisal included methodological (validity, results and relevance) and analytical soundness (whether the analysis is consistent with the results) and considerations for minimizing research bias. | NO. No details provided for funding of individual studies. | No meta-analysis conducted | No meta-analysis conducted | NO. Unclear to see how the ROB was incorporated into the results (Each paper was critically appraised for the quality of the research..Notes were important in considering the validity and rigour of the identified studies.) | NO. Integrative review so did not talk about heterogeneity between studies | No meta-analysis conducted | NO. No reference to conflicts of interest/ funding |
| Obino 2016 | YES. General population Coaching No comparator weight loss  The objectives of this IR were to  relate ‚coaching‚ tools to weight loss and health and answer the following question: How can coaching assist in the process of weight loss and improve the state of health of the general population? | NO. No reference to a written protocol. | NO. Limited information for inclusion criteria | PARTIAL YES. "The search for indexed publications was conducted in February 2015 using the following databases: PubMed, Scientific Electronic Library Online, and Latin American and Caribbean Literature in Health Sciences. The  descriptors used were ‚coaching‚ and the keywords ‚weight loss,‚ ‚quality of life, ‚health promotion, and ‚neurolinguistic programming; AND was used as a Boolean operator between the descriptor and the keywords" | NO. Insufficient information | NO. Insufficient information | NO. No PRISMA and no list of excluded studies. However in the text authors state "We identified 289 articles about coaching from the literature search. Among them, 287 articles were identified from the PubMed database, of which 274 were excluded because they did not address the leading research question or their full texts were not available on the Internet. Two articles were identified in the Latin American and Caribbean Literature in Health Sciences database: one that had already been selected in the PubMed database, and one that did not meet the inclusion/exclusion criteria. No articles were found in the Scientific Electronic Library Online database. Thus, the review was conducted with the 13 articles that met the established criteria" | NO. Population not really described or outcomes. Research design provided and intervention type - in-person and mixed and duration of sessions. Very little detail. | NO. No quality assessment completed | NO. Not reported | No meta-analysis conducted | No meta-analysis conducted | NO. No indication that quality of studies was assessed. | NO. Integrative review - did not discuss heterogeneity. | No meta-analysis conducted | YES. "Disclosure: The authors report no conflicts of interest in this work" |
| Patel 2019 | YES. Eligibility criteria clearly reported in Table 1 | YES. The systematic review was conducted using a predetermined protocol registered with PROSPERO (CRD42018094147; https://www.crd.york.ac.uk/prospero/display_record.php?RecordID=94147 | YES. Studies were limited to randomized controlled trials, to enhance methodological quality. | PARTIAL YES. "The medical librarian (MBB) and the first author (MLP) constructed the search strategies. The major concepts searched were motivational interviewing, body weight, and technology and these combined concept searches were completed in four electronic databases: PubMed, Embase, PsycInfo, and CINAHL. See Supplemental Table 1 for the tailored search strategies. Hand-searching was conducted of the reference lists of included articles, relevant post-2009 systematic reviews (and relevant journals (Obesity, JMIR, Annals of Behavioral Medicine). This step was conducted to help further identify articles that may not have been captured in the database search" | YES. "two reviewers (MLP and LNW) independently screened all titles and abstracts of the remaining studies and classified papers as potentially eligible or not eligible. All papers deemed potentially eligible by at least one reviewer were then independently evaluated in the full-text review." | YES. " The first author (MLP) extracted data from all included trials using a standardized form. A second reviewer (LNW) verified the accuracy of this information." | Excluded studies reported in the PRISMA flowchart with reasons for exclusion but no separate table with individual studies presented | PARTIAL YES. All RCTs. Details of PICO described in detail but not doses or setting or timeframe for follow-up. | YES. "Two reviewers (MLP and LNW) independently assessed the risk of bias of each included trial, using a previously published adaptation of the Cochrane Risk of Bias Scale" | NO. No reference to funding of individual studies | No meta-analysis conducted | No meta-analysis conducted | NO. No trial had high risk of bias but included low and medium risk trials. Did not use this to interpret or discuss the results although it is mentioned. | NO. "Lastly, given the expected heterogeneity in intervention and study design, no meta-analysis was performed; nevertheless, this narrative synthesis suggests moderate efficacy of digital health interventions that incorporate MI for weight loss" | No meta-analysis conducted | YES. "Funding: This research did not receive any specific grant from funding agencies in the public, commercial, or not-for-profit sectors. Declaration of Competing Interest None" |
| Reading 2020 | YES. Eligibility criteria clearly described in a series of text sections in the narrative following PICO | YES. "This systematic review was developed using the Preferred Reporting Items for Systematic Reviews and Meta-Analyses (PRISMA) guidelines and registered with PROSPERO (ID#42017056566)" | YES. Studies using quantitative methodologies, such as RCTs and quasi-experimental pre-post studies, without a control group, were included. To limit the risk of bias, studies using only qualitative methods,  observational studies, secondary analyses, and posttest-only or pretest-only designs were excluded | PARTIAL YES. "To mitigate potential selection bias, the first author and an experienced librarian conducted an initial search on multiple online databases including  PubMed, Proquest, and Embase during February 2017....An additional search was conducted in July 2019 to ensure that no additional articles met inclusion criteria.... Keyword search terms included: education, medical education, primary care, training, workshops, physician‚ patient relations, doctor patient, clinician patient, communication, motivational interviewing, obesity, over-weight, weight loss, weight control, weight change, and weight management" Didn't contact experts or search grey literature, or trial registries. Conducted within 2 years. | NO. "The first author extracted data and reviewed the titles/abstracts for relevancy. Articles, titles and abstracts appearing to meet inclusion criteria were included for the second screening. Following the initial screening, the first and second authors independently reviewed the full text of articles using a prespecified protocol that was developed by all authors and included detailed inclusion criteria." | YES. "Each article was assigned an ID number. From there, we selected every third article for coding the initial dictionary. The first author reviewed 25% of the articles to establish an initial coding dictionary that captured the content of the training, including length, delivery, outcomes, and conceptual framework. Content of the methodology, including population and design, and results were coded. The first and second authors, then, reviewed articles using the coding dictionary to synthesize the articles, which involved combining the relevant content from each article that was reflective of the coding dictionary (length, framework, etc.). Authors names were removed prior to quantitative synthesis. The first and second authors quantitatively coded data using the coding dictionary. A kappa of .80 was achieved and  maintained throughout the coding process between the two independent coders" | PARTIAL YES. PRISMA shows 23 articles excluded after full-text screen with reasons and 9 articles excluded during data extraction - with reasons in the text ("9 were excluded for failing to meet inclusion criteria for the following reasons: (a) training was delivered to nurses and registered dietitians (n = 1); (b) training was specific to diabetes self-management (n = 2); and (c) trainings were targeted for the adolescent and/or pediatric patient  population (n = 6). " No separate table of individual studies excluded presented. | PARTIAL YES. Population, training intervention including dose and setting detailed. Comparator not described for both RCTs - referred to waitlist control for one study. Included studies reported in Tables 1 and 2 | NO. Unclear - appears that ROB conducted for RCTs only (n=2/7 included studies were RCTs) ("While a quality assessment was planned to assess the risk of bias and quality of reporting of the studies included using the Cochrane Handbook, the commonly validated tools to appraise individual studies were limited to randomized controlled trials (RCTs) [33]. Given that the studies extracted for this study are mainly a quasi-experimental design, assessing these studies using validated tools that are designed to assess the quality of RCTs has limitations in that many of the criteria are not applicable, thus, reducing the score in quality for each study." | NO. No indication of sources of funding for the studies included | No meta-analysis conducted | No meta-analysis conducted | NO. Did not assess quality in the end | NO. No reference to heterogeneity | No meta-analysis conducted | YES. "Funding: This research did not receive any specific grant from funding agencies in the public, commercial, or not-for-profit sectors...Conflicts of Interest: J.M.R., L.M.S., and J.G.L. declare that they have no conflicts of interest" |
| Ryan 2019 | YES. Overweight or obese free-living participants without specified clinical disease Tailored (behavioural) e-health interventions Control comparison group (generic or inactive) Weight loss | PARTIAL YES. Unable to access PROSPERO. However registered on PROSPERO CRD4201707290 | YES. The aim of the current paper was to review the evidence for tailored eHealth weight-loss interventions. We aim to describe in detail: 1. how tailoring was implemented in these studies and 2. whether these tailored approaches were effective in producing weight loss compared with generic or inactive controls | PARTIAL YES. Searches of all databases provided in Suppl files but also described in the text in addition to key word search, publication restrictions indicated (although no further explanation). Hand-searching completed, didn't contact experts or complete grey lit search. Searches re-run 2018 | NO. Not reported | YES. Data extraction was conducted by KR. Fifty per cent of the extracted data were checked for accuracy by author SD and 50% by author CL. However did not talk about consensus. | PARTIAL YES. Excluded studies reported in PRISMA flowchart with reasons but no table of excluded studies | YES. See table 1 - characteristic of included studies | PARTIAL YES. Quality of methodology was assessed by authors KR and SD using the Cochrane Collaboration tool for assessing risk of bias for RCTs.26 Bias was assessed as a judgement (high, low, or unclear) in the following domains of bias: selection, performance, detection, attrition, reporting and other bias. In this review, as the outcome of significance was weight, we included an assessment of measurement bias for weight as part of the quality review. Studies using self-reported weight, or using one-off (not averaged) weight measurements were considered to have an increased risk of measurement bias. Only assessed quantitative studies. | NO. Not reported | No meta-analysis conducted | No meta-analysis conducted | NO. Provided details of quality assessment of quantitative studies (6/8 studies) in paragraph and summarised in figure 2: There were two articles considered to be of low risk of bias, three of moderate quality, and one of high risk. Did not incorporate into discussion/ interpretation of the results. | NO. | No meta-analysis conducted | YES. "Funding: This work was supported by the Irish Research Council [grant number EPSPG/2016/182]. Conflict of interest: There are no conflicting interests related to this research" |
| Samdal 2017 | YES. Objectives were clearly stated. Eligibility criteria clearly reported on page 3 | YES. "The review was preregistered at PROSPERO with protocol and search strategy (CRD 42015020624" | YES. "Eligible study designs included published, peer-reviewed, randomized and cluster randomized controlled trials (RCTs) of behaviour change interventions providing baseline and/or follow-up data at minimum 12 weeks after randomization. The intervention duration was set at ‚â• 12 weeks to allow time for counselling to effect the behaviour change process" | PARTIAL YES. "The electronic databases MEDLINE, PsycInfo and EMBASE were searched in cooperation with the library service at the Medicine and Dentistry Library, University of Bergen, Norway. Articles published in peer-reviewed journals from January 2007 to April 2013 using a search strategy based on previous systematic reviews [10] with these adjustments were targeted; ‚Motiv* interview*‚ was added to the concept ‚psychological interventions, the search term ‚healthy eating‚ was added to ‚diet, and ‚physical activity‚ or ‚ walking were added to the term ‚exercise. Detailed search strategies can be obtained from the author. The initial search was updated once to October 2014. The reference list of relevant reviews on the topic of interest was also screened [19, 24‚ 33]. Additionally, we manually searched the following journals: International Journal of Obesity; International Journal of Behavioural Nutrition and Physical Activity; Obesity Research and Clinical Practice; and International Journal of Behavioural Medicine | YES. "The first 100 titles were screened in cooperation using a data collection form, and discussed by two reviewers (GBS and EM). In the next step, 100 titles were screened independently two separate times. This procedure yielded 94 and 90% agreement between the reviewers. Disagreements were solved through discussion. Thereafter, identifications of titles were performed by one researcher (GBS). The screening yielded 584 relevant titles of which abstracts were obtained. The first 20 abstracts were screened independently by two reviewers (GBS and EM). Thereafter GBS and EM independently screened all obtained abstracts." | YES. "Study and intervention characteristics were collected by GBS using two data collection forms and later checked by EM." | PARTIAL YES. Excluded studies reported in PRISMA with reasons for exclusion provided but no separate list of studies. | YES. See supplementary files | YES. GBS and EM independently assessed risk of under- or overestimating the intervention effects using a standard. risk of bias form covering: random sequence generation; allocation concealment; performance bias; blinding of assessment; attrition; and reporting bias [37] | NO. No indication of funding for included studies | Yes | Yes | YES. See section - Bias, BCTs and other study characteristics | YES. This was key objective of the paper - see supplementary files and narrative in text | Yes | YES. "Funding: The research project is funded by The Research Council of Norway (grant number 228454). Competing interests: The authors declare that they have no competing interests" |
| Skea 2019 | YES. Adults with BMI ‚a 35kg/m2 (and/or the views of providers involved in their care) Lifestyle WMPs No comparator Views of adults/ providers | YES. This study is registered as PROSPERO CRD42016040190. All information in protocol/Avenell (2018) Protocol: Plan provided for synthesis: During Qualitative Cycle C, Category C (non-intervention studies) will be theoretically sampled and analysed to further refine and validate our emerging analysis and theory. | YES. We included studies that fitted into the following broad categories: 1. Qualitative and mixed-methods studies linked to eligible randomised controlled trials (RCTs) (from our other review), including any qualitative data reported  as part of papers reporting quantitative outcomes. 2. Qualitative and mixed-methods studies linked to ineligible RCTs and identified non-randomised intervention studies including any reported qualitative data. 3. Qualitative studies not linked to specific interventions that drew on the experiences and perceptions of adults with BMI ‚â•35kg/m2 (and/or providers involved in their care) providing they reported data specifically relating to views/experiences of strategies for weight loss. | YES. 14 databases searched. Key word and search strategy provided. Trial registries, advisory group contacted. Reference lists of included studies searched. A systematic search was conducted for published papers that contained qualitative data from adults with BMIs of ‚â• 35 kg/m2 (and/or the views of providers involved in their care) and considered issues relating to weight management. Studies conducted in developing countries were included if they were relevant to the UK context.  The search strategies comprised two facets: severe or morbid obesity and qualitative studies. No date restrictions were imposed. Search updated 2017 and published 2018 and subsequently in 2019. | YES. Two researchers independently screened titles and abstracts and selected full-text papers. When consensus could not be reached regarding eligibility, a discussion took place at a research team meeting | YES. To collate and synthesise the available primary research, two authors (ZS and MAM) each read and systematically extracted data from the included papers, shared notes and discussed study findings and interpretations during a series of group meetings. The papers were initially organised in accordance with the categories described above but, as inductive analysis progressed, papers were grouped and compared and contrasted in accordance with emerging issues and themes. We used a standard data extraction form, which summarised the main themes, information regarding aims and methods and any other important information relating to the context of the research within each study in the qualitative systematic review. | YES. List of excluded studies with reasons in suppl file 1 - Avenell Report | PARTIAL YES. Populations, WMPs described, no comparators as views studies. Research design described - all qual and mixed methods studies | YES. "The retrieved publications were appraised for method- ological rigour and theoretical relevance independently  by two reviewers using Toye‚ recently proposed criteria  for quality in relation to meta-ethnography.16 They suggest two core facets of quality for inclusion in syntheses of qualitative evidence, namely (1) conceptual clarity: how clearly has the author articulated a concept that facilitates theoretical insight; (2) interpretive rigour: what is the context of the interpretation; how inductive are the findings; has the interpretation been challenged? Two reviewers made notes regarding quality and results were compared and discussed." | YES. Indicated that funding of studies was extracted as part of extraction form but not reported. | No meta-analysis conducted | No meta-analysis conducted | YES. Applying quality criteria to qualitative research remains a contentious issue and there is no consensus regarding whether and how this should be done.52 53 While authors of some qualitative evidence syntheses have chosen to exclude what they deem to be poor quality papers, we made the decision not to exclude any of the identified papers. We included 33 papers that each reported some qualita- tive data that met our inclusion criteria and addressed our key research questions. Although all included qualitative data, with regard to ‚quality, some were deemed richer than others in terms of data and insights. Some ranged from being exclusively qualitative studies providing rich data in our areas of interest, through to studies that were actually primarily quantitative with responses to open-ended survey questions. The five studies providing qualitative data in the form of responses to open-ended survey  questions within structured questionnaires22 32 37 46 49 were  deemed less useful as they presented only very limited qualitative data and insights. Despite this variation in the overall level of quality, we believed it was more important to retain any relevant findings rather than disregard based on study quality. In doing so, we would argue that all 33 papers contributed useful elements to the collective whole and enabled us to develop our understanding of the issues of importance to people with BMI ‚≥35kg/m2. We cannot exclude the possibility that unpublished service evaluations from within the NHS, that we failed to locate, might have been sources of rich data | YES. No significant heterogeneity described as views-based studies. | No meta-analysis conducted | YES. "Funding The project was funded by the NIHR Health Technology Assessment Programme (Project number: 15/09/04). See the HTA Programme website for  further project information. Competing interests None declared." |
| Sturgiss 2021 | YES. Adults 18+ with obesity delivered in primary care Therapeutic alliance in lifestyle interventions Both control (conventional health care) and alternative intervention (variation on the intervention) will be included. Reduction in body weight (kilograms) or reduction in BMI (%). Loss to follow-up | YES. The protocol of this systematic review was published prospectively and registered in PROSPERO (CRD42018091338). Meta-analysis will be performed if studies of sufficiently homogenous primary outcome data are found. Homogeneity will be determined statistically via I¬≤ (classified as negligible (I¬≤ = 0%), minimal (I¬≤ < 20%), moderate (20% < I¬≤ < 50%), or substantial (I¬≤ >50%)), as well as descriptively focusing on methodological and clinical variation. In instances in which studies are too heterogeneous to combine quantitatively, a narrative synthesis will be done. Change from baseline data will be used, otherwise endpoint data will be used. For variables with the same scale, weighted mean different with 95% CI will be used; with a different scale standardised mean different with 95% CI will be calculated. All pooling of data will use the DerSimonian and Laird random effects model. This reflects the diverse and complex nature of the interventions included. | YES. Controlled trials of interventions (randomised controlled trials (RCTs) and cluster RCTs) This study aimed to answer: 1. What effect does therapeutic alliance have on the effectiveness of  interventions for obesity in adults in primary care? 2. What effect does therapeutic alliance have on the rates of loss to  follow-up in obesity management trials in primary care? | PARTIAL YES. Databases to be searched: MEDLINE, EMBASE, PsycINFO, CINAHL, the Cochrane Central Register of Controlled Trials (CENTRAL), and Scopus. Both original literature searches plus citation searching will be conducted.  In addition, authors of unpublished trials that are registered with the World Health Organisation International Clinical Trials Registry Platform will be contacted. Theses databases to be searched: Open Access Theses and Dissertations and Networked Digital Library of Theses and Dissertations. Search strategy provided Does not indicate when searches were carried out. Protocol indicates searches until 2018 - published 2021 | YES. Data were independently identified by ES, NE and SA.  Protocol: Two reviewers will screen all titles and abstracts independently using Covidence software. Conflicts will be resolved by discussion, with studies retained for full text screening if agreement is not reached. Where there is ambiguity, abstracts will be retained for full text assessment. Where no abstract is available, records will be retained for full text assessment unless they can be judged from the title not to relate to the review topic. Full text screening: Two reviewers will assess full texts of all potentially eligible studies using a piloted checklist. Disagreement about eligibility will be resolved by discussion, involving a third reviewer where necessary. | YES. Data were extracted by KO and checked by SA. Two members of the research team then independently categorized interventions into three groups  Protocol: Two reviewers will extract data, independently, using piloted data extraction forms | PARTIAL YES. Numbers of excluded studies and reasons provided in PRISMA - not by individual study | PARTIAL YES. Population described, outcomes and intervention arms with comparators. All RCTs. Setting was primary care. Doses not described. Time to follow-up in supplementary file | YES. The quality of included studies will be assessed by two reviewers, independently using the risk of bias criteria detailed in Cochrane Collaboration's risk of bias tool. Risk of bias relating to the primary outcome will be assessed and presented for each study in a risk of bias table in suppl file | NO. No reference to funding of individual studies | No meta-analysis conducted | No meta-analysis conducted | NO. Did refer to risk of bias in paragraph and table provided in suppl file however did not discuss in relation to the results. | YES. Protocol: Homogeneity will be determined statistically via I¬≤ (classified as negligible (I¬≤ = 0%), minimal (I¬≤ < 20%), moderate (20% < I¬≤ < 50%), or substantial (I¬≤ >50%)), as well as descriptively focusing on methodological and clinical variation.  In instances in which studies are too heterogeneous to combine quantitatively, a narrative synthesis will be done.  As I2 was commonly over 90%, we are not able to make rigorous comparison between the studies regarding effectiveness.  The reported primary outcomes varied greatly (Table 1), and this contributed to the high statistical heterogeneity. The difficulties associated with variability in effectiveness outcomes have been recognized and the COMET initiative is working to develop agreed minimum outcomes for a variety of health conditions.  Subgroup analyses (time intensive- ness, whether the intervention was delivered in regular practice and usual provider involvement) were also not possible due to the small number of studies identified and high heterogeneity. | No meta-analysis conducted | YES. Authors made declaration of no actual or potential conflicts of interest. |
| Sutcliffe 2018 | YES. Adult (≥18 years) service users who had experience of attending a WMP Weight Management Programmes (WMP) No comparators (Qual synthesis) Service-users views, perceptions or beliefs about WMPs. | PARTIAL YES. Protocol for review 2 (Johns et al. 2013) agreed with NICE before beginning review. Sutcliffe (2016) based on this with update. The detailed search strategy was agreed separately between reviewers and the CPHE information specialist, and is reported in Appendix 2. We used the same electronic databases as we searched in  Review 1 (Medline, Medline in Process, Embase, Psycinfo, Cochrane (CENTRAL, DARE, CDSR), Science Citation Index, Conference Proceedings Citation Index), with the exception of BIOSIS, which was  judged not to be applicable to the questions in review 2. We used similar terms to those used in the Review 1 search but removed the filters that aimed to confine the search to randomised controlled trials and included terms to pick up specific keywords and text words. Table 1 provides criteria for inclusion. | YES. To develop a deeper understanding of WMPs critical features, we under- took a systematic review of qualitative evidence | PARTIAL YES. Since a recent NICE (2013) review (Johns 2013 Review 2 - part of NICE reviews) had undertaken searches for views studies,18 we assessed each of the qualitative studies included in that review. In addition, to identify qualitative studies conducted since that review was undertaken, we reran the search strategy employed for the NICE review on the highest yielding databases from their original search (MEDLINE, EMBASE, Web of Science, Medline in process) for the period from 2012 to 2014. To supplement the updated search, we also identified studies from other recent reviews of qualitative views research,20-22 conducted on all studies meeting our inclusion criteria and contacted key authors in the field. Trial registries not searched/ google search completed. Searches provided in Sutcliffe (2016)/ Johns (2013 - Review 2) | YES. Sutcliffe (2016) Titles and abstracts returned by the search strategy were exported into EPPI-Reviewer 4 (Thomas et al., 2010) reviewing software and independently screened by pairs of reviewers (KS, RR, MR) using the predefined criteria specified in Table 6.1. All disagreements were resolved by discussion between the reviewers. Where it was not possible to decide on the exclusion of a paper based on the information in the title and abstract, the full text was retrieved. The same three researchers examined these independently for inclusion or exclusion using modified predefined criteria | YES. Sutcliffe (2016) Data were extracted from studies meeting the eligibility criteria for inclusion in the syntheses. User and provider views were extracted independently, leading to the development of two classification systems. | PARTIAL YES. Johns (2013) provides list of excluded studies and reasons (26 references and 25 studies) but in Sutcliffe (2016) doesn't (26 qual studies and 31 reports). Sutcliffe (2018) - 21 studies in 26 papers (only service-users views studies) | PARTIAL YES. Described service-users, intervention and outcomes in detail as well as design of qual studies. Table 2 provides an overview. | NO. Did not assess quality. To ensure a basic level of quality, papers were excluded if they (i) did not provide a clear account of the methods used for data collection and analysis; and (ii) contained only minimal or data pertinent to the review question. | NO. No indication that this was reported. | No meta-analysis conducted | No meta-analysis conducted | YES. There is a lack of consensus among qualitative researchers about how to measure quality in qualitative research;23 therefore, we were cautious about excluding papers on the basis of quality. Nonetheless, to ensure a basic level of quality, papers were excluded if they (i) did not provide a clear account of the methods used for data collection and analysis; and (ii) contained only minimal or ‚data pertinent to the review question. | NO. Heterogeneity not described as qual synthesis. | No meta-analysis conducted | YES. Authors declared no competing interests |
| Teixeira 2012 | YES. General and Family Practitioners Healthcare provider obesity practices Beliefs, attitudes and practices No comparator as qual review | NO. No indication of a written protocol | NO. Inclusion criteria only states empirical evidence | PARTIAL YES. Searched several databases and provided key word search. Publication restrictions discussed: material written in English, Portuguese and Spanish between 1991 and 2011... No studies written in Portuguese were found and only one study was written in Spanish. Nevertheless, the latter did not meet the criteria, so the search methods had to be limited to studies published in English. Citation searching completed. | NO. No indication of methods of study selection. | NO. No indication of how data was extracted or by whom. | NO. No details of excluded studies provided | PARTIAL YES. Research design described in some detail and population. Beliefs, attitudes and practices detailed (Intervention and outcome). | NO. No indication that quality of studies was assessed. | NO. No details of funding for individual studies provided. | No meta-analysis conducted | No meta-analysis conducted | NO. Quality of studies not completed | NO. Integrative/ narrative review | No meta-analysis conducted | NO. No statement declaring no completing interests. |
| Wadden 2014 | YES. We used the PICOTS12 (ie, population, interventions, comparators, outcomes, timing, setting) approach to establish inclusion and exclusion criteria   Overweight or obese adults whose body mass index (BMI) was 25 or higher [BMI is calculated as weight in kilograms divided by height in meters squared] recruited from primary care setting Behavioural weight loss counselling/ lifestyle intervention (3 components diet, exercise and behavioural strategies) Delivered by CMS-defined PCP working alone or with trained interventionists or trained interventionists (not recognised by CMS) without PCPs remotely or in person. Comparator intervention Weight (Kg, BMI, %) Offered behavioral counseling for at least 3 months and with at least 6 months‚Äô follow-up after randomization; | NO. Cannot see any reference to a protocol | YES. This systematic review summarizes the results of randomized controlled trials involving patients recruited from a primary care setting, in which CMS-defined primary care practitioners,working alone or with trained interventionists, delivered behavioral weight loss counseling. It also examines randomized trials involving patients recruited from primary care who were counseled by trained interventionists alone, working without primary care practitioners, who delivered treatment in person, by telephone, and via the Internet rather than counseled by physicians.8 These interventionists are not currently recognized by the CMS as independent providers of behavioral counseling, although they may potentially provide services incident to eligible practitioners | PARTIAL YES. ".. and searched PubMed, CINAHL, and EMBASE from January 1, 1980, through June 30, 2014, using the following terms: obesity, primary care, weight loss, counseling, diet, exercise, behavior modification, and lifestyle counseling....It was supplemented by examination of prior reviews 6,13-15 and a search of the Cochrane Central Registry of Controlled Trials" | YES. "Titles and abstracts of all articles were reviewed independently by pairs of authors (T.A.W. and P.S.H.; M.L.B. and A.G.T.) to exclude non relevant articles. The full text of each remaining article was similarly reviewed to determine whether it met inclusion and exclusion criteria." | NO. Not reported | PARTIAL YES. Shown in the eFigure but no separate list of excluded studies | YES. Participants described in detail in the text and in the tables. Tables describe follow-up period, number of sessions, comparators and outcomes which is further elaborated on within the text in the results section. Setting was primary care and research design RCTs. | NO. As shown in the eFigure in the Supplement, 27 studies,16-42 3 of which published additional follow-up data,43-45 met all criteria and were subjected to a quality rating of poor, fair, or good by 2 authors who used criteria similar to those of the obesity guidelines.7 NHLBI Quality Rating of the Strength of Evidence - Table 3 (Jensen, 2013). | NO. Not reported | No meta-analysis conducted | No meta-analysis conducted | YES. "The 12 remaining studies, all with a good rating, were divided into 2 categories following preliminary examination" | NO. No discussion of heterogeneity, even to explain why they didn't combine data. | No meta-analysis conducted | YES. "Conflict of Interest Disclosures: All authors have completed and submitted the ICMJE Form for Disclosure of Potential Conflicts of Interest. Dr Wadden reported that he serves on advisory boards for Nutrisystem, Novo Nordisk, Orexigen, and Shire Pharmaceuticals; has received grant support, on behalf of the University of Pennsylvania, from these organizations and from Weight Watchers International; served as an advisor to Johns Hopkins University on a proprietary weight loss program (Innergy); and receives royalties from Guilford Press. Dr Butryn reports that she has received grant support, on behalf of Drexel University, from Shire Pharmaceuticals. Ms Hong reported no disclosures. Dr Tsai reported the he has received donations of food products from Nutrisystem for a research study. Funding/Support: Dr Wadden work on this project was supported, in part, by grant DK-65018 from the National Institute of Diabetes and Digestive and Kidney Diseases" |
| Warr 2021 | YES. "We included studies which were carried out in primary care, reported the perspectives of primary care staff in discussing overweight and obesity with adults (18+ years) who were overweight and included qualitative studies based on interviews or focus groups in which GPs and nurses reported their views about discussing overweight" | PARTIAL YES. Indicated they wrote a protocol which was supplied to the editor of the journal. No further details. | YES. Authors state in the introduction that "We therefore aimed to provide a comprehensive overview of GPs' and nurses' views of treating obesity in primary care using broader selection criteria than either of the other published reviews" | PARTIAL YES. "An initial search of the electronic databases PubMed, Embase, Cinahl, Web of Science and PsycINFO was undertaken" but no grey literature and not clear that they contacted experts/ | YES. "Title and abstract screening was conducted by two independent reviewers (WW and CA) using Covidence. In the event of disagreement, the article proceeded to the next phase.17 Full text screening was done by WW, and two 10% samples were checked by other authors." | NO. Not reported | PARTIAL YES. Full text papers with reasons for exclusions shown in PRISMA | PARTIAL YES. Does not provide extensive details of populations (601 GPs and nurses), interventions delivered, or outcomes. However details including methodological design, are provided in the tables. Setting was primary care. | YES. See Quality assessment (Table 2). ROB using JBI checklist | NO. Not reported | No meta-analysis conducted | No meta-analysis conducted | YES. Based on the results of this checklist, we assessed whether the ‚higher-quality studies contributed richer data to the thematic analysis We found that there was no such relationship and therefore included all papers.17  There were some quality issues. Some studies did not report ethical approval11,24; authors drew conclusions that did not flow from the data25 or did not discuss reflexivity.26,27 Recruitment to the study group was sometimes self-selected from a subgroup with training in weight management28‚Äì30 or recruitment was incentivised by offering educational obesity treatment training,27 which perhaps meant that clinicians with greater interest were recruited. | NO. Qual review heterogeneity not discussed | No meta-analysis conducted | YES. "PA obtained funding from Cambridge Weight Plan for an investigator-initiated clinical trial. PA spoke at a symposium at the Royal College of General Practitioners of the UK National Conference that was funded by Novo Nordisk. PA and CA did half a day's consultancy for Weight Watchers. None of these activities resulted in personal payments" |
| Whitehall 2025 | YES. Detailed in full in inclusion criteria section | YES. The protocol is registered in PROSPERO [CRD4202125343 | YES. Study design. Mixed-methods, quantitative and qualitative study designs where barriers and facilitators to engaging in a synchronous, interactive, remotely delivered weight management intervention were measured/discussed | YES. 12 electronic databases searched, search strategy provided. English language restrictions indicated. Reference lists hand searched. Clinical trials registry and grey literature searched. Consulted content experts re searches. Searches within 2 years (2023 published 2025) | YES. "evenly distributed between all authors and were double-screened for eligibility using the inclusion and exclusion criteria, with any discrepancies discussed and resolved by three authors .. The full-text screening was conducted similarly, with discrepancies resolved by three authors)" | YES. "completed by one researcher (EC) and checked by JMW" | NO. | YES. Population, intervention and outcomes (B/F engagement) clearly described in tables of characteristics - use of TiDIER and throughout results section with comparators described and setting (remote) and follow-up. | YES. Clearly detailed in Table 1 | NO. Not reported | No meta-analysis conducted | No meta-analysis conducted | NO. Reported but not incorporated in to interpreting/ discussing results | NO. Did not discuss heterogeneity or how dealt with it i.e. because this was qualitative drawing together results regarding engagement from across study designs. | No meta-analysis conducted | YES. Authors described funding. Authors declared no competing interests |

Abbreviations: Q1. Did the research questions and inclusion criteria for the review include the components of PICO?; Q2. Did the report of the review contain an explicit statement that the review methods were established prior to the conduct of the review and did the report justify any significant deviations from the protocol? Q3. Did the review authors explain their selection of the study designs for inclusion in the review? Q4. Did the review authors use a comprehensive literature search strategy?; Q5. Did the review authors perform study selection in duplicate?; Q6. Did the review authors perform data extraction in duplicate?; Q7. Did the review authors provide a list of excluded studies and justify the exclusions? Q8. Did the review authors describe the included studies in adequate detail?; Q9. Did the review authors use a satisfactory technique for assessing the risk of bias (RoB) in individual studies that were included in the review? Q10. Did the review authors report on the sources of funding for the studies included in the review?; Q11. If meta-analysis was performed did the review authors use appropriate methods for statistical combination of results?; Q12. If meta-analysis was performed, did the review authors assess the potential impact of RoB in individual studies on the results of the meta-analysis or other evidence synthesis?; Q13. Did the review authors account for RoB in individual studies when interpreting/ discussing the results of the review?; Q14. Did the review authors provide a satisfactory explanation for, and discussion of, any heterogeneity observed in the results of the review? Q15. If they performed quantitative synthesis did the review authors carry out an adequate investigation of publication bias (small study bias) and discuss its likely impact on the results of the review?; Q16. Did the review authors report any potential sources of conflict of interest, including any funding they received for conducting the review?

Table S8 Theoretical Domains Framework (TDF) Coding (Adapted from Heslehurst, 2014)^11^

| TDF Domain | Definition | Examples of Relevant Data |
| --- | --- | --- |
| (1) knowledge | An awareness of the existence of something | What did they say about having/not having/wanting knowledge of when and how to do the behaviour.  What did they say about having/not having/wanting an understanding of the rationale behind performing the behaviour.  What did they say about having knowledge of obesity, weight management, risks, contradictory beliefs. |
| (2) skills | An ability or proficiency acquired through practice | How did they describe the techniques/capability/skills used/how they do the behaviour in practice  What did they say about wanting to develop/improve skills  What did they say about training/ education (or lack of)  What did they say they did they do when uncertain of skills (e.g. reframing, link to other health conditions)  Did they say anything about how information or behaviour change could be approached relevant to skills of professional? E.g. active listening skills  the interpersonal nature of conducting the behaviour with the patient (e.g. discussing weight management strategies, informing of obesity status, difficulties) would be classed as skills. |
| (3) social/professional role and identity | A coherent set of behaviours and displayed personal qualities of an individual in a social or work setting. | How did healthcare professionals see themselves (own weight, gender and the impact on providing weight management)  How did patients/ participants see HCPs and their role  What was their view of weight management or behaviour change as a characteristic/ feature/ meaningful aspect/ representative of their professional role (or not) (e.g. recognition of importance)  Did they say anything about the extent their personal/ professional identity influences doing the behaviour  Also include:  beliefs/ attitudes of HCPs in general (e.g. professional ambivalence and the impact, trust or belief in the person).  Personal/ Individual/ social responsibility – u the role/ responsibility of the individual within society with regard to weight management (role/ identify of users).  References to professional confidence coded as belief about capabilities. |
| (4) beliefs about capabilities | Acceptance of the truth, reality, or validity about an ability, talent, or facility that a person can put to constructive use. | What did they say about confidence, judgements about their/ or others (HCP/ patient) competence and control in their ability (or inability) to perform the behaviour.  What did they say about their expectations of carrying out a behaviour due to beliefs of competency in performing the behaviour. |
| (5) optimism | The confidence that things will happen for the best or that desired goals will be attained | What did they say about optimism/pessimism of effectively discussing obesity, weight management, nutrition etc or of weight management/ behaviour change  Did they say anything about their hopes of a positive outcome, this could include their beliefs that HCPs are acting in their best interests or HCPs beliefs of a positive effect. |
| (6) beliefs about consequences | Acceptance of the truth, reality, or validity about outcomes of a behaviour in a given situation | What did they say about beliefs/views etc on the outcome/consequences of doing/not doing a behaviour/ carrying out weight management (positive or negative consequences).  Statements can include consequences of doing/not doing the behaviour on themselves or their patients.  Statements relating to doing the behaviour being directly contingent on receiving rewards or punishments.  What did they say about willingness to perform the behaviour based on expectations of outcomes (e.g. weight management in pregnancy is pointless/too late to reduce risks etc).  This could also include expectations about what will happen when engaging with services/ intervention/ HCPs |
| (7) reinforcement | Increasing the probability of a response by arranging a dependent relationship, or contingency, between the response and a given stimulus | What did they say about expectations in relation to service or intervention received – reinforcement or punishment. |
| (8) intentions | A conscious decision to perform a behaviour or a resolve to act in a certain way | Could include approaches such as motivational interviewing used by providers which aims to address intention and motivation.  Intentions expressed by providers or users |
| (9) goals | Mental representations of outcomes or end states that an individual wants to achieve | What did they say about prioritising weight management intervention/ consultation (importance of the task or goals of intervention/ routinely part of their role (goal of treatment)  This could include the broader goals or tasks of the interaction as well as specifically agreeing goals or plans for behaviour changes. |
| (10) memory, attention and decision processes | The ability to retain information, focus selectively on aspects of the environment and choose between two or more alternatives | Statements relating to time/situations etc when the healthcare professionals would remember or forget to do the behaviour.  Statements relating to relying on cognitive approaches to perform the behaviour/make a quick decision.  Providing treatment options or choices or making choices with or for users. |
| (11) environmental context and resources | Any circumstance of a person’s situation or environment that discourages or encourages the development of skills and abilities, independence, social competence, and adaptive behaviour | Describing the presence or absence of tools/resources/ equipment/ services/ organisational structures which facilitate/impede performing the behaviour  Describing how the organisational practice/culture/ population facilitates/impedes performing the behaviour  Wanting tools/resources/equipment/services/changes in the organisational structure to facilitate performing the behaviour  Having to prioritise behaviours due to time restrictions/ not having time would be coded as goals. |
| (12) social influences | Those interpersonal processes that can cause individuals to change their thoughts, feelings, or behaviours | What did they say about the influence of others on carrying out a behaviour (social support, group norms etc)  When healthcare professionals want patient perspectives, feedback, and experiences to influence their behaviour (e.g. healthcare professionals want feedback on patient experiences of obesity communication to help develop their communications skills etc).  What did they say about beliefs/ attitudes/ prejudice toward particular groups – stigma/ stereotypes. |
| (13) emotion | A complex reaction pattern, involving experiential, behavioural, and physiological elements, by which the individual attempts to deal with a personally significant matter or event | Expressing how their emotional reaction/state positively or negatively impacts on them doing the behaviour  emotion relates to the emotional response of the healthcare professional in relation to performing the behaviour, and the emotional response of the patients to the behaviour. |
| (14) behavioural regulation | Anything aimed at managing or changing objectively observed or measured actions | What did they say about behaviour change (techniques) used or not used (e.g. monitoring, prompts) in relation to supporting weight change  Did they say anything about helping/ not helping to make a behaviour sustainable/ routine/ habit. |

Table S9 Characteristics Amber and Red Reviews

| Characteristics of Amber Reviews | | | | | | | |
| --- | --- | --- | --- | --- | --- | --- | --- |
| Author(s), Year,  Setting,  Synthesis Type,  No. Studies | Population/ Participant Description (n) | BWMI Description | Mode of delivery | Relationship Described | Perspective and/or Outcome | Retention (e.g. attrition, engagement) | Conclusion |
| Alnaabi^12^  2021  NR  SR-QUAL  9 Studies | Adults  (n=936) | Mobile interventions utilising persuasive technology for obesity | Mobile telephone | NS | Features and possible gaps of mobile obesity interventions | Persuasive features associated with dialogue support similarly supported in current day mobile interventions for obesity. The most common persuasive features are liking, suggestions, and reminders, with eight instances each. It is important for interventions to be visually appealing in order to encourage compliance and engagement.  We found that current mobile obesity interventions have strong primary task and dialogue support. We also found that social support is much less supported, despite persuasive design research that stated social support is vital in creating user engagement…we know that the gap is the lack of social support in current mobile obesity interventions. Social support is vital for the persuasive systems design because: the more kinds of support in a system, the higher user engagement is. The higher user engagement is, the higher their compliance to the intervention. | Current mobile obesity interventions  have strong primary task and dialogue support. We also found that social support is much less  supported, despite persuasive design research that stated social support is vital in creating user  engagement. |
| Alnuaimi^13^  2019  Primary, Secondary Care  SR-QUAL  4 Studies | Individuals living with overweight and obesity (BMI ≥ 25 kg/m2) with and without comorbidities  (NR) Women: 45.56%-86% | Mobile-based interventions aimed at promoting weight loss. | Mobile application | HCP/ Doctor-Patient | patient–health care worker relationship, patient–doctor satisfaction, use of mobile apps and electronic device to sustain weight loss, time-saving merit of mobile-based applications | Mobile apps for weight loss management may negatively affect the patient–health care worker relationship among individuals living with overweight and obesity. | 3 key results: use of mobile apps is an effective tool in sustaining weight loss among intervention groups when compared to control groups. Mobile apps for weight loss management may negatively affect the patient–health care worker relationship. Mobile apps have been found to enhance patient satisfaction with weight management programs |
| Barnes^14^  2015  Primary Care  SR-QUAL  24 Studies | Adults in primary care (n=7448)  Men: 8-55% | MI for Weight Loss | Face-to-face (some mixed individual and group; 7/22 incorporated computer, internet or email technology) | HCPs/ Counsellors/ Community Health Educators/ Coaches/ Exercise Physiologist/ Trained Non-Specialists-Patients | Weight Loss | Treatment retention rates tended to be high for intervention conditions 80–89% and 90–100% ranges; the lowest MI intervention retention rates ranges were 50–69% | Potential for MI to help primary care adult patients lose weight and improve weight-related variables (e.g. BP) Conclusions, however, must be drawn cautiously as more than half of the reviewed studies showed no significant weight loss compared with UC/control groups and there was little existing evidence regarding MI treatment fidelity |
| Bennett^15^  2014  NR  SR-QUAL  6 studies | Racial/ethnic minority (at least 50%) adults ≥18 years old, living with overweight or obesity (BMI ≥ 25 kg/m2)  (n=4899) | eHealth adjunctive or sole interventions for weight loss, weight gain prevention or weight loss maintenance | Electronic health | Counsellors/ HCPs-patients | Weight change | Interpersonal contact is an important predictor of positive outcomes in eHealth trials.  With one exception, all studies reporting significant outcomes included additional support from a human counsellor.  Trials were methodologically sound, with high retention and participant engagement, potentially suggesting the appropriateness of eHealth interventions for the target population. Specific engagement metrics were quite variable or no engagement metrics reported. Rates of intervention engagement among racial/ethnic minorities similar to those in the broader eHealth weight loss Could not determine differences in engagement by race/ethnicity with the limited data available. | eHealth interventions can produce low magnitude, short-term weight loss among racial/ethnic minorities.  Some preliminary evidence as to the absolute weight loss expected with eHealth trials, we do not yet know whether eHealth approaches represent a non-inferior treatment option, relative to in-person individual or group-based intervention approaches for racial/ ethnic minority populations. There was no evidence detailing the efficacy of mobile health approaches, although this area is promising given high utilization rates of mobile devices among racial/ethnic minorities. |
| Burnet^16^  2006  Non-study settings SR-QUAL  4 studies | People living with overweight  (n=4748) | Lifestyle interventions to prevent or delay the onset of diabetes | Face-to-Face | HCP-Patient | Improve nutrition, lose weight, increase physical activity | These studies incorporated vigorous follow-up efforts, especially for subjects having less success. Subjects failing to meet initial goals were actively encouraged by staff. The DPP used computer monitoring to track program adherence and trigger actions for ‘‘recovery’’ of participants failing to reach goals. DPP staff used a stepped strategy to optimize outcomes, with a ‘‘toolbox’’ of problem-solving strategies valued at $100 annually per participant, including exercise tapes and classes, personal trainers, cookbooks, and other resources. | Diabetes prevention trials have shown dramatically how diabetes can be prevented or delayed through lifestyle changes, with remarkably consistent outcomes despite implementation across very different cultures. Clinicians should recommend behavior changes for asymptomatic patients at high risk for diabetes.  The rigorous cost-effectiveness analyses of the DPP provide a compelling case for increased insurance coverage of nutrition and physical activity interventions in persons at high risk for diabetes.  Relationships and social context are key factors for diabetes prevention. In these trials, close coaching relationships with study staff facilitated lifestyle change by participants. |
| Carraça^17^  2021  All settings SR-MA  63 Studies | Adults living with overweight (BMI ≥ 25 kg/m2) or obesity (BMI ≥ 30 kg/m2)  (n>12854) | Multi-component behaviour change interventions | Digital; Face-to-Face | HCP/ Commercial Provider-Patient/ User | Increasing PA (primary or secondary) | Regarding the main flaws, 26 presented dropout rates above 20%. | Effective BCTs for increasing PA in adults living with overweight and obesity in digital and face-to-face interventions seem to differ -using goal setting, social incentive, and graded tasks might be considered to improve PA outcomes in digital interventions. It might also be useful to opt for newer, more objective self-monitoring technologies to increase compliance. Face-to-face interventions - useful to prompt behavioral practice and rehearsal to obtain more favorable PA outcomes. There is also insufficient evidence to examine the effectiveness of most BCTs included in the two coding taxonomies; even more so if we consider long-term effectiveness, as less than half of the studies included a follow-up and of highly variable length. No consistent (theory-congruent) combinations of BCTs for increasing PA in this population were identified, calling for greater standardization at this level as well. |
|  |  |  |  |  |  |  |  |
| Cheatham^18^  2018  NR  SR-QUAL  25 Studies | Men and Women with a BMI >25 kg/m^2^) (n=2608) | Wearable activity tracker within a comprehensive WLP | Wearable Activity Tracker | NS | Weight loss | The attrition rate among all studies ranged from 2 to 32%. The higher attrition rates were found among studies with an intervention period of 6 months or greater. Unfortunately these studies did not report the reasons for attrition beyond the subjects drop-out rate | An activity tracker combined with a comprehensive WMP may provide superior short term (<= 6 months) results than a standard WLP in middle age or older adults. The comprehensive weight loss program may contain several components such as nutrition and behavioural counselling, diet and physical activity prescription, self-monitoring of diet and physical activity. |
| Chisholm^19^  2012  Medical School and placements  SR-QUAL  12 Studies | Medical School Students  (n=1362) | Educational interventions on obesity management | Didactic sessions; other educational methods included classroom-based learning, opportunities to practice learned skills, assessment, self-monitoring, and experiential learning | HCP-Medical Student | Studies with at least one outcome measure and sufficient (quantitative or qualitative) data to describe intervention effects. | The analysis firstly presents a description of participants’ current experiences of and perceptions about behavior change talk. Secondly three inter-linked sets of reasons why doctors do not engage in behavior change talk are presented. These are: (1) ‘personal challenges’; (2) ‘somebody else’s responsibility’ and (3) ‘prioritizing the doctor–patient relationship’. | Current educational interventions for medical students that address obesity management are varied, and empirical tests of their efficacy are inadequate. It therefore remains unknown to what extent medical students are being prepared to facilitate important lifestyle changes with future patients with obesity or what the best methods to achieve this involve |
| Chopra^20^  2020  NR  SR-QUAL  23 Studies | Patients living with overweight and obesity  (n=8187) | Behavioural lifestyle modification interventions | NR | Participant-Interventionist | Predictors of successful weight loss | Some of the intervention‐based factors associated with weight loss are initial weight loss, higher adherence and self‐monitoring. It can be postulated that early weight loss motivates the patient to adhere to advice, building confidence in the intervention and improves patient's engagement in the programme through greater session attendance, use of materials, maintenance of the diet and activity diary and other factors. Higher adherence and attendance was related to greater weight loss success; moderate and consistent level of adherence to behavioural weight loss programme leads to achievement of sustainable and realistic weight loss goals. Higher rate of attendance in the follow‐up sessions helps the interventionist to plan patient‐specific recommendations, educate self‐monitoring skills and manage challenges during the intervention, thus promoting better weight loss outcome.  Initial participant‐ interventionist bond was associated with significantly greater weight loss and treatment adherence at initial 4 weeks of the intervention. | Percentage weight loss success is related to duration and intensity of the intervention. The proportion of successful participants ranged from 14.7% to 67% in short and medium duration interventions and in case of long‐ term studies, about two‐fifth (43%) of participants achieved successful weight loss outcomes. demographics such as being male and of older age were graded as moderate quality predictor which were associated with greater chances of successful weight loss...Although being male is a non‐modifiable predictor, it can be concluded that it might be easier for the interventionist to keep a male patient motivated to adhere to lifestyle modification advice with limited follow‐up in comparison with female patient. On the other hand, it can also be inferred that more interventionist support should be extended to a female patient trying to achieve successful weight loss. |
| Cooper^21^  2018  Community, outpatient  SR-MIXED  17 Studies (Trials n=14;  Qualitative Studies n=3) | Adults (≥18 years) with overweight/obesity (BMI  ≥25 < 30 kg/m2 overweight; BMI ≥30 kg/m2 obese) and chronic musculoskeletal pain (n=4607) | Any weight management interventions including bariatric drugs surgery  and lifestyle modifications such as diet, physical activity  or psychological interventions delivered as part of a multi  or single component study | Face-to-Face | HCP-Patient | Primary outcome: change in weight.  Secondary outcome: change in pain,  Perceptions and experiences of engaging with weight loss interventions and sustaining weight loss efforts long term. | Meta-analysis and narrative analysis in this review demonstrated that adults with overweight/ obesity and CMP can engage with structured weight loss interventions including exercise. | Low-quality evidence from the quantitative component and moderate quality evidence from the qualitative component suggests HCP and policy makers should emphasize the benefits of managing weight and CMP simultaneously. Interventions designed to combine management of weight and specific CMP conditions could maximize weight loss and reduce pain. |
| Coupe^22^  2019  All settings  SR-QUANT  10 Studies | Adults ≥18, with a BMI ≥25 or if amount overweight or weight loss goal ≥15lbs or percentage overweight ≥10%  (n=1214) | Soft  commitment device accompanying a behavioural lifestyle  intervention targeting diet and/or physical activity | Face-to-face (1 individual had web support) | HCP-Patient | Primary outcome: change in weight (kg) or BMI.  Secondary outcome: were  behaviour change | Interventions that included a course fee or deposit not relating to the contract goals (e.g. for attendance) were included as they were not contingent on the contract behaviour or outcomes, and therefore were not identified as an incentive.  Five of the included 10 studies compared variations of behavioural contracts, and three of these did this exclusively without a non-contract control. Four studies focused on the involvement of others such as peers or healthcare professionals, either through publicity, attendance, or co-signing of contracts. Nyer and Dellande compared two levels of public commitment groups which are discussed here. The fifth study included two behavioural contract groups in which we are interested; one alongside a group intervention and one alongside an educational booklet with minimal contact. | Findings suggest that  soft commitment devices, specifically non-financial behavioural contracts, have potential for facilitating short-term, and possibly longer term weight loss and dietary  behaviour change when used alongside a lifestyle intervention. Including a public element to the contract, specifically where witnessed by a provider or peers, appear to improve outcomes. |
|  |  |  |  |  |  |  |  |
| Flodgren^23^  2017  SR-QUAL  Primary Care  12 Studies | HCPs; Adults living with overweight or obesity (Children living with obesity)  Practices (n=139); Patients (n=89754) | Interventions to change the behaviour of HCPs or organisations to promote weight loss. | Face-to-face | HCP-Patient | Body Weight, BMI | NR | We found little convincing evidence for a clinically-important effect on participants' weight or BMI of any of the evaluated interventions. While pooled results from three studies indicate that educational interventions targeting healthcare professionals may lead to a slight weight reduction in adults, the certainty of these results is low. Two trials evaluating CDS tools (unpooled results) for improved weight management suggest little or no effect on weight or BMI change in adults or children with overweight or obesity. Evidence for all the other interventions evaluated came mostly from single studies. The certainty of the included evidence varied from moderate to very low for the main outcomes (weight and BMI). All of the evaluated interventions would need further investigation to ascertain their strengths and limitations as effective strategies to change the behaviour of healthcare professionals or the organisation of care. As only two studies reported on cost, we know little about cost effectiveness across the evaluated interventions |
|  |  |  |  |  |  |  |  |
| Gostoli^24^  2024  Mixed settings: clinical and outpatient clinics  SR-QUANT  31 studies | Adults (aged 18 years+) living with type 2 diabetes and overweight or obesity (n=7272) | BWMI including device-based, and educational interventions | Face-to-face; remote and hybrid | HCP-Patient | Weight loss | NR | Although in the literature it emerges the importance of lifestyle interventions in populations living with overweight/obese and diabetes, in this systematic review it was found that not all proposed and analyzed interventions are effective for weight loss. In fact, it is not enough to engage behavioral intervention for the management of this disease, but, as the findings of the present study reveal, it is necessary that the behavioral intervention includes specific components to achieve a clinically significant weight loss and, as a result, a general improvement. Findings show that the most effective behavioral interventions for achieving significant weight loss are those that include accurate instructions regarding dietary behavior, stimulate behavioral change through regular meetings (weekly or biweekly), and provide feedback and capillary support to patient’s path through individual or group sessions. This systematic review shows that physical activity alone, in contrast to diet, does not produce the best results. Specifically, diet is more effective when associated with educational ingredients, with the most effective ones in achieving weight loss through high protein, very low calories (400–500 kcal), low calories (1000–1500 kcal), fat and carbohydrates and ketogenic diets. In contrast, physical activity produces better results when integrated within a global lifestyle intervention, in comparison with exercise alone. Given the limitations of this work, further investigations including the elements that emerged from this review are needed, in order to systematize effective protocols increasingly widespread and focus on specific targets such as, in this case, patients with T2DM. Moreover, compared to previous works, this systematic review used a more rigorous methodology to assess the efficacy of behavioral lifestyle |
| Gudzune^25^  2015  NR  SR-MA  39 Studies | Individuals living with overweight and obesity  (n=5637) | Commercial and proprietary weight loss programs | Face-to-face; telephone | Counsellor/ PCP/ Psychologist-Patient/ Participant | Weight loss benefits, adherence, and harms | Many trials had high attrition. Adherence was reported variably. In many trials, study staff assisted in program retention and trials often covered the costs of these programs for participants. Therefore, the study results are likely better than can be expected in a “real world” setting, as a prior study of one commercial program reported retention of only 7% at 12 months. Studies often failed to report adherence/engagement or adverse outcomes. When described, program adherence was reported differently across trials,  making comparability across studies challenging. | Consistent evidence supports the long-term efficacy of Weight Watchers and Jenny Craig, while NutriSystem may require 12-month or 24-month RCTs reporting ITT analyses before we can be confident of the long-term effect. VLCD approaches can achieve substantial short-term weight losses; however, enthusiasm is limited due to potential risks and the lack of evidence supporting sustained long-term weight losses. |
|  |  |  |  |  |  |  |  |
| Harper^26^  2018  University, clinical and commercial settings  SR-QUAL  3 Studies | Men and women who had undertaken a weight loss regimen consisting of a total meal  replacement diet with a maximum energy intake prescription of 3,350 kJ d-1 (800 kcal d-1) (i.e. VLEDs)  (n=47) | VLED for weight loss | Face-to-face (included telephone and email) | NS | Participant experiences | Those participants who undertook a VLED as part of a clinical trial experienced an added sense of responsibility towards adherence, as well as camaraderie with research staff, both of which highlighted an important difference between a research-based intervention and a commercial weight loss programme (23,25).  This sense of accountability can be replicated outside of a research facility and that primary care may be a feasible setting in which to deliver VLEDs as a weight loss treatment. | With targeted and effective support, participants found VLEDs were easily adhered to and that they triggered changes in lifestyle to support the positive outcomes experienced during the VLED. It is essential that healthcare professionals have an in-depth understanding of how their clients may experience a VLED while losing weight and what strategies have been successfully employed by others, and this review can give healthcare professionals insights into using VLEDs as a dietary treatment option for overweight and obesity. |
| Hartmann-Boyce^27^  2015  SR-MA  23 Studies (43 Reports) | Adults (aged ≥18  years) with a BMI of  ≥ to 25 kg/m2  (or a BMI of ≥23 kg/m2 in Asian  populations)  (n=9672) | Self-help BWMI (not assisted by HCP) | Face-to-face; Remote | Researcher-Participant | Weight Change | At 8 weeks, mean weight loss in the contract and voicemail arms was less than that in the mail-only arm, but because of high levels of dropout, longer-term effects were not reported. | Results from this review show promising evidence of the effectiveness of self-help interventions for weight loss. Public health practitioners and policymakers should look to implement self-help interventions as a component of obesity intervention strategies because of the high reach and potentially low cost of these programs. Interactive and tailored programs may be more effective than reading materials alone. Further progress in understanding and improving the effectiveness of self-help interventions will come from using the taxonomy described here, which focuses on the strategies individuals might use themselves. Future trials might also test the relative effectiveness of interventions by SES and build longer-term follow-up into their plans. Notwithstanding these caveats, the evidence is now clear that self-help interventions can be effective and that such interventions may have an important role to play to reduce the population prevalence of obesity |
| Johnson^28^  2013  UK settings SR-QUAL  17 Studies | Women who were pregnant or had been pregnant; health professionals involved in caring for women that were pregnant.  (n≈434) | Weight management interventions | NR | HCPs-Women during pregnancy | Views and experiences of women and HCPs about dietary and/or physical activity interventions aimed at managing weight during pregnancy and views about  carrying out these activities outside the intervention setting | Women were reported to feel blamed for their weight following some interactions with health-care professionals. Complaints and reports of perceived victimisation regarding their size were reported to have resulted in women moving their care to other units (Heslehurst et al., 2007). Professionals were therefore concerned that women may miss out on crucial care if they felt too embarrassed to attend. The service name ‘maternity obesity service’ was highlighted as a potential source of embarrassment for women that affected their rapport with professionals (Heslehurst et al., 2011). | Targeting weight management advice at women during pregnancy may reduce the risk of complications and increase wellbeing for the mother as well as the unborn infant. However, exploration of women’s and health professional’s views within the UK suggests that women require sensitively delivered advice that accounts for their personal attitude towards their weight and its management. For this to be achieved midwives require specialised knowledge about nutrition and physical activity during pregnancy, and/or appropriate referral information. Evidence also suggests that poor communication skills by some health professionals act as a barrier for uptake of advice. Pregnancy may not be the optimum time to promote weight management due to a range of barriers that women face at this time, but it could be addressed as an aim for the postpartum period, and ideally before a pregnancy is planned. |
|  |  |  |  |  |  |  |  |
| Khaylis^29^  2010  NR  SR-QUAL  21 Studies | Participants  (n=2850) | Technology-based intervention for weight loss | Internet, telephone, Text | Health Coach/ Counsellor-Participants | Weight reduction | Interventions that were individually tailored to participant goals had higher rates of adherence and weight loss | Technology has provided health professionals with an opportunity to improve behavior change models by making them more convenient, accessible, and continuous. Technology that incorporates the 5 components is likely to facilitate behavior change that has substantial impact on public health. As rates of obesity and associated health problems continue to rise in the United States, the interface of technology and behavior change is an effective foundation of a successful, short-term weight-loss program and may prove to be the basis of long-term weight loss. |
| Makin^30^  2021  NR  SR-MA  12 Studies | Adults (18 years and above) with BMI ≥ 30 kg/m2  (n=6249) | MI | Face-to-face (some telephone or online) | Practitioner-Participant | Primary outcome:  measures of adiposity, (e.g. BMI, kg).  Secondary outcomes: measures of motivation or  adherence to other treatments, such as  BWLP | Around a third of papers were considered potentially at high risk of attrition bias due to lack of reporting about participant drop-out or handling of incomplete data (37.9%). | this review identified that a range of methodologies of MI have been researched for effectiveness of weight loss outcomes, and a meta-analysis found no significant overall effect. However, no significant increases in weight comparable to the comparator groups were observed. Frequently reported BCTs included social support, self-monitoring of behaviour, problem solving, goal setting and information about health consequences, which is consistent with current recommendations from government bodies such as PHE and demonstrates integration of MI with these techniques. |
| Michalopoulou^31^  2022  Range of settings  SR-MA  46 Studies | Adolescents (aged ≥12 years) or adults  (n= 11077) | MI as part of BWMP | Face-to-face (some text, email, telephone components) | HCP-Patient | Weight change | A quarter of studies were high RoB, primarily due to attrition.  Ten of 15 studies in which the 2 groups received interventions of similar intensity reported program attendance or engagement data. Seven of 10 studies reported that participants allocated to MI had better attendance or engagement. | We found no evidence of benefit from MI on mental health or well-being outcomes. However, MI was used not as a standalone intervention but rather in addition to components typical in BWMPs, such as goal setting, monitoring, and provision of feedback. These elements may be at odds with the spirit of MI and could have undermined the potential benefit on mental health outcomes. In summary, BWMPs that incorporate MI may slightly increase weight loss in the short term compared with minimal or no intervention, but there is no evidence that MI increases weight loss compared with otherwise similar BWMPs without MI. There is little evidence to recommend MI as a specific component of BWMPs. |
| Manzoni^32^  2011  Various settings  SR-QUAL  26 Studies | Adults living with overweight  and/or obesity  (n= 8324) | Internet-based behavioural interventions | Internet at least  one mode of delivery for a weight loss treatment (some face-to-face, telephone, email) | NR | Weight loss or weight maintenance | Attrition rates varied from 0 to 70% at the post-intervention time point. Two studies lost greater than 50% of participants, eleven lost 20–50% and twelve 0–20% with a mean attrition rate of 22,52%. | The Internet offers a novel delivery tool for weight loss  and weight loss maintenance interventions, with the potential to offer long-term intervention at a low cost, in comparison  to traditional face-to-face treatments. |
| Marques^33^  2021  Primary Care  SR-QUAL  56 Studies | Adults (age 20-59 years) or older people (≥60 years) living with obesity  (n=21696) | Overweight or obesity treatment interventions | Face-to-face, e-health (e.g. text, phone, apps) | PHCP-Patients; Researchers-Participants | BMI, body weight  (BW) and waist circumference (WC) | NR | Our study showed that most interventions were effective for outcomes analysed in adults and the elderly. We also found some literature gaps, such as the need to implement and evaluate obesity after intervention and the requirement to carry out more studies in low- and middle-income countries. |
| McDonald^34^  2022  Various Settings  SR-QUAL  36 Studies | Men with a mean  baseline participant BMI of ≥30 kg/m2 and/or a  participant eligibility criterion BMI of ≥25 kg/m2  (n=5017) | Interventions targeting weight, diet, and/or physical activity with a behavioral component, such as education,  BCTs, psychological theories, or counselling, were included. Interventions could include group-based or  individual, face-to-face, or e-health interventions | Face-to-face, Remote | NR | Weight or BMI | Due to the heterogeneity of socioeconomic measures across studies and countries, moderation analysis of socioeconomic status on weight outcomes and retention rates was not undertaken.  Gender-tailoring and aligning interventions with men's interests can engage men who would not otherwise participate; however, depending on the context, specific targeting (e.g., in lower socioeconomic areas or regional towns) and tailoring (e.g. for specific ethnic groups) may be required to reach more diverse populations more representative of the burden of obesity-related disease  There is currently limited evidence on the reach and effectiveness of interventions across the socioeconomic spectrum, or for interventions designed specifically for men from lower socioeconomic circumstances. However, some recent feasibility studies did aim to engage specific socioeconomic and ethnic groups. | Trials of interventions with a behavioural component for men that report weight as an outcome, socioeconomic factors are inconsistently reported, men from lower socioeconomic circumstances are rarely targeted or consulted during intervention design, and there is scant evidence that interventions reach or are effective in lower socioeconomic groups. |
| Morrow^35^  2022  Workplace SR-QUAL  9 Studies | People living with overweight or obesity;  HCPs  NR | Workplace-based physical activity and/or dietary interventions | Face-to-face, 2 studies online platforms  1 study individual only remaining group or mix of group and individual | HCP-patient | Weight loss | Retention rates varied, with six of studies achieving over 80% retention rate. | The review fails to highlight any observable relationship  between the mode of intervention delivery and significant weight loss.  Evidence suggests that a combined online and face-to-face approach is most successful at supporting adults living with overweight and obesity with weight loss This review does not allow the opportunity to effectively compare  mode of delivery due to the diverse nature of interventions. |
| Pearson^36^  2012  Community  SR-QUANT  18 Studies | Adults living with overweight and/ or obesity  (n=1187) | Goal setting as the primary intervention/ principal  component effecting dietary and/or physical activity  behavior change | Face-to-face, Most studies included telephone, or technological delivery via e-mail or through a computer program) | NS | Behavioural outcomes | When comparisons were made to distal or no goal groups, findings revealed some favourable outcomes for proximal goal setting as evidenced by changes in eating behaviours and weight loss. Alternatively, Zegman and Baker found greater reductions in caloric consumption in the distal goal setting group during treatment; however, attrition rates in this same group were also higher compared with the proximal group. | Positive behavioural and physiological results were observed across the selected studies including decreases in weight, BMI levels, and food and beverage consumption, as well as increased energy expenditure, and greater intentions to perform healthy behaviours. As several different components were implemented concurrently (e.g., education sessions, personalized feedback, self-monitoring devices), over various lengths of time, and not assessed specifically to determine effectiveness on outcomes, it is not possible to ascertain which were responsible for the positive behavioural and physiological changes observed. Thus, causal inferences and recommendations pertaining to the optimal combination of goal setting components cannot be made with certainty. |
| Perperidi^37^  2023  Mixed Setting: Clinical, homebased and remote.  SR-QUANT  11 studies | Female adult breast cancer survivors with BMI ≥ 25 kg/m2 and without any active  cancer therapy or any ongoing treatment, except of hormonal  or immune therapy | Dietary and physical activity components targeting  overweight/ obesity with the use of at least one behaviour  change theory or model | Face-to-face, telephone, digital/remote platforms and written material | HCP-Patient;  Researcher-Participant | Weight loss | NR | Considering the unique needs of breast cancer survivors, lifestyle interventions should include behavior modifications in diet, physical activity and psychosocial factors according to the official guidelines with the use of behavioural theory and the suitable behavioural change techniques. The completion of the initial cancer treatment signals a critical period, in which breast cancer survivors may adopt healthy behaviours thus maintain or adopt a healthy body weight. The identification of the optimal methods targeting obesity seems vital for lowering the risk of cancer recurrence and overall mortality. The taxonomy can help with the understanding of behaviour change tools used in the interventions; thus, the need for stronger identification of the components of the intervention using a taxonomy of behavioural change techniques should be highlighted. The findings of this systematic review may enable healthcare professionals to include the reported elements and BCTs in their behavioural interventions in order to help breast cancer survivors to improve their lifestyle, control their body weight and maintain the changes more effectively |
| Pritchard^38^  2022  NR  SR-QUANT  7 Studies | Long-distance (≥ 500 kms per day) truck drivers who were 18 years old  (n=1214) | Weight loss interventions included physical activity, diet therapy,  behavioral therapy, and health promotion or education programs | Face-to-face, online, phone | Clinicians/ Coaches-Patients | Body weight or some measure related to body composition related to weight loss (e.g. BMI, WHR), skin-fold  thickness), or impact on LTHC associated with obesity | There were moderate to high levels of attrition (23–49%) at follow-up | A combination of MI and supporting resources has potential for long-term effectiveness in reducing truck driver body weight. However, the level of evidence in this area is minimal with only two RCTs available. Findings presented are also inconclusive due to the level of bias, small sample sizes, and designs of each of the studies included in this review. |
| Robertson^39^  2014  Societies relevant to the UK setting  SR-MIXED  95 Studies  Total: 95  (33 RCTs with  12 linked reports, 24 non-randomised  reports, five economic evaluations with two linked reports, and 22 qualitative studies) | Men living with obesity BMI ≥ 30 kg/m2 or overweight men with a BMI ≥ 28 kg/m2 with cardiac risk factors.  (n=89500 men and women) | Interventions explicitly promoting weight loss or weight maintenance as their main outcome. We considered lifestyle changes (e.g. diet, physical activity, BCTs or combinations of any of these) and orlistat | Face-to-face, telephone, online | HCP/ Researcher-Patient/ Participant | Primary outcomes: Weight, WC, cardiovascular risk factors, disease-specific outcomes, adverse events, quality of life, process outcomes and economic costs  Barriers/ facilitators of men’s experiences in weight management. | No eligible studies for review of interventions to increase the engagement of men.  Eight trials provided data that could be included in the analysis comparing the numbers of men and women who completed the trials. In total, there were 3813 participants; 1197 were men and 2616 were women. The results show that men were 11% (95% CI 8% to 14%, p < 0.001) more likely to complete the trial than women, suggesting men are highly motivated once commencing weight loss.  Attrition rates for programmes designed for men tended to be low but programmes were short and numbers of men recruited were low, with the exception of the LighterLife study148 (although the attrition rate for this study is unknown). | Weight reduction for men is best achieved and maintained with the combination of a reducing diet, physical activity advice or a physical activity programme, and behaviour change techniques. Tailoring interventions and settings for men may enhance effectiveness, though further research is needed to better understand the influence of context and content |
| Rose^40^  2013  Primary Care SR-MA  32 Studies (MA n=12) | Patients and PCPs (e.g. physician, nurse practitioner or  physician assistant  (n=466645) | PCP weight  loss advice | Face-to-face | PCP-Patient | Weight loss behaviours and weight loss efforts | 22 studies looked for an association between weight loss advice and patient engagement in weight loss efforts. Of the 22 studies, 11 used data from the BRFSS, 4 used NHANES data, 1 used NHIS data and 6 used local study data. Only one did not find a positive association | We found that provider weight loss advice positively affects  patient weight loss behavior in all outcomes addressed.  PCPs need to recognize their influence when it comes to advising their patients about their weight. |
| Sherrington^41^  2016  NR  SR-MA  12 Studies | Adults (18+ years) with BMI > 25 kg/m2  (n=3547) | Intervention targeting diet and/ or physical activity for weight loss, incorporating any form of individualized feedback  either human-delivered  or  computer-generated personalized feedback  delivered via  web-based messages or email | Personalised feedback via internet-delivered intervention | HCP/Researcher-Patient/ Participant | Primary: Body weight change  Secondary: body fat, WC or BMI change,  retention rates | Attrition rates in this review ranged  from 12 to 47%, therefore are similar to previous findings.  Retention rates were calculated by the number of partici-pants who provided follow-up data at the last assessment point (varying between studies). In total, intervention groups retained 73.5% (1132/1540) of participants, whilst control groups retained 77.5% (1036/1336), a signiﬁcant difference in retention rates between intervention and control groups (p < 0.05). | Incorporating personalised feedback may be an important BCT for effective weight loss interventions delivered via the internet. Participants within the IWLPF were identiﬁed as twice more likely to achieve 5% weight loss than those in control groups. Shorter term data collection, 3 or 6 months, produced signiﬁcant differences between the IWLPF and the control groups receiving no personalized feedback for all outcomes. In contrast, interventions lasting 12 months or longer did not produce signiﬁcant differences between IWLPF and control groups receiving no personalized feedback for weight loss. Subgroup analysis identiﬁed signiﬁcantly greater weight loss for the IWLPF irrespective of the comparator used, whether wait list/minimal face-to-face interventions or control internet-delivered interventions receiving no personalized feedback |
| Sherson^42^  2014  Primary Care SR-QUAL  15 Studies | PCPs;  Patients living with Overweight or obesity  (n=3416) | 5 A's (Assess, Advise, Agree, Assist and Arrange) Weight loss Counselling | Face-to-face | PCP-Patient | Patient perspective, viewpoint, opinion, and belief, or physician use and physician practice patterns | Adherence - Evans (32) surveyed individuals living with obesity who had lost ≥ 45kg while attending a commercial weight loss group about physician involvement. Two of the studies surveyed participants in their PCPs waiting room. The trends were similar to the studies reported above, with ‘Assess’ and ‘Advise’ being commonly used, and ‘Agree, Assist and Arrange’ being rarely used. Additionally, 33% of patients living with obesity reported that their physician did not address weight with them in the study by Potter et al. | Patients would like their physicians to ‘Arrange’, followed by ‘Advise’, ‘Agree’ and ‘Assist’. There was no data regarding how often patients want to be ‘Assessed’. In comparison, physicians most frequently ‘Advised’ and ‘Assessed’, but rarely ‘Agreed’, ‘Assisted’ or ‘Arranged’. This disconnect identifies a significant gap between patient needs and physician practices that likely affects patient care. When a patient living with overweight or obesity is ready to talk about their weight and receive advice from their physician, it seems likely the patient may have a greater chance of success at achieving weight loss if the physician is able to systematically implement the full 5 A’s model with the patient over time |
|  |  |  |  |  |  |  |  |
| Tang^43^  2016  Community, Workplace, University  SR-MA  27 Studies | Healthy adults (≥18 years) including those at risk of diabetes who were not taking medications which could affect weight;  people of normal weight or living with overweight or obesity  (n=28500) | Self-directed Interventions for weight loss | Face-to-face; internet, text messages | HCP-Patient | Body weight (kg or lbs), BMI kg/m2, WC (inches or cm) | With only one initial face-to-face professional contact, they can prolong client and professional contact engagement without further professional contact. Only one of the trials considered here included an economic evaluation and they found that a self-directed internet-based intervention cost £716.28 more per person per year compared to usual care. However, they advised caution in the interpretation of results because of high attrition and low compliance. | Self-directed WLI that do not involve ongoing professional support, including those delivered using the internet, can generate modest significant weight loss at up to six months but, as yet, there is little evidence to suggest that they promote weight-loss maintenance. Since they are likely to be cost-effective, when effective, they may provide useful adjuncts to existing community and clinical services, and could potentially be used exclusively to generate clinically meaningful weight loss. |
| Tapsell^44^  2016  SR-QUAL  16 Studies | Adults  NR | Interdisciplinary interventions (defined as  those involving more than one type of HCP) | Face-to-face (included individual and/ or group) | HCP-Patient | Anthropometric, BP, or biochemical outcomes | Greater effects were seen where there was a greater amount of contact between HCPs and participants. Only two RCTs reported a follow-up period. Sustained and superior effects were seen where there was retained (and more frequent) contact with health professionals. No difference in long-term effects was seen where there was no contact between the end of the intensive phase and the follow-up period. | Interdisciplinary lifestyle interventions, involving AHPs, and in some cases, primary health care professionals, can result in significant improvements in chronic disease risk factors, but intensity of intervention needs to be considered. |
|  |  |  |  |  |  |  |  |
| Walsh^45^  2011  Any  SR-QUAL  5 Studies | Primary health care professionals and patients living with overweight or obesity  (n≈935, 2 NR) | Weight loss counselling | NS | PHCP-Patient | Interaction between PHCP and patients - ‘Interaction’ defined as a communication where there is a two‑way sending and receiving of verbal and non‑verbal information | Attendance | Several factors inhibit effective management of overweight and obesity by PHCP, and impact on their interaction with patients. Key factors include PHCP practice skills and knowledge, lack of resources, and inconsistent overweight and obesity management guidelines. The emotive and personal nature of overweight and obesity, and the attitudes and beliefs of PHCP also impact on the interaction. Education of PHCP needs to address their attitudes and beliefs about overweight and obesity. Equally, education in the diagnosis of overweight and obesity and the acquisition of effective weight loss counselling skills is also important in improving PHCP‑patient interactions. |
| Wieland^46^  2012  Community SR-MA  18 Studies | Adults living with overweight or obesity  (n=4140) | Interactive computer intervention for weight loss or maintenance | Computer-based, internet, face-to-face | Provider-Patient | Primary outcomes: Body weight (kilograms or pounds or BMI), WC, HRQOL, wellbeing and patient satisfaction.  Secondary outcomes: physical activity or dietary-related outcomes, cost-effectiveness, adverse events | Greater amounts of intervention use, particularly self-monitoring, were typically associated with greater amounts of weight loss, though it was not clear what intervention elements would best encourage greater use or whether encouraging greater use would lead to larger effects.  Assessed attrition rates, noted whether reasons for dropout were given, and whether methods of handling missing data were stated – not summarised. | A variety of interactive computer-based interventions led to short-term weight loss when compared to a minimal or no treatment condition. Computer-based feedback also appeared to be effective as an adjunct to Internet interventions in promoting weight loss. Fewer trials tested computer-based interventions for maintenance of weight loss, however computer-based interventions consistently led to lower levels of weight regain when compared to a minimal or no treatment condition. Few interventions led to changes in caloric intake, physical activity or WC, though these outcomes were not reported in all studies.  While interactive computer-based interventions appear to be more effective than a minimal or no treatment control, they do not appear to be more effective than in-person treatments, even in cases where the in-person contacts are infrequent. |
| Young^47^  2011  NR  SR-MA  23 Studies | Males aged 18–65 years living with overweight or obesity  (n=1869) | Weight loss or weight loss  maintenance interventions with clear intent to change  behaviour or lifestyle | Face-to-face (individual and group; some email contact) | NS | Weight change  or weight at baseline and a minimum of one post-intervention time point (KG or pounds) | Weight Loss: Fourteen studies (61%) met the criteria for adequate retention rates (dropout ≤20% for ≤6-month follow-up and ≤30% for >6-month follow-up).  Weight maintenance: . Three studies included sufficient follow-up but only two reported adequate retention rates.  Preliminary evidence suggests men-only weight loss programmes may effectively engage and assist men with weight loss. | Several characteristics of interventions may be linked to effectiveness in male-specific  weight loss studies: (i) a prescribed energy  restriction; (ii) inclusion of group face-to-face contact; (iii) higher frequency of contact (>2.7 contacts/month) and (iv) a younger sample (mean age 42.8 years). |

| Characteristics of Red Reviews | | | | | | | |
| --- | --- | --- | --- | --- | --- | --- | --- |
| Author(s), Year,  Setting,  Synthesis Type,  No. Studies | Population/ Participant Description (n) | BWMI Description | Mode of delivery | Relationship Described | Perspective and/or Outcome | Retention (e.g. attrition, engagement) | Conclusion |
| Abbott^48^  2020  UK  SR-MA  7 Studies | Adults with a BMI ≥ 25 kg m^2^ (n=2576) | Multicomponent lifestyle interventions for weight loss | Face-to-face  (Exclusively groups compared to exclusively 1-to-1) | HCP/ Weight Loss Coach-Patient | Weight Change | Individuals attending a group intervention were 58% more likely to attain a 5% weight loss at 12 months relative to attending one-to-one interventions (relative risk=1.58, 95% CI=1.25–2.00, P=0.04; I2= 60%). Sensitivity analysis including only ‘low’ risk of bias studies did not alter the findings (relative risk=1.51, 95% CI=1.14–2.00, P=0.03; I2=66%).  If there is the option for an individual seeking weight management to attend either a group or a one-to-one intervention, the findings of this review suggest that attending a group over a one-to-one intervention will lead to greater weight loss at 12 months. However, patients’ choices should be exercised to promote treatment fidelity. | Multicomponent lifestyle interventions delivered in groups are more effective for weight loss compared to one-to-one interventions for adults with a BMI ≥25 kg m^2^ |
| Aguilar-Martinez^49^  2014  NR  SR-QUAL  10 Studies | Adults living with obesity or overweight aged 18+  (n=1156) | Tools or apps for mobile phones, whose primary aim was to achieve weight loss or weight loss maintenance | Mobile phones and associated apps | Researcher-participant (weight assessment) | Weight Change | Social and personal circumstances (mainly work-related) had the greatest effect on participant drop-out | Diversity and limitations in the studies reviewed. Elements that were important to the successful use of apps in obesity: proportional relationship between weight loss and programme use; programmes most benefited those who took a pro-active approach to everyday problems; frequent self-recording; personalisation of the intervention (counselling & individualized feedback); a social support system acted as a motivational tool. |
| Alamnia^50^  2022  Developing Countries. Institutions, Community  SR-MA  12 Studies | Adults aged 18 years and above living in developing countries  (n=4059) | Text message delivered interventions for weight loss | Mobile - SMS Messages (some telephone and face-to-face) | HCP-Patient | Change in body weight (Kg/BMI) | Attrition | This systematic review and meta-analysis showed that text message delivered interventions for weight loss among adults in developing countries are apparently effective given the limited evidence to reach a rough conclusion. From 12 trials, 10 reported significant effect of text message interventions for weight loss. Frequent and extended interventions and use of behavioural theories resulted in a more significant weight reduction in adults with overweight and obesity. |
| Armstrong  2011  NR  SR-MA  12 Studies | Adults living with overweight or obesity (BMI >=25.0 kg m^2^)  (n=3636) | Behaviour change using MI | Face-to-face, telephone (some group) | HCP/ Health Counsellors/ Exercise Scientists-Patients | Body mass (kg or BMI) | MI appeared to improve adherence to the BWLP. The degree of adherence to weight-loss interventions can be a strong predictor of weight loss. Smith et al. (36) observed significantly increased attendance by MI participants at BWLP sessions than controls (P < 0.01). In a study investigating the effect of WMP adherence on weight loss, Finley et al. (50) demonstrated that improved programme adherence enhanced absolute weight loss over 1 year. Given these improvements in retention, it seems possible that MI may work to increase attendance to a behavioural WLP resulting in greater weight loss. However, in this systematic review there were a limited number of available studies thereby limiting the generalizability of this conclusion. It may be important to consider the base intervention to which MI is applied. | MI appears to be a promising value add for weight-loss interventions in obesity management. It is consistent with recommendations from patients, healthcare providers and researchers for more ‘patient-centred’ approaches in health care, where the provider–patient relationship is seen as a partnership, rather than an expert–recipient one. It provides practitioners with a means of working with patients who are ambivalent about change. This meta-analysis suggests that MI is a useful intervention in weight management and its effectiveness may be enhanced when applied alongside BWMPs. |
| Balk^51^  2015  Clinic, community  SR-MA  53 Studies | Adults or children at increased risk for T2DM (i.e., with pre-diabetes) (including participants with metabolic syndrome or whose participants were chosen because they were at risk for either T2DM or CVD)  NR | Combined Diet and Physical Activity Promotion Programs | At least 2 contact sessions (in-person or virtually) over a minimum period of 3 months. 7 programs were delivered entirely over the Internet or by video, telephone, or e-mail, programs used in-person individual or group sessions (or both) | HCP/ trained laypersons-patients | Incident diabetes, reversion to normoglycemia, body weight, glycemia measures all cause death, diabetes-related clinical | This review did not specifically address participant attrition, a better understanding of typical attrition rates is needed to understand the reasons program participants drop out and to develop methods to retain them. | Combined diet and physical activity promotion programs are effective in reducing new onset diabetes, increasing reversion to normoglycemia, and improving diabetes and cardiometabolic risk factors in persons at increased risk for type 2 diabetes. Programs are effective across a wide range of features, but more intensive interventions seem to be more effective. |
| Bassi^52^  2014  Primary Care  SR-QUAL  28 Studies | Patients with one or more components of metabolic syndrome  (n=6212) | Tailored lifestyle-change regimen with peer support and modern technology | Face-to-face, telephone (some group) | PCP-Patient | Real-time adherence monitoring strategies included tools such as questionnaires dietary recall surveys and exercise diaries | 2 high-quality studies included in our review show that adherence, not the specific dietary prescription, contributes most to successful weight loss programs. Patient motivation was found to be critical for improved adherence. This should be a key element of any successful strategy.  Three replicable strategies can be implemented in the primary-care setting. Interactive programs, such as group sessions, engage patients much more effectively than passive strategies. Adherence improves when patients are held personally accountable with frequent personal feedback. A team-based approach, with dieticians trained in motivational interviewing, can improve adherence.  If adherence or resources are an issue, prioritizing a diet-based program may be more effort- and cost-efficient. Allowing patients to choose their own calorie-controlled diets may help increase acceptability and adherence. | A successful lifestyle intervention for the metabolic syndrome, paradoxically, does not depend much on the exact prescription of diet and exercise, but rather, on the patient’s motivation to change, as reflected in improved adherence. A focus solely on the specifics of the lifestyle regimen may not be sufficient. We found evidence that expanding the focus to include methods of increasing patient participation and motivation through specifics of delivery (use of technology and promotion of personalized feedback and individualized goals) as well as setting (group-based strategies; team-based approach) can enhance the realization of a cost-efficient and effective program. Although technology is an exciting advance, our review could not endorse technology alone for the needed improvement in lifestyle to treat the metabolic syndrome. We conclude that technology enhances, rather than replaces, personal contact for continued successful control of metabolic syndrome risk factors. |
| Beleigoli^53^  2019  Community, University  SR-MA  11 Studies | Adults (≥18 years) living with overweight and obesity (BMI ≥25 kg/m2)  (n=1525) | Web-based digital intervention (accessed by browser or Web-based application, regardless of device) | Digital (internet, smartphone app) | NS | Weight and BMI change | Intervention-induced weight loss was of small clinical significance. This happened even in studies with a short-term follow-up. Low engagement to the interventions delivered by the Web-based tools might explain these modest results and might be a proxy for the low motivation of participants [23]. These modest results also highlight the need to investigate the components and tools of Web-based platforms that lead to the maintenance of users’ motivation, interest, and participation, which play a key role in enhancing adherence to healthy behaviours.  The high dropout rates in the retrieved studies contributed to a lowered quality of evidence and suggest that designing interventions that maintain participants’ engagement and motivation over time might be fundamental to the success of digital interventions. | There is moderated certainty in our findings that Web-based digital health interventions are more effective than non-technology interventions in promoting short-term but not long-term weight loss. Moreover, Web-based interventions do not seem superior to non-technology ones in terms of changes in dietary and physical activities. |
| Blue^54^  2005  Naturalistic settings  SR-QUAL  30 Studies | Healthy Adults (18 years or older)  (n≈11622, 1 NR) | Activity-related interventions of supervised or unsupervised | Face-to-face | Behavioural outcomes and indicators or behaviour change | Targeted behavioural outcome (e.g. physical activity, dietary change, physiologic changes) | An association was reported between attendance during the first 6 months and the intervention and greater weight loss (1 study). Attendance was from 25% to 74% at 6 months. At 12 months attendance dropped dramatically from 10%-17%. Retention was greater than 60% at the end of 1 year in 5 studies and for over 1 year in another 4 studies. In general both adherence and retention decreased greatly over time. There also appears to be variation in dropout by treatment condition. Women in the Health Works for Women Study dropped out of a delayed-intervention control group faster that the intervention group, and the investigators offered part of the intervention 6 months early to retain them in the study. Reasons for dropping out of studies were travel and vacations, time constraints, wanting a "faster or fad" diet and health problems. Other reasons were lack of transportation, need for childcare, alcohol-related problems and other social factors, and high intervention staff turnover. | There are 12 main messages from this review which emphasize that process evaluation and decomposition of interventions aimed at both promoting a healthy diet and increasing physical activity are needed…Participants should be able to decide which change strategies are most appropriate for themselves. Sound health pedagogical principles and strategies for teaching need to be used. Moderating variables need to be addressed in study designs. Issues related to recruitment, retention and adherence need further attention to include integrity and fidelity of the intervention. The dose-response relationship need to be carefully examined as well as the therapeutic index or intervention safety factors. |
| Booth^55^  2014  Primary Care  SR-MA  15 Studies | Adults with overweight and obesity  (n=4539) | Behavioural  weight loss interventions | Face-to-face, telephone (some group) | HCP/ Project Staff/ Community Health Educators-Patient/ Participant | Primary outcome: weight loss | All of the studies reported change in weight at 12 months in the control and intervention groups, but not all of these could be included in a meta-analysis. The cluster trial which had not accounted for clustering in its analysis was excluded, and a second cluster trial reported mean baseline and follow-up weights, but these values couldn’t be used to calculate weight loss due to a drop-out rate of almost 40%. | The results of this review suggest that behavioural interventions conducted in primary care have a negligible effect on participants’ weight at 12 and 24 months. Weight loss interventions are generally considered to be clinically significant if participants lose ≥5% of their baseline total body weight (34). Based on the range of mean baseline body weights observed in this review (82.9–107.7kg) the pooled estimates did not reach this threshold. Whilst the key clinical finding from this review relates to the limited effectiveness of primary care behavioural interventions at achieving weight loss in participants living with obesity, the results also highlight a number of questions of importance for research. |

| Brown^56^  2008  Primary Care  Integrated Review  11 Studies | Adults with overweight or obesity  (n≈3149 nurses, 2 NR; n≈ patients 5256) | The role and practice of nurses in relation to weight management or other services provided to adults living with overweight or obesity | Face-to-face | PCN-patient | NS | NS | The NHS is a useful case study of how nursing practice can develop in response to the public health problem of obesity. A small number of empirical studies provide some insight into the roles, preparation and outcomes of nursing practice in managing obesity. The findings from these studies are relevant to other settings and health systems considering how to tackle obesity. They illustrate that this is a resource intensive development for primary care nursing but with potential to help patients tackle obesity. However, caution is needed. Despite progress towards a well-trained workforce making use of evidence-based protocols the actual outcomes of the activity remain contested. |
| --- | --- | --- | --- | --- | --- | --- | --- |
| Brown^57^  2011  Any setting  SR-QUAL  29 Studies | People living with overweight or obesity (n=1387) | Weight management decision-making | NR | HCP-Patient | Perceptions of participants living with  overweight or obesity. | Improving decisions about weight management requires attention to how diffuse cultural and psycho-social factors, such as obesity stigma, influence patient choices. Reflection on experiences of previous attempts at weight loss is also essential, as are practical resource factors – particularly for less affluent groups. Considering these factors along with more established theories of individual psychological motivations and barriers may help to improve initial participation and retention within interventions. | A systematic review of qualitative studies of obesity shows there are complex influences on weight management decisions. Improving decisions about weight management interventions requires attention to how the more diffuse cultural and psycho-social factors, such as obesity stigma, influence choices. Appreciating these broader factors, as well as the more established personal psychological motivations and barriers, may help to improve choices about interventions and hence retention within and better outcomes from weight management. It may also improve satisfaction with health professionals with respect to obesity management. |
| Burgess^58^  2017  NR  SR-QUAL  24 Studies | Adults living with obesity  (n=6563) | Lifestyle interventions | NS | HCP-Patient | Primary outcome: adherence to lifestyle intervention or lifestyle intervention programmes (objective or self-reported modifications to physical activity and/or diet or by lifestyle intervention programme session attendance or attrition | An increased focus on addressing common barriers to behaviour change, perhaps using a variety of behavioural treatment strategies, may facilitate long-term engagement and adherence for adults with obesity. Lifestyle intervention programmes that involve a MDT will be best able to help individuals with barriers to behaviour change and ultimately improve adherence and health outcomes.  The most prominent predictors of adherence identified were: early weight loss success, lower baseline BMI, better baseline mood, being male and older age.  he predictors of adherence reveal that early weight loss is crucial to longer-term adherence within a lifestyle intervention programme. It is proposed that people lose interest, motivation and confidence in themselves and the programme on offer when they do not experience early weight loss. The finding that individuals with lower expectations with regards to weight loss have better adherence rates is also an indicator that weight loss expectations play a huge role in adherence and affect the success of an intervention. We suggest that health professionals should be careful when discussing weight loss expectations with individuals in the initial stages of a programme to ensure that unrealistic goals do not increase the likelihood of attrition. Discussing the physical and psychological health benefits of long-term lifestyle change is also crucial to help steer people towards creating goals based on health and quality of life as opposed to the traditional and time-limited weight loss focus.  Many studies were not included within this review as they used weight change as their only marker of adherence, without any mention of factors such as attendance, attrition or lifestyle modification. | The findings within this review provide novel insight to clinicians working with individuals living with obesity and have important implications for lifestyle intervention programme design. Barriers to behaviour change need to be addressed early in treatment, with lifestyle intervention individualized accordingly. Predictors of adherence should also be taken into careful consideration, with negative moods and unrealistic weight loss expectations discussed at the outset.. If adherence were improved, treatment effectiveness, health outcomes and the ultimate burden of chronic disease could also be improved. |
| Byaruhanga^59^  2020  Any setting, including community and health care settings  SR-QUAL  13 Studies (5 obesity) | Obesity trials targeted clinical populations with lifestyle conditions such as diabetes, hypertension, and overweight or obesity (BMI>25 kg/m^2^)  (n=328) | Video communication was used to deliver individual, one-on-one support (ie, Skype, FaceTime, Facebook Messenger, WhatsApp, or any preferred individual real-time video communication platform). | Real-time video communication | HCP-Patient | Any measure of an individual’s smoking, nutrition alcohol, physical activity, or obesity | The retention rates at follow-up were 87.6% (496/566) at 12 months, 55% (27/49) and 97.4% (112/115) at 6 months, and 78% (38/49) at 3 months (refers to all real-time communication included in this review). | The overall results suggest that video counseling is neither more nor less effective in modifying smoking and alcohol consumption but may have particular benefits for addressing physical inactivity and obesity. |
| Comșa^60^  2020  NR  SR-MA  16 Studies | Healthy adults living with overweight or obesity (25<BMI>40)  (n=1663) | Active CBT intervention Accepted strategies: cognitive restructuring, problem solving, body image acceptance, weight expectations, self-efficacy, working on maintenance the weight loss. | Face-to-face | Psychologist/ counsellors/MDT-Patient | Primary or Secondary: Change in weight (BMI,% or WC) and cognitive component. Other psychological features such as behaviours and emotional states | NS | CBT is an efficient psychological treatment in weight loss. Its efficacy lies in adding the cognitive factors, more specifically motivation and self-efficacy. Our results show that CBT intervention is more effective when delivered by a MDT in longer sessions, since these two factors are significant moderators. |
| Conley^61^  2021  Various Settings (hospital outpatient clinic, research organisation)SR-MA  17 Studies | People with CKD living with overweight or obesity  (n=988) | Intentional weight loss interventions of at least four weeks in duration, designed to promote weight loss as one of their primary stated goals | NS | NS | Primary outcomes: Death (any cause), cardiovascular events, anthropometric measures  Secondary outcomes: Clinical parameters, patient-centred outcomes, cost effectiveness and economic analysis, adverse events and potential harms | Only five studies were considered to be at low risk of attrition bias. Three studies were assessed to be at high risk of bias as more than 20% of participants were lost to follow-up. Attrition bias in the remaining nine studies was judged unclear due to insufficient information.  There was limited reporting of adherence to treatment, adverse events, and potential harms across all three comparisons. | All types of weight loss interventions had uncertain effects on death and cardiovascular events among adults living with overweight and obesity and with CKD as no studies reported these outcome measures. Non-surgical weight loss interventions (predominately lifestyle) appear to be an effective treatment to reduce body weight, and LDL cholesterol. Surgical interventions probably reduce body weight, waist circumference, and fat mass. The current evidence is limited by the small number of included studies, as well as the significant heterogeneity and a high risk of bias in most studies. |
| Cradock^62^  2017  Community, clinic  SR-MA  13 Studies | >18 years of age with clinically confirmed type 2 diabetes  NR | Dietary and physical activity interventions | Face-to-face, app (some group, combination group-individual) | Primary outcome: HbA1c, Secondary outcome: Body weight | HCP/ Exercise Physiologist-Patient | Adherence/ Treatment fidelity | Our findings show that combined diet and physical activity interventions achieved clinically meaningful reductions in HbA1c at 3 and 6 months, but these were not sustained at 12 and 24 months. We identified four BCTs and nine intervention features associated with reductions in HbA1c. These exploratory findings may guide future research into BCTs such as ‘instruction on how to perform a behaviour’, ‘behavioural practice/rehearsal’, ‘action planning’, and ‘demonstration of the behaviour’ which seemed to be associated with better outcomes in type 2 diabetic adults in addition to the intervention features identified. |
| Crawford^63^  2012  NR  SR-QUAL  13 Studies | Participants who were aged over 18 and attempting to lose weight  (n=5771) | Weight loss intervention: behavioural weight-loss counselling; bariatric surgery; mail and telephone intervention | Mail and telephone | Expectations were assessed and analysed as part of the investigation with regard to a defined outcome (which could be in terms of weight loss, weight regain, and attendance/attrition) | Weight loss, weight regain. attendance/ attrition | Studies either suggested that there was no relationship or that higher expectations resulted in higher rates of attrition. Thus, potentially, higher expectations may be linked with higher weight loss in the mid-term but also with higher rates of attrition/attendance. | Studies exploring the relationship between expectations and short-term weight loss seem to suggest that there is no association. For mid-term weight loss, it is suggested that this relationship is unclear, with half of the studies reviewed suggesting there to be no association, whilst the other half suggested that higher expectations were related to higher weight loss. The relationship remains unclear for longer term weight loss with no clear agreement as to the relationship between expectation and weight loss. With regard to weight regain, all studies reviewed suggested there to be no association between expectations and weight regain. The overall quality of the studies reviewed was reasonably high, with ratings ranging from 16 to 19 out of 20. The majority of papers were rated as lower in quality due to not reporting power calculations or effect sizes. |
| Dombrowski^64^  2012  Community, Primary Care  SR-MA  33 Studies | Adults living with obesity and additional risk factors or co-morbidities.  (n=10055) | Behavioural interventions aimed at changing diet and/or PA | Face-to-face (some group) | HCP/Non-HCP-Patient | Primary outcome: Behaviour and weight change Secondary outcome: modes of intervention delivery and BCTs. Reporting of behaviour-change data for diet and PA | A mean dropout at study completion of 16% (SD=10.2; range 0-47.1).  Twenty-one studies provided numbers and reasons for study participant dropouts and 20 studies mentioned the numbers of withdrawals only. Three studies stated withdrawals but did not provide further details regarding numbers and reasons. | Meta-regression suggested that increasing numbers of identified BCTs are not necessarily associated with better outcomes. The BCTs provision of instructions (β=-2.69, p=0.02), self-monitoring (β=-3.37, p<0.001), relapse prevention (β=-2.63, p=0.02) and prompting practice (β=-3.63, p<0.001) could be linked to more successful interventions. Studies including more BCTs aimed at dietary change that are congruent with Control Theory were associated with greater weight loss (β=-1.13, p=0.04). Post-hoc ratings of intervention components in published trials can lead to the identification of components and theories for behaviour change practice and research |
| Dombrowski^65^  2014  Home, clinic, community  SR-MA  42 Studies | Adults (aged ≥18, BMI of ≥30 and lost ≥5% of their body weight/mass within 24 months before weight loss maintenance treatment.  (n=9451) | Any behavioural/lifestyle, pharmacological (with European Medicines Agency approval for weight loss), food replacement/supplement, or alternative interventions, singly or in combination were included | Face-to-face, remote (internet, telephone) | HCP/peers/students-patients | Primary outcome: weight at 12 months from randomisation to the weight loss maintenance intervention. | Initial attrition was mainly because of study dropout, although failure to meet weight loss and/or adherence criteria prevented entry into the maintenance phase for individuals in 17 studies. A total of 6278 individuals completed weight maintenance treatment (additional average dropout of 20%). | Comprehensive behavioural interventions targeting dietary and physical activity behaviours are moderately effective in slowing regain of weight in adults living with obesity after initial weight loss for follow-up periods of up to 24 months. Orlistat treatment in addition to behaviour change is effective in reducing weight regain, with clear evidence of a dose-response relation and some evidence for effectiveness over 36 months. Side effects of this drug should be considered and discussed with patients before it is prescribed |
| Dounavi^66^  2019  NR  SR-MA  39 Studies | Adults with typical intellectual ability  (n=26521) | Mobile health applications in weight management | Mobile technology (apps) including self-monitoring strategies, face-to-face | Researcher-Participant | Weight management behaviours | mHealth apps can be effective in the self-management of weight, such as in reducing weight, and improving health indicators, such as glucose levels and BP, provided specific characteristics identified in RCTs as effective are incorporated. These include the effortless self-monitoring of diet and physical activity, tailored feedback provision, reminders for app use, and interaction with peers. The integration of such features increases user engagement with the treatment, which, in turn, promotes successful weight management  The examination of social validity indicators reveals a pattern where higher engagement with mHealth applications is associated with greater treatment adherence and consequent weight loss. | mHealth applications seem to facilitate weight management across a wide range of measured outcomes. There is sufficient consensus across studies that mHealth apps are acceptable by patients and effective in producing weight loss through lifestyle changes in eating behaviours and physical activity patterns. The examination of social validity indicators reveals a pattern where higher engagement with mHealth applications is associated with greater treatment adherence and consequent weight loss. This study also highlights the need to obtain further empirical evidence about the role of social validity as a mechanism underlying the influence of mHealth app use on weight management behaviours. |
| Everitt^67^  2021  OPD, Clinic. Community, University  SR-QUAL  17 Studies | Adults living with obesity (BMI ≥30kg/M2)  (n=14166) | Multidisciplinary (MD) non-surgical WMIs designed to provide a minimum of 6-months treatment. | NS | NS | Attrition/ retention | The studies reviewed suggest older age, living in less deprived areas, higher levels of education and female gender were the sociodemographic factors with evidence of reduced risk of attrition. There was some, but limited evidence that poor mental health, low social support, high weight loss goals and poor or unsatisfactory results may increase the likelihood of dropout. Until a comprehensive understanding of attrition is developed, WMIs should focus on reducing social inequities in the benefit of WMI provision. | The scope for targeted retention strategies is limited because few variables were consistently associated with attrition. Until a comprehensive understanding of attrition emerges, WMIs should seek to reduce social inequities in the benefit of WMI provision. Future research should consider factors reported qualitatively, such as intervention expectations and satisfaction, social support, patient-clinician relationships, and logistical barriers. Adopting a universal definition of attrition and de-homogenising participant dropouts would advance future research. As qualitative evidence is limited, exploring participant experiences of WMIs would help understand how attrition rates can be reduced, and in-turn improve WMI effectiveness. |
| Faulkner  2007  Any setting  SR-MA  23 | People diagnosed with schizophrenia or schizophrenia-like illnesses  (n>1000) | Weight loss (treatment) and weight maintenance (prevention) studies evaluating pharmacologic or non-pharmacologic adjunctive interventions | NS | Primary outcome:  Weight loss or maintenance, Global measures, Mental state, Wellbeing and QOL, Adverse effects - general and specific, Leaving the study early | Weight, Global Measure, Mental State, Wellbeing and QOL, Adverse Events, Leaving the study early | Prevention - CBI v. Standard Care: One small study (Cog/Behav 2003) managed to retain all its participants. Whilst (Cog/Behav 2005b) initially indicating better retention in the CBI group but this was not sustained during further follow up at six months after the study began. Treatment – CBT v. Standard Care The additional intervention of CBI does not improve or reduce attrition rates relative to the control group during medium term. | Modest weight loss can be achieved with selective pharmacological and non pharmacological interventions. However, interpretation is limited by the small number of studies, small sample size, short study duration and by variability of the interventions themselves, their intensity and duration.. At this stage, there is insufficient evidence to support the general use of pharmacological interventions for weight management in people with schizophrenia. |
| Ge^68^  2020  NR  SR-MA  121 Studies | Adults (≥18 years) living with overweight (BMI 25-29) or obesity (BMI≥30)  (n=21942) | Popular named dietary programmes | NS | NS | Weight loss, lipid profile, BP, CRP levels | Loss to follow-up | Compared with usual diet, moderate certainty evidence supports modest weight loss and substantial reductions in systolic and diastolic blood pressure for low carbohydrate (eg, Atkins, Zone), low fat (eg, Ornish), and moderate macronutrient (eg, DASH, Mediterranean) diets at six but not 12 months. Differences between diets are, however, generally trivial to small, implying that people can choose the diet they prefer from among many of the available diets without concern about the magnitude of benefits. |
| Gilinsky^69^  2015  Hospital  SR-MA  13 Studies | Women with previous diagnosis of GDM  (n=2746) | Lifestyle intervention (promoting weight loss or physical activity, change in diet, or decreasing sedentary behaviour and delivered via structured exercise programmes, lifestyle counselling, health education, and self-management programmes) | Face-to-face, online | HCP/ Peer Educators-Patient | Behavioural, anthropometric or diabetes outcome | Retention rate at the last follow-up point in the included studies was generally between 80–100%. Three studies reported good retention at later follow-ups (i.e. >90% at three years and one study at 51 months. Limited details were provided on reasons for loss to follow-up or methods used to retain participants across most studies. Two studies reported lower retention i.e. 77% and 68% at six months and 12 weeks. | This review shows that we currently lack an evidence base from methodologically robust trials for how to effectively promote lifestyle change among women with prior GDM. There is evidence quality of methodology is improving with future study protocols providing more detailed information and being methodologically more robust. Recruitment to trials and adopting lifestyle changes appear challenging in this group. |
| Hartmann-Boyce^70^  2021  Any setting  SR-MA  249 Studies | Non-pregnant adults (aged ≥18) living with overweight or obesity (BMI ≥25, or ≥23 in Asian populations)  (n=59081) | Behavioural weight loss programmes | Face-to-face, web-based, telephone (some group) | Coach/ Dietitian-Patient | Rate of change in weight after the end of the programme | Attendance at behavioural weight loss programmes declines over time and many participants struggle to maintain the cognitive effort required to adhere to ongoing interventions. | Regain in weight after weight loss in a weight management programme was faster with greater initial weight loss, but it takes an average of five years to remove the weight advantage (and thereby the beneficial effect on cardiometabolic risk) that greater initial weight loss achieves (fig 2). Such legacy benefits for ongoing, although declining, reduction in risk factors for cardiovascular disease have been shown to improve major clinical outcomes in studies of interventions for hypertension and high lipid levels. We found no evidence that providing support for specific maintenance of weight loss during the programme reduced the regain in weight. Instead, programmes that are widely available to allow individuals to re-engage when they want to might be the best strategy for supporting longer term lower rates of change in weight |
| Hutchesson^71^  2015  NR  SR-MA  84 Studies | Adults aged ≥18 years  (n=24010) | e-Health Interventions (Internet, computers, including tablets, email, personal digital assistants (PDAs), mobile/smartphones, and digital games) | Digital, non-eHealth components delivered in written/paper-based form, telephone face-to-face (some group) | NS | Weight-related primary outcome (e.g. weight, BMI, percentage body fat, WC) | The mean retention rate at post-intervention was 78 ± 17 (range 16–98%), with 58.3% (n = 49) studies with retention rates ≥80% at post-intervention.  The potential impact of the eHealth interventions with additional features or technologies on other key outcomes such as participant engagement (e.g retention, behaviour change, along with the small, but significant amount of additional weight loss achieved (i.e. 1.5 kg greater weight loss) provide some support for preferential use of such eHealth interventions for obesity treatment.  In studies comparing eHealth weight loss interventions to minimal interventions, shorter intervention durations (<6 months) were shown to demonstrate significantly greater MD in weight change between groups, than interventions 6 months or more. This is consistent with traditional weight loss programmes (93), which are also more effective in the short term. For eHealth interventions specifically, it is likely because of the well-documented poor intervention engagement with eHealth interventions, whereby usage declines over the longer term (101). Many of the studies focused on evaluating ‘additional features’ because these features may facilitate engagement over time. These findings highlight the potential influence of user engagement on intervention effectiveness. | eHealth weight loss interventions achieve modest weight loss (−1.4 to −2.7 kg) at post-intervention compared with no or minimal treatment, and therefore offer an additional obesity treatment option. In addition, eHealth weight loss interventions with evidence-based behavioural features (e.g. self-monitoring, personalized feedback) also appear to achieve significantly greater weight loss (−1.5 kg). However, at this point, no specific recommendations can be made regarding what intervention components that are necessary for successful weight loss. Despite some promising results from individual studies, there is insufficient evidence to recommend the use of eHealth interventions for weight loss maintenance or weight gain prevention. Overall, while the evidence is inconclusive, current research suggests that individuals considering eHealth weight loss interventions should be encouraged to choose programmes with evidence-based behavioural features (e.g. selfmonitoring, personalized feedback, social support) that incorporate monitoring devices (e.g. accelerometers) for self-monitoring. To potentially improve accountability individuals may choose a programme that incorporates non-eHealth components (e.g. face-to-face appointments, telephone counselling) although these may not be imperative to success. |
|  |  |  |  |  |  |  |  |
| Lemstra^72^  2016  NR  SR-MA  27 Studies | Adults living with overweight and obesity (n=6803) | Weight loss or reduction interventions (supervised or non-supervised) | Face-to-face, Text (some group) | NS | Adherence or behavior modification (Adherence defined as completion of the WLP) | Among RCTs, the mean adherence was 63.1%, while the mean adherence for the observational intervention studies was 59.6%.  High adherence rates were observed for interventions that incorporated aspects of social support. Programs supervising and monitoring attendance improved adherence rates by 65% compared to self-monitoring programs and had the highest adherence rates overall. Monitoring attendance likely improves adherence rates since participants are more accountable for their behaviors. Supervision has been found to increase physical activity intervention adherence in other conditions.  Supervised programs also allow participants to access HCP knowledge and feedback. Working without guidance can be discouraging and, at times, dangerous.  Increasing knowledge transfer through access to an expert supervisor may help improve self-efficacy. A good relationship between supervisors and participants can increase adherence to additional health advice. | Supervising WLPs and adding social supports help improve adherence to weight loss programs. It is expected that with better adherence, people living with overweight or obesity can lose more weight and help keep it off in the long term. Evaluating the views of participants and adherence rates to weight loss programs is critical as it can offer insight, expand our current knowledge, and provide evidence in support of designing and implementing more effective interventions.(n=6) |
| Leung^73^  2017  Community, Clinic  SR-QUAL  19 Studies | Adults living with overweight or obesity (generally healthy without existing chronic diseases, significant psychological comorbidities or medical condition that limited the ability to perform PA)  (n=14082) | Lifestyle modification programs for weight management (clearly defined with components of diet, PA and behavioural strategies or theories. The diet component should be based on healthy diet principles and not involve meal replacement, low calorie diet or VLCD) | Face-to-face, web-based (some group) | HCP/ trained lay educators-Patients | Weight loss, weight maintenance and adherence | The most common indicator of adherence used was attrition, followed by attendance and self-monitoring. Only one study used dietary adherence as the indicator of adherence.  See Conclusion | Research on adherence to lifestyle modification is still at its infancy. We have reviewed a wide range of potential factors related to adherence. Of the 19 studies identified, attrition is the most common indicator used, followed by attendance, self-monitoring and dietary adherence. Factors that may predict better adherence were being in action or maintenance stage of change, older age, higher education, healthier eating and PA behaviour at baseline and more initial weight loss. Factors that may predict poor adherence were depression, stress, strong body shape concern, more previous weight loss attempts and unemployment. Inconsistent findings were found for self-efficacy, motivation and male gender. Despite our conclusions were limited by small number of studies identified for each factor and inconsistent results across studies, our attempt contributes to the synthesis of current knowledge on adherence to lifestyle modification program |
|  |  |  |  |  |  |  |  |
| Malik^74^  2017  NR  Integrative Review  38 Studies | Community pharmacists and nutritionists, patients living with obesity  (NR) | Weight loss counselling | NS | HCP (Pharmacists and nutritionists)-Patients | NS | Pharmacy based programs have been reported more effective than GPs, with a considerably greater retention rate of participants and weight maintenance after weight loss. However, lack of time, patient demand, and incentives are few of the identified barriers towards regular counseling by the pharmacists. | Rapid urbanization, lifestyle modification, high energy dense diets, and minimal physical activity are identified factors continuously contributing toward obesity crisis globally. Screening and treatment for diseases get delayed as individuals living with overweight are mostly reluctant to seek medical care for their obesity, as they are certain of their humiliation. Physical exercise and dietary measures to control body weight are lacking in spite of the community desire to have appropriate body weight. This is due to lack of awareness among community regarding healthy lifestyle and role of health-care professionals in weight management. Patient education programs to control obesity have been ignored. The role of community pharmacists and nutritionists in obesity management is not well-acknowledged in most of the developing countries including Pakistan. Limited data are available on number of nutritionists and type of services provided by them for weight management in these countries. |
| Mastellos^75^  2014  Hospital, Community  SR-QUAL  3 Studies | Adults, age 18 years and over living with overweight or obesity according to any standard parameters used by the WHO. Participants with co-morbidities, such as diabetes, heart diseases and hypertension  (n=2971). | Dietary intervention or physical activity interventions, or both, and other interventions based on the TTM SOC. | Telephone, mail | HCP-Patient | Primary outcome: Sustained weight loss (1-5 years and above), Short-term weight loss (<12 months), HRQOL Secondary outcome: Change in: dietary consumption, physical activity, other weight loss measures, SOC progression. | The included trials addressed the incomplete data for subjective outcomes by incorporating imputation techniques (Johnson 2008; Jones 2003; Logue 2005). However, one trial had high attrition rates (in total, only 54% in the intervention group and 67% in the control group finished the trial) and was characterised 'unclear risk' with regards to this domain (Johnson 2008). The other two trials were categorised ‘low risk’ in terms of attrition bias (Jones 2003; Logue 2005). | The evidence to support the use of TTM SOC in weight loss interventions is limited by risk of bias and imprecision, not allowing firm conclusions to be drawn. When combined with diet or physical activity, or both, and other interventions we found very low quality evidence that it might lead to better dietary and physical activity habits. |
| Metzendorf^76^ 2022  Community, Primary Care,  Outpatients  SR-MA  18 studies | Adults with mean BMI 27.3-49.6kg/m^2^; (adolescents – 2 studies)  (n=2703) | Mobile Health (M-Health) Smartphone Interventions | Online/ Remote (App); In person sessions | HCP-Patient;  Researcher-Participant | Primary outcomes: change in physical activity, change in anthropometric measures, Adverse events  Secondary outcomes: Health-related quality of life, self-efficacy, well-being, participation satisfaction, adherence/engagement, change in dietary behaviour | 14/18 studies were judged as high risk due to “missing outcome data (substantial loss to follow-up, even in studies with a duration of three months), deviations from the intended interventions (due to lack of blinding, dropouts and substantial loss to follow-up) or  bias in the measurement of the outcome (knowledge of assigned  intervention could influence outcome measurement. | The available evidence is limited and does not demonstrate a clear benefit of smartphone applications as interventions for adolescents or adults with overweight or obesity. While the number of studies is growing, the evidence remains incomplete due to the high variability of the apps' features, content and components, which complicates direct comparisons and assessment of their effectiveness. Comparisons with either no or minimal intervention or personal coaching show minor effects, which are mostly not clinically significant. Minimal data for adolescents also warrants further research. Evidence is also scarce for low- and middle-income countries as well as for people with different socio-economic and cultural backgrounds. The 34 ongoing studies suggest sustained interest in the topic, with new evidence expected to emerge within the next two years. In practice, clinicians and healthcare practitioners should carefully consider the potential benefits, limitations, and evolving research when recommending smartphone apps to adolescents and adults with overweight or obesity. |
| Moroshko^77^  2011  Any setting  SR-QUAL  61 Studies | Adult (18–65 years) living with over-weight or obesity (BMI ≥25)  (n=14531) | Weight loss interventions (e.g. such as diet, exercise, behaviour modification, cognitive behavioural therapy, lifestyle intervention, pharmacotherapy and surgery) | Face-to-face (some group) | NS | Attrition | Increased attrition was associated with individual treatment mode in one study and with group treatment mode in another. In contrast, another study failed to find association between attrition and a counselling compatibility. | Treatment dropout is not consistently reported or explored in WLIs. Conclusions were limited by the large number of variables considered, very small number of studies exploring each variable and by inconsistent results across studies. Trends and findings of the current review can be used to generate hypotheses to be examined in future research. More comprehensive assessment and reporting of theoretically grounded dropout correlates and predictors are required. Patient’s mental health and social–psychological processes associated with participating in a weight loss treatment are predictive of attrition and thus worthy of further research. Such constructs typically require more comprehensive examination and may need to be assessed both prior to and during treatment. |
| Naude^78^  2022  Outpatient clinics, medical/ research centres  SR-MA  61 Studies (58 in MA) | Adults (18+years old) living with overweight or obesity with or without T2DM, cardiovascular conditions or risk factors such as hypertension or dyslipidaemia  (n=6880) | Low-carbohydrate weight-reducing diets | Face-to-face, telephone (some group) | HCP (dietitian)-Patient | Primary outcomes: Change in body weight (kg), participants per group with weight loss ≥5%  Secondary outcomes: adverse events | For interventions in which there was substantial attrition (15% or more for at least one of the groups) of study participants the attrition rate was reported. Sensitivity analyses excluded studies with some concerns or a high risk of bias for the missing outcome data domain in RoB2. | There is probably little to no difference in weight reduction and changes in cardiovascular risk factors up to two years' follow-up, when participants living with overweight and obesity without and with T2DM are randomised to either low-carbohydrate or balanced-carbohydrate weight reducing diets. |
| Norris  2005  NR  SR-MA  9 Studies | Adults with prediabetes  (n=1568) | Dietary, physical activity, and behavioral interventions | NS | NS | Primary outcomes: weight, BMI, or % weight loss  mortality: diabetes-related, CVD, total; QOL Secondary outcomes: morbidity; CVD events; glycemic control, serum lipid concentrations, BP | Attrition ranged between 4% (at one-year follow-up) (Lindahl 1999) and 43% (10-year follow-up) (Jarrett 1979).  Mean attrition was 9.6%, perhaps because participants were most commonly self-selected and thus perhaps more motivated to complete the study. Jarrett et al (1987) noted sustained weight loss at 10-year follow-up, but their attrition rate was 43%, which likely contributed to the large measured weight loss among participants who persisted with the intervention over the long term. The successful interventions we examined in this review (DPP 2002; Pan 1997; Tuomilehto 2001) had prolonged frequent contacts, low attrition rates, and evidence of persisting behaviour change, which likely contributed to the effectiveness of the interventions. Few data are available on changes in patterns of behaviours over time. | Overall, weight loss strategies using dietary, physical activity, or behavioural interventions produced significant improvements in weight among persons with prediabetes and a significant decrease in diabetes incidence. Further work is needed on the long-term effects of these interventions on morbidity and mortality and on how to implement these interventions in diverse community settings. |
| Park^79^  2019  NR  SR-MA  20 Studies | Adults living with overweight or obesity over 18 years of age with a BMI above 25 kg/m2  (n=2318) | Mobile healthcare (m-health) programs providing health promotion services and health information through mobile phones | Mobile phone | NS | Changes in body weight and BMI | One study showed a dropout rate of more than 20% during the follow-up period, but this was a long-term follow-up period of 1 year | The results of this study showed that mHealth intervention for adults living with obesity led to a modest short-term effect on body weight and BMI. The mobile phone provides convenience in everyday life; weight-loss options using mHealth have recently expanded. It is therefore difficult to definitively determine the effect of mHealth at this point. In the future, mHealth is expected to have a significant impact on reducing adult obesity, given improved service content available through mHealth, the convenience of the mobile phone, and mHealth’s ability to actively intervene in the daily life of adults living with obesity in real time. |
| Plotnikoff^80^  2015  Tertiary Education  SR-QUAL  17 Studies | Staff of institutions within the tertiary education sector  NR | Interventions implemented in a tertiary education setting and have an aim of improving one or more health behaviors, including but not limited to physical activity, dietary intake, weight loss, smoking patterns, alcohol consumption, sexual health, and/or mental health. | Face-to-face, telephone | NS | Physical activity-, Eating behavour-, weight-, health risk-, mental health–related outcomes. Ergonomics, Other risky behavior outcomes | Retention was poor at 31% in the second largest cohort study; three studies had retention between 60% and 79% and 12 studies, >80%; one study did not report retention. | Research is currently lacking in the field of health promotion programs for tertiary education staff, despite the need for and potential of such programs. Whilst this is the case, there is extensive literature on health promotion programs within other workplace settings that can contribute to the knowledge base on this topic. Universities and colleges are important and unique workplace environments for promoting health. They comprise an abundance of research expertise and often include world-class facilities and resources across a vast array of health-related disciplines. This potentially places tertiary organizations at the forefront of workplace health promotion. |
| Power^81^  2025  Mixed Settings: Clinical, community and research  SR-MA  47 studies | Adults (aged 18 years+) living in any country with a BMI>25 kg/m^2^  (n=6729) | Emotional Eating Interventions | Face-to-face; remote; group and individual | HCP-Patient; Researcher-Participant | Changes in weight, Emotional Eating | NR | This is the first systematic review and meta‐analysis to examine how specific BCTs impact EE in adults living with overweight or obesity. Overall, interventions were associated with a significant reduction in weight loss and EE scores, but the substantial heterogeneity indicates that effectiveness varies widely and therefore there is low certainty of evidence. Further testing of the BCTs identified in this review, using high‐quality RCT design, is needed to strengthen confidence in the results. Future intervention studies addressing EE and weight should consider including the following BCTs as a minimum: ‘incompatible beliefs’, ‘goal setting (outcome)’, ‘pros/cons’, ‘review outcome goal’ and ‘feedback on behaviour’. Interventions may also benefit from the inclusion of ‘commitment’, ‘goal setting behaviour’, ‘information about emotional consequences’, ‘distraction’, ‘self as role model’, 'problem solving’ and ‘social support unspecified’. Interventions that address EE only should consider the following additional BCTs: ‘social support (emotional), ‘information about health consequences’, ‘remove aversive stimulus’ and ‘monitoring emotional consequences’. |
| Puig^82^  2019  NR  SR-QUAL  28 Studies | Any population  (NR) | mHealth-based interventions for overweight or obesity management | Mobile, Face-to-face | NS | Reduction of weight and BMI as well as fat mass and hip circumferences, changes in dietary habits, PA and screen time patterns; biochemical measurements, BP | Although the main aim of most of the studies was to measure the efficacy of mHealth interventions, they also measured other outputs that might be relevant for determining the success of the intervention, such as (1) acceptability, (2) adherence, usability, and engagement, (3) satisfaction, (4) motivation, (5) intention to continue, and (6) perceived support. All these aspects appear to affect whether an intervention will be successful. Tests and questionnaires are the most prevalent tools used for assessment, whether existing and previously validated or devised for the situation | The potential for apps to positively help users manage their obesity or overweight has yet to be attained. Studies assessing the success of mHealth interventions are remarkably heterogeneous and most have methodological limitations that leave significant room for improvement regarding quality. |
| Reed^83^  2012  Workplace, Academia, Clinic  SR-MA  11 Studies | Adults living with overweight or obesity  (n=1866) | Computer-based education or support aimed at reducing weight or BMI | Computer-based (website, computer programme), Face-to-face (some group) | NS | Change in weight and/or BMI | Inadequate detail regarding randomisation techniques and attrition precluded assessment of selection and attrition biases. | While the addition of computer-based technology to weight loss interventions led to statistically greater weight loss, the magnitude (<1.5kg) was small and unsustained. |
| Renders^84^  2000  Primary Care, Outpatients, Community  SR-QUAL  41 Studies | HCPs (including physicians, nurses, pharmacists), taking care of non-hospitalised patients with T1DM; T2DM in a PC, OPD  (n=48000 patients, 200 practices) | Intervention strategies to improve the care for patients with diabetes, including organisational, professional and financial interventions | Face-to-face | HCP (Physicians/ Nurses/ Pharmacists)-Patients | Objectively measured health professional performance or patient outcomes in a clinical setting and self-report measures with known validity and reliability | Among patients who entered the study high dropout rates were experienced, which may have affected the reported effects. There are many factors that predispose to non-attendance e.g. patient health beliefs, attitudes of health professionals and financial costs of attendance. On the other hand, because diabetes affects a population of mainly elderly people with a chronic complex disease, some loss to follow-up is inevitable in primary care to follow-up because of severe illness, death and hospitalisation | From the review it is difficult to know whether the postgraduate education components did contribute to improvements in care. There were no trials solely evaluating postgraduate education - all had other components. Postgraduate education in combination with other professional interventions improves the process of diabetes care. Also strategies that increase structured recall contribute to a better quality of diabetes care. This can be achieved by central computerised tracking systems or by nurses who regularly contact patients. These arrangements for follow-up improve process outcomes. The effectiveness of these professional and organisational interventions on patient outcomes (clinical outcomes and wellbeing) is less clear. The addition of patient education or a more enhanced role of a nurse to a complex intervention strategy seems to be important to improve patient outcomes besides process outcomes. Nurses can play an important role in facilitating compliance or giving patient education. They can even replace physicians in delivering many aspects of diabetes care, if detailed management protocols are available, or if they receive training. |
| Shaikh^85^  2020  Various Settings  SR-MA  20 Studies | Breast cancer survivors diagnosed with early stage breast cancer with no evidence of a recurrence of their cancer living with overweight or obesity  (n=2360) | All weight loss interventions (diet, diet & exercise, diet & psychosocial, diet & exercise & psychosocial) | Face-to-face, online, telephone (some group) | NS | Primary outcome: Overall survival, Breast cancer recurrence Secondary outcome: Change in body weight, BMI, skinfold thickness, WC, Disease-free survival, Adverse events, Change in QOL and PROs, Changes in concentration of oestradiol, androgen, insulin, IGF, FG and lipids profile, Changes in adipokine concentrations, Changes in inflammatory marker concentrations | From these 20 studies, 2360 breast cancer survivors were randomised, and we analysed 2028 (85.9%) of these participants. The remaining 332 (14.1%) participants were unable to be analysed primarily due to attrition. The attrition rate is in part explained by our decision to include data on the latest follow-up provided for each study. The ratio of patients analysed to patients randomised varied considerably by outcome, with ranges of 77.4% to 80.4% for anthropometric outcomes, 52.7% to 73.8% for biomarker outcomes and 73.5% to 92.5% for QOL outcomes. The number of intervention-comparisons (and studies) analysed per outcome was also highly variable, with several outcomes having an insufficient number of intervention-comparisons to be included in the meta-analyses.  There was also evidence of attrition bias, with anthropometric outcomes having attrition rates ranging from 19.6% to 22.6%. | This review identified insufficient data to reach firm conclusions regarding survival outcomes and breast cancer recurrence after body weight interventions breast cancer survivors living with overweight and obesity. Whilst there was sufficient data to perform meta-analyses on breast cancer recurrence (which suggested no evidence of effect), the total number of included intervention-comparisons, participants and recurrence events were very small (and thus likely underpowered), and the quality of the evidence was low. The effect of these interventions on survival outcomes and cancer recurrence remain unclear and clinical recommendations cannot be made when considering these primary outcomes in isolation. Our secondary outcomes suggest that these body weight interventions compared favourably to controls (which included usual care) and resulted in significant improvements in anthropometric outcomes, and were safe. Whilst the quality of the evidence was high for safety (adverse events), it was low for the remaining outcomes (change in body weight, waist circumference, overall QOL) due to a high risk of bias and substantial heterogeneity. In contrast, there appeared to be no evidence of effect of these weight loss interventions on other QOL subscales (social subscales, emotional subscales and anxiety and depression subscales). There also appeared to be no evidence of effect for analysed biomarkers, with the exception of triglycerides and leptin. However biomarker analysis may have been underpowered as the interventions may not have been of sufficient duration and/or intensity to cause a detectable decrease in biomarkers. There was some evidence to suggest using a combination of different intervention modalities (e.g. 'diet and exercise' or 'diet and exercise and psychosocial support') may lead to a greater effect than an exclusively 'diet' intervention. However, there was a wide range of intervention programmes using multimodal interventions, and it is not known which particular intervention programme confers the most benefit. Thus, whilst the overall quality of evidence was low and the effects on survival and recurrence are unclear, the interventions were safe and were associated with favourable improvements in anthropometric outcomes and various aspects of QOL. Therefore clinicians may consider utilising such interventions in their clinical practice, although no definite recommendations can be made from this current review. |
| Shannon^86^  2009  NR  SR-QUAL  23 Studies | Adult participants (aged 18 years or older)  NR | Management of obesity using some form of digital technology | Mobile phone applications and text messages, smartphone, or telehealth/ telemedicine, telephone counselling (videoconferencing, and interactive telemonitoring), e-health (websites and internet-based programs), wearable device | NS | Primary outcomes: Weight loss, physical activity, dietary intake habits, time spent in sedentary positions Secondary outcomes: changes in self-efficacy, BP, step count, adherence to prescribed technology during the study | User experience and technology usability are critical factors, especially for interventional studies. The technology adoption model theory suggests that usefulness and ease of use are factors associated with technology adoption and should be identified in interventional studies.12, 13 When intervention outcome effects are examined, technology and participant acceptance should be considered. If the technology involved is difficult to use, inefficient, or ineffective, all of these could cause user dissatisfaction and noncompliance, thereby contributing to nonachievement of the goal that the study was intended to measure. Only two of the reviewed studies measured technology use and user satisfaction as outcome measures. Further study exploring associations between the user experience of technology and outcome changes could fill the gap in this area.  Future research needs to include patient-level outcomes as well as cost-effectiveness and user engagement of technology to fully evaluate the feasibility of continued and expanded use of technology in obesity management. Future studies should also identify consumers’ individual and environmental factors, including consumers’ motivation to use a given technology, in order to evaluate whether these factors are correlated with the technology used and with weight loss. | Adoption of technology such as mobile health, eHealth, and telehealth/ telemedicine in obesity management has shown positive outcomes and wide usage. Weight loss and behaviour changes are among the measurements evaluated in studies of technology use for obesity intervention. In an environment of continual development and refinement of technology, and the advent of new software to apply those developments, this field of research has a positive future. Appropriate and effective technology use, in the hands of skilled researchers, promises more positive outcomes in obesity management and intervention. |
| Shaw^87^  2012  NR  SR-QUAL  14 Studies | Participants who are managing their weight  (n=2248) | SMS interventions reducing obesity, overweight or promoting weight loss | SMS primary mode of communication, face-to-face, website, wearable device | NS | Weight loss (body weight, BMI, WC physical activity or diet | From these results, effectiveness and optimal use cannot yet be determined. Nonetheless, at least one SMS per day may be appropriate in helping motivate people to engage in weight loss behaviours without generating a considerable burden. | Text messaging and mobile telephone technology have emerged as an encouraging tool in promoting health that can reach people directly wherever they are, that is affordable and is easy to use. Quickly becoming a popular and major area of focus, SMS is part of a larger field of mobile health, known as mHealth: the practice of medicine, nursing and public health supported by mobile devices. The US National Institutes of Health (NIH) alone have a dedicated mHealth research focus which partners with several NIH Institutes. Other public institutes such as the Centers for Disease Control offer free mobile tips and alerts through SMS on how to improve your health. Private corporations, such as Microsoft, have recently held a healthy apps competition where developers compete to create the best health focus application that can be run on a mobile phone. As more sophisticated mobile devices such as smart phones become ubiquitous, SMS will be just a part of a cadre of mHealth technologies that will become available. This affords the opportunity for research applications that were not previously available, such as simultaneously assessing behavioural, physiological and psychological states in the real world and in real-time. The use of mobile technology, including SMS, affords numerous methodologic advantages over traditional methods, including ‘reduced memory bias, the ability to capture time-intensive longitudinal data, date- and time-stamped data, and the potential for personalizing information in real-time’ |
| Smith^88^  2020  NR  SR-QUAL  5 Studies | Participants enrolled in an online physical activity intervention for weight-loss (n=1099) | Online physical activity intervention for  weight-loss | Online (including both online and face-to-face) | Researcher-participant | Intervention usage data  throughout the intervention, and evaluated the dose-response relationship | The purpose of this review was to examine the types of intervention usage metrics reported in online physical activity interventions for weight-loss and to describe the dose-response relationship between usage and intervention outcomes. We found total logins, login frequency, and use of online tools were reported most frequently. We found that increased intervention usage was associated with an improvement in satisfaction with, fitness. MVPA and retention, and prevented weight regain following an intervention. Our review identified that there is a limited number of studies examining the dose-response relationship in online physical activity interventions for weight-loss and highlighted the importance to promote intervention engagement. | The objective of this review was to examine the types of intervention usage metrics reported in online physical activity interventions for weight-loss and to describe the dose-response relationship between usage and intervention outcomes. The usage metrics reported in online physical activity interventions for weight-loss included login frequency, login duration, and use of online tools. We found that increased intervention usage appeared to be associated with an improvement in participants' weight-loss, weight regain, physical activity behaviours, intervention retention. However, these findings should be interpreted with caution due to the limited number of studies. |
| Spanos^89^  2013  Community, Institutional  SR-QUAL  22 Studies | Adults (≥ 18 years of age) with a valid diagnosis of ID  (n=777) | Non-surgical or pharmacological interventions | Face-to-face  (majority group) | HCP-Patient | Impact of intervention on total body weight and/or BMI | The majority of the studies included in this review did not report a high dropout or attrition rate with the exception of one dietary intervention and a multi-component intervention. | The majority of the interventions were delivered in group sessions, which could be regarded as more preferable potentially offering improved cost effectiveness. However, there is insufficient evidence to support the effectiveness of group therapy for weight management versus individual therapy. No studies in this review explored or commented on which method is the most suitable way of delivering a weight loss intervention for adults with ID. |
| Streb^90^  2024  Clinical research settings  SR-QUANT  21 studies | Adults (aged 18-65 years) living with obesity  NR | Exercise based interventions | Supervised or unsupervised; Telephone | Qualified Professionals-Participants | Exercise adherence | The adherence percentage ranged from 18% to 99% of the prescribed exercise sessions. | The synthesis of exercise interventions for adults with obesity allowed us to conclude that interventions with adherence greater than 50% applied combined training supervised by professionals in the area, involving moderate or vigorous intensity exercises and interval training. Moreover, strategies incorporating social support— particularly counselling and group activities—were associated with higher attendance rates for exercise sessions in this population. |
| Suire^91^  2021  Varied Settings  SR-MA  9 Studies | Women  (n=1034) | Physical activity and nutrition-based  MI intervention | Face-to-face | HCP-Patient | Anthropometric outcomes | Two studies demonstrated increases in adherence or attendance to weight management programs. | An MI approach has demonstrated a positive trend regarding anthropometric measures for females in WMI. Empathic communication, non-judgmental responses, autonomy and self-efficacy support, and face-saving interventions, among other MI-based strategies, appear to have merit for consideration as an intervention framework for behavior changes that can have positive impact on females’ anthropometric outcomes. Higher dosage and interventions taking place over several months appear to be important for impact on anthropometric outcomes. HCPs may consider MI when treating or preventing excess weight gain among females’ due to potential current and future associated health risks. |
| Varela^92^  2021  NR  SR-Network MA  15 Studies | Adult population (18–65 years) with overweight and obesity, with a BMI between 25 and 39.9 kg/m2  (n=2426) | Web-based behavioural programs for overweight and obesity | Web-based (website or website combined with other technological devices) | NS | Primary outcome body weight change (kg). Secondary outcomes: maintenance of weight loss, dropout rates. | There were not relevant differences in dropout between active conditions (IC-W 19.9%; MC-W 14.4%; GSH-W 20.2%; SH-W 14.9% and SH 16.3%). Wait-list was the condition with the lowest rate of dropout 7.8%. We separate web-based programs from self-help and wait-list to analyse the dropout rates. In total, web-based programs obtained 17.3% of dropout compared with 13.1% in the other conditions. | Network meta-analysis has been a helpful tool to analyse the efficacy of web-based programs for people with obesity. Intensive Contact Web-based interventions have obtained the first position in the ranking, proving the relevance of personalized feedback. Further investigation is needed, but these results are a first step in the design of the internet-delivered treatments, an innovative field of research. Moreover, the results of this review support the importance of focusing on the person who suffers obesity instead of the diagnosis or symptoms of this problem. |
| Weerasekara^93^  2016  Workplace  SR-MA  23 Studies | Adults employed at a workplace who were 18 years or older, both sexes, any weight status, and with or without co-existing risk factors (low physical activity and elevated lipid levels) or comorbidities (diabetes and hypertension)  (n=12545) | Workplace interventions to prevent weight gain, facilitate weight loss, or manage weight status. Dietary, physical activity, financial incentives, behaviour change and goal setting, health risk appraisal with feedback, or workplace environmental modifications | Face-to-face | NS | Change in weight, BMI, and/or body fat | Only 3 of the 18 studies with data for 6–12 months had significant mean weight loss, with mean values ranging from −3.95 to −8.80 kg. Those studies were from the USA and Australia, and it may be noteworthy that none of them included a financial incentive for weight loss or attendance at sessions for intervention implementation. | Randomized trials evaluating weight management interventions in worksites have been conducted in the USA, Europe, and several other countries. A small number of interventions have been extremely successful in achieving clinically significant weight loss over a period of 12 months. |
| Wong^94^  2022  Hospital, Community  SR-MA  30 Studies | Adults aged 18-64  years living with obesity (BMI ≥ 30 kg/m2) or overweight (BMI= 23–  29.9 kg/m2)  (n=5391) | Wearable technology lifestyle interventions | Wearable technology (defined as digital gadgets worn by individuals that link to a website that performs functions such as activity tracking, real-time feedback, coaching, goal setting, social support networks, competitions and action planning). | NS | Primary outcomes: weight/ weight change (kg),  steps per day/ change, physical activity intensity change (min/day). Secondary outcomes: BMI/ BMI Change (kg/m2), WC/ WC change (cm), energy intake (kcal/day), fat/ fat change/ mass (%, kg)  , systolic pressure/ change (mmHg),  diastolic pressure/ change (mmHg) | The attrition rates ranged from 0% to 47.8%. Sixteen trials had a high attrition rate (>20%), and 11 of them used the intention-to-treat analysis or missing data management to impute the missing data. Five of the 30 trials (16.66%) were rated at high risk of attrition bias because a high attrition rate was found without using intention-to-treat analysis or missing data management. | Lifestyle interventions delivered by wearable technology can generate change in weight, BMI, WC, physical activity and blood pressure. Given that the majority of objective results were assessed as moderate to high, it is recommended that multicomponent interventions be applied among young overweight and adults living with obesity in non-Asian populations, particularly in the hospital or clinical settings and over long durations. Subgroup and meta-regression analyses are useful for developing effective interventions. |
| Zheng^95^  2014  Various settings  SR-MA  17 Studies | Adults living with overweight or obesity  (n=7269) | Weight loss intervention strategies including self-weighing | Face-to-face, telephone, internet, phone posted cards (some group) | NS | Self-weighing behaviour, weight change, psychological outcomes | Retention rates varied from 13 to 96%, with 10 studies retaining >80% of participants, five retaining 60-80% and two retaining less than 60%. | The findings provide evidence that regular self-weighing has been positively associated with weight loss and not with negative psychological outcomes. |

**Abbreviations:** AHP- Allied Health Professionals, BCT- Behaviour Change Techniques, BMI-Body Mass Index, BRFSS-Behavioural Risk Factor Surveillance Survey, BWLP- Behavioural Weight Loss Programmes, CKD-Chronic Kidney Disease, FG-Fasting Glucose, GDM-Gestational Diabetes Mellitus, GSH-W-Guided Self-Help Web, HCP-Healthcare Professionals, HRQOL-Health-related Quality of Life, IC-W-Intensive Contact Web, ID-Intellectual Disability, IGF- IWLPF- Internet-delivered weight loss interventions providing personalized feedback, LTHC-Long-term Health Condition, MC-W-Minimal Contact Web, MI-Motivational Interviewing, MVPA-Moderate-to-vigorous physical activity, NHANES-National Health and Nutrition Examination Survey, NHIS- National Health Interview Survey, NR-Not Reported, NS-Not Specified, PA-Physical Activity, PCP-Primary Care Provider, PCN-Primary Care Nurse, PHCP- Primary Healthcare Professionals, PHE-Public Health England, PRO-Patient Reported Outcomes, QOL-Quality of life, ROB2-Risk of Bias-2, SH-W-Self-Help Web, SMS-Short Messaging Service, SR-QUAL-Systematic Review Qualitative/ Narrative Synthesis, SR-QUANT-Systematic Review Quantitative Synthesis, SR-MA-Systematic Review with Meta-Analysis, TTM SOC-Transtheoretical Model Stages of Change, US-United States, VLCD-Very Low Calorie Diet, VLED-Very Low Energy Diet, WC-Waist Circumference, WHR -Waist-to-hip ratio, WMI-Weight Management Interventions, WLI-Weight Loss Intervention, WLP-Weight Loss Programme.

Table S10 Moderate, Low and Very Low Confidence Review Findings coded to TDF.

| Cross-Cutting Finding: Mode of Delivery, Relationships and Retention | |
| --- | --- |
| Moderate Confidence | |
| Knowledge,  Skills  Memory, attention and decision processes | Individual characteristics were not well reported across reviews. Only one review^96^ reported specifically on the participant characteristics of gender, age, education, ethnic group and socioeconomic status in relation to engagement within remote interventions. The value of incentives and rewards to promote engagement in BWMI was shown to be related to socioeconomic status. Ethnicity although often inadequately reported was shown to be a determining factor in relationships with providers. Older people were less likely to consent to take part and struggled to engage with remote interventions due to digital literacy, sensory and cognitive limitations. Education was also a factor with levels of literacy and skill a barrier to engagement with remote interventions. The symptoms of SMI, along with societal stigma about these symptoms, can lead people to withdraw from situations like a BWMI^97^. |
| Relationships and Retention | |
| Moderate Confidence (n=3) | |
| Social Influences (n=1) | Several reviews highlighted that engagement is an “under-considered factor in research design”^98^. These reviews had difficulties consolidating evidence regarding retention as commonly there was no clear and consistent definition of engagement among studies. The lack of clarity and variation in terms of conceptualising and operationalising engagement with a study and/ or programme-related content made it difficult to differentiate between measures of retention. This prevented direct comparisons of relational approaches such as Motivational Interviewing on retention and limited the ability to draw conclusions about the influence of relationships on both weight-related outcomes and retention. For example, the low number of included studies and high heterogeneity between studies meant the only meta-analysis investigating relationships and retention the authors were unable to reach conclusions on the effect of primary care relationships on retention ^8,98,99^. |
| Environmental context and Resources (n=1) | Contextual factors, such as a lack of equipment, facilities and resources (e.g. room, furniture, transportation) to provide appropriate care created stigmatising interactions between users and providers, the consequence of which was a negative impact on engagement with healthcare services^11,96,100^. |
| Intention (n=1) | When and how interventions were delivered were important factors facilitating high levels of engagement and retention. One review^11^ described how providers perceived pregnancy to be a good time to intervene as women were motivated and engagement enhanced due to parental desire to protect their unborn child. Another review^99^ provided evidence which demonstrated high retention across studies among BWMIs which incorporated a Motivational Interviewing communication approach.^11,97,99^ |
| Low Confidence | |
| Beliefs about consequences (n=1) | Providers beliefs about the negative consequences of weight-related discussions created a barrier for users. Weight-loss discussions were avoided as many considered addressing obesity such as within maternity services^11^ would negatively impact user-provider relationships and lead to disengagement from services. Users negative perceptions regarding remote interventions including concerns with privacy and technology anxiety were barriers to engagement^11,96^. |
| Very low Confidence | |
| Social/ Professional Role or Identity (n=1) | When support was given from the primary care system, this empowered providers (e.g. by enhancing knowledge and training) to address obesity. Support enabled providers to take an unambiguous position, reducing confusion and uncertainty around the practitioner role, leading to improved user engagement^101^. |
| Barriers and Facilitators Relationships | |
| Moderate Confidence Findings (n=12) | |
| Social/ Professional Role or Identity (n=3) | There was no consistency in views regarding the influence of provider weight status. Users had mixed views about their preferences and how they valued advice in terms of a provider with a higher body weight or someone who was considered “slim”. Providers were also unclear if their own weight status was a barrier or facilitator to effective weight-related communication. Some considered having a lower body weight challenged their authenticity, others with a higher body weight felt hypocritical managing weight loss.^11,102-104^ |
|  | The acceptability of taking a role in weight management was discussed by both users and providers. Users generally wanted providers to take the lead and initiate weight-related interactions. In some instances, they reported that providers did take an active part in weight management. Some providers regarded obesity as an important issue that was within the scope of their professional role. Role acceptance among providers was supported when there was assistance from a multi-disciplinary team where they could call in expertise and when obesity was linked to existing co-morbidities. Existence of a co-morbidity made weight more medicalised and therefore “doctorable”, reportedly enabling weight loss discussions and making interactions less difficult for healthcare providers^11,100-105^. |
| Beliefs about capabilities (n=2) | There were doubts about capabilities from both provider and user perspectives. Providers lacked confidence that users’ had the necessary capabilities to make and sustain the changes required for weight loss. Users doubted that providers were capable of helping them to make the necessary changes. Doubts in this regard were also expressed by providers who lacked confidence and self-efficacy regarding their ability to deliver weight management. This led to a reluctance among providers to raise the issue or discuss weight loss. There was a belief that they would ‘do harm’ by communicating about weight loss or participants will get upset or react badly^11,101,103,105,106^. |
|  | Increased confidence among providers was related to training delivery and content, taking part in research, as well as intervention components. Skills training using the 5As model and training which incorporated role-playing and interactive components demonstrated improvements in physician confidence to deliver weight-related consultations. Providers felt more confident in their ability to provide counselling when interventions were specifically related to physical activity or nutrition advice and these were more routinely addressed components of weight management. When providers had belief in user capabilities this was associated with higher confidence among users to make the necessary changes^11,104,105,107,108^. |
| Optimism (n=2) | Both providers and users were pessimistic about the possibility of successful weight loss. Providers lacked belief in the effectiveness and efficacy of weight loss interventions due to limitations in skills and knowledge, the challenge of obesity, previous experience and the confines of the healthcare system Providers didn’t want to offer treatments they thought were unlikely to work, which might damage credibility which was associated with the sense of powerlessness. Providers also expressed low expectations that users could successfully lose weight which was associated with the perception that providing an intervention was a waste of their time. Users had limited expectations regarding success in relation to weight loss. They believed that they were not treated equally, with their higher weight status compromising treatment options leaving them at a healthcare disadvantage. Some also felt demotivated by the language used in weight management such as being called ‘obese’ leaving users feeling hopeless and powerless as a result^11,100-105^. |
|  | Some providers were optimistic that there were effective weight loss interventions and that their actions and involvement in weight management could benefit users. There was some evidence that users trusted that providers were on their side and were acting in their best interests in relation to weight loss interventions^11,102,105,109^. |
| Reinforcement (n=1) | Both users and providers recognised the importance of good user-providers relationships as fundamental to facilitate positive reactions. Positive reinforcement by acknowledgement of weight loss efforts and achievements strengthened motivation. Financial incentives or reimbursement from insurance programmes were considered useful to motivate providers to train in communication/ counselling approaches such as Motivational Interviewing and encourage prioritisation of weight management^97,102,103,107,109^ |
| Intention (n=1) | Proactive support between sessions was considered important to translate intention into action^97^. Use of a communication style that addresses motivation such as Motivational Interviewing was considered by both providers and users to be an important method to complement behavioural techniques; satisfy psychological needs; enhance weight-related communication and improve user outcomes in different settings. However, effectiveness may be dependent not only on MI training/skill competence but also on the mode of delivery, i.e. telephone versus in-person or remote. Phone coaching using a Motivational Interviewing approach was shown to be as effective as in-person coaching^99^. Delivery by telephone was thought to be able to encompass key mechanisms of MI such as compassion and developing autonomy. This may not translate into e-health delivery of MI where there is no human contact^97,101,106-108,110-112^. |
| Social Influences (n=2) | Both users and providers described the impact of weight bias in terms of suboptimal communication and difficulties within user-provider relationships. Users’ weight status commonly determined what, if any, intervention was offered and how it was approached; weight assessment might only occur if the individual was visibly overweight or health problems dismissed as weight-related. Women living with obesity, for example, tended to receive inaccurate weight gain advice with recommendations exceeding guidelines with less concern shown for those with a higher body weight^106^. There was some suggestion of reciprocal bias with users also judging providers with a higher body weight^104^. Users reported the use of discriminatory language and providers holding stigmatising beliefs, such as the perception that users were lazy, unhealthy, in denial, unwilling, and unmotivated. Negative experiences left users feeling vulnerable and dismissed, distrust of providers, poorer relationships and perpetuated a detrimental cycle of disempowerment and ambivalence. Avoidance of weight-related discussions were perceived by users as stigmatising, “*the doctors' silence reflected a perception that person was not worthy of their time*”^102^. Conversely providers' knowledge and understanding of weight bias and the associated difficulties navigating stigma lead to providers avoiding intervening in weight^11,100-106,108^. |
|  | Hierarchical support (e.g. management) from within the healthcare system was important to empower practitioners and in turn users. This provided an environment to foster trusting, supportive user-provider relationships, an essential feature of successful weight management interventions. Providers recognised that self-awareness of weight bias and taking action to address prejudices could facilitate relationships. They also considered a supportive peer learning environment as well as feedback from users beneficial to minimise negative weight-related interactions. In some cases, users and providers valued human contact, including hybrid methods of delivery, over remote-only support for the development of bonds and facilitation of engagement and motivation^11,96,97,100,101,105,109,113^. |
| Emotions (n=1) | Users had negative feelings toward weight loss programmes, providers and weight-related communication. Negative feelings were associated with the language used around weight such as using the term “obese”. They perceived weight loss programmes to be humiliating and providers reluctance to talk about weight felt stigmatising. In some cases, users were disappointed by providers approach to weight, such as the lack of empathy shown and dismissive attitude^100-102,109^. |
| Low Confidence Findings | |
| Behavioural Regulation (Goals) | Goal-setting was a specific behaviour change technique considered central to successful weight management interventions and strong therapeutic relationships. Directiveness regarding goal-setting and feedback were considered important alongside the establishment of supportive relationships. A high level of provider direction in terms of energy intake, weight and exercise goals and tracking progress were notable within more successful behavioural weight management interventions and underpinning self-regulation^8,96,107,109,112,113^. |
| Memory, attention and decision processes (n=1) | Users and providers both valued the opportunity to be involved in decision-making. Users wanted to be offered a range of treatment options in relation to obesity. This seemed to legitimise their concerns about their weight and built trust with providers reducing the sense of powerlessness and motivating them to make changes. Providers liked to have some flexibility to make choices about interventions such decision-making around tailoring^8,100,101,109^. |
| Very Low Confidence Findings | |
| Beliefs about consequences (n=1) | Providers believed that they could mitigate the negative consequences of interactions and support ongoing positive user-provider relationships by avoiding weight-related discussions by or only intervening when there was a level of engagement and motivation to change such as during pregnancy^11,101^. |
| Reinforcement (n=1) | Among users there was a perception that they were being punished by providers for having a higher body weight by being denied treatments (inequitable treatment)^102^. |
| Intentions (n=1) | Providers considered user motivation key without it users would be unsuccessful in their weight loss efforts. Providers were also less motivated to support women during pregnancy where they believed women were not motivated to change or intervening in weight loss could negatively impact the relationship^11^.Previous negative interactions with providers led to dissatisfaction with these encounters among users and were believed to counteract any positive interaction such as those using a Motivational Interviewing approach. Users with experience of Serious Mental Illness (SMI) had difficulties with weight loss due to fluctuating motivation, symptoms and medication side effects^11,97,101^ |
| Memory, attention and decision processes (n=1) | Decision-making around treatments for people living with obesity was viewed as complex. Weight bias may impact providers' decision-making, accounting for variations in clinical practice. Users were aware of the potential healthcare disadvantages that they face due to their higher body weight. This was related to a sense of powerlessness and lack of control in decision-making regarding their health^100^. |

Table S11 Changes to Protocol

| PROSPERO Overview Protocol | Overview |
| --- | --- |
| Quality assessment - ROBIS | AMSTAR with adaptations to fit with GRADE CerQUAL process. |
| Overlap. Where there is significant overlap (>70% proportion of primary studies in one systematic review found in another) the more recent review with the greatest number of studies will be extracted based on Lunny (2021)^114^. | Overlap - Graphical Representation of Overlap for OVErviews (GROOVE) tool based on Bracchiglione (2022)^115^ – overlap for entire matrix and for pairs of reviews very high overlap >15%. |

**References**

1.	Gates M, Gates A, Pieper D, et al. Reporting guideline for overviews of reviews of healthcare interventions: development of the PRIOR statement. *BMJ*. Aug 9 2022;378:e070849. doi:10.1136/bmj-2022-070849

2.	Dombrowski SU, O'Carroll RE, Williams B. Form of delivery as a key 'active ingredient' in behaviour change interventions. *Br J Health Psychol*. Nov 2016;21(4):733-740. doi:10.1111/bjhp.12203

3.	Michie S, West R, Finnerty AN, et al. Representation of behaviour change interventions and their evaluation: Development of the Upper Level of the Behaviour Change Intervention Ontology. *Wellcome Open Res*. 2020;5:123. doi:10.12688/wellcomeopenres.15902.2

4.	Bordin ES. The Generalisability of the Psychoanalytic Concept of the Working Alliance. *PSYCHOTHERAPY: THEORY, RESEARCH AND PRACTICE*. 1979;16(3)

5.	Bordin ES. A Working Alliance Based Model of Supervision. *The Counseling Psychologist*. 1983;11(1)

6.	Gleitman H. *Basic Pscyhology 4th Edition*. W W Norton and Company; 1996.

7.	Mercer SW, Maxwell M, Heaney D, Watt GC. The consultation and relational empathy (CARE) measure: development and preliminary validation and reliability of an empathy-based consultation process measure. *Fam Pract*. Dec 2004;21(6):699-705. doi:10.1093/fampra/cmh621

8.	Sturgiss EA, O'Brien K, Elmitt N, et al. Obesity management in primary care: systematic review exploring the influence of therapeutic alliance. *Fam Pract*. Sep 25 2021;38(5):644-653. doi:10.1093/fampra/cmab026

9.	Michie S, Ashford S, Sniehotta FF, Dombrowski SU, Bishop A, French DP. A refined taxonomy of behaviour change techniques to help people change their physical activity and healthy eating behaviours: the CALO-RE taxonomy. *Psychol Health*. Nov 2011;26(11):1479-98. doi:10.1080/08870446.2010.540664

10.	Shea BJ, Reeves BC, Wells G, et al. AMSTAR 2: a critical appraisal tool for systematic reviews that include randomised or non-randomised studies of healthcare interventions, or both. *BMJ*. Sep 21 2017;358:j4008. doi:10.1136/bmj.j4008

11.	Heslehurst N, Newham J, Maniatopoulos G, Fleetwood C, Robalino S, Rankin J. Implementation of pregnancy weight management and obesity guidelines: a meta-synthesis of healthcare professionals' barriers and facilitators using the Theoretical Domains Framework. *Obes Rev*. Jun 2014;15(6):462-86. doi:10.1111/obr.12160

12.	Alnaabi Y, Ibrahim N, Singh Dhillon J. A systematic literature review of persuasive design features utlized by mobile-based obesity interventions. . *Journal of Theoretical and Applied Information Technology*. 2021;99(2)

13.	Alnuaimi A, Rawaf S, Hassounah S, Chehab M. Use of mobile applications in the management of overweight and obesity in primary and secondary care. *JRSM Open*. Mar 2019;10(3):2054270419843826. doi:10.1177/2054270419843826

14.	Barnes RD, Ivezaj V. A systematic review of motivational interviewing for weight loss among adults in primary care. *Obes Rev*. Apr 2015;16(4):304-18. doi:10.1111/obr.12264

15.	Bennett GG, Steinberg DM, Stoute C, et al. Electronic health (eHealth) interventions for weight management among racial/ethnic minority adults: a systematic review. *Obes Rev*. Oct 2014;15 Suppl 4:146-58. doi:10.1111/obr.12218

16.	Burnet DL, Elliott LD, Quinn MT, Plaut AJ, Schwartz MA, Chin MH. Preventing diabetes in the clinical setting. *J Gen Intern Med*. Jan 2006;21(1):84-93. doi:10.1111/j.1525-1497.2005.0277.x

17.	Carraca E, Encantado J, Battista F, et al. Effective behavior change techniques to promote physical activity in adults with overweight or obesity: A systematic review and meta-analysis. *Obes Rev*. Jul 2021;22 Suppl 4(Suppl 4):e13258. doi:10.1111/obr.13258

18.	Cheatham SW, Stull KR, Fantigrassi M, Motel I. The efficacy of wearable activity tracking technology as part of a weight loss program: a systematic review. *J Sports Med Phys Fitness*. Apr 2018;58(4):534-548. doi:10.23736/S0022-4707.17.07437-0

19.	Chisholm A, Hart J, Mann KV, Harkness E, Peters S. Preparing medical students to facilitate lifestyle changes with obese patients: a systematic review of the literature. *Acad Med*. Jul 2012;87(7):912-23. doi:10.1097/ACM.0b013e3182580648

20.	Chopra S, Malhotra A, Ranjan P, et al. Predictors of successful weight loss outcomes amongst individuals with obesity undergoing lifestyle interventions: A systematic review. *Obes Rev*. Mar 2021;22(3):e13148. doi:10.1111/obr.13148

21.	Cooper L, Ryan CG, Ells LJ, et al. Weight loss interventions for adults with overweight/obesity and chronic musculoskeletal pain: a mixed methods systematic review. *Obes Rev*. Jul 2018;19(7):989-1007. doi:10.1111/obr.12686

22.	Coupe N, Peters S, Rhodes S, Cotterill S. The effect of commitment-making on weight loss and behaviour change in adults with obesity/overweight; a systematic review. *BMC Public Health*. Jun 24 2019;19(1):816. doi:10.1186/s12889-019-7185-3

23.	Flodgren G, Gonçalves-Bradley DC, Summerbell CD. Interventions to change the behaviour of health professionals and the organisation of care to promote weight reduction in children and adults with overweight or obesity. *Cochrane Database Syst Rev*. Nov 30 2017;11(11):Cd000984. doi:10.1002/14651858.CD000984.pub3

24.	Gostoli S, Raimondi G, Popa AP, Giovannini M, Benasi G, Rafanelli C. Behavioral Lifestyle Interventions for Weight Loss in Overweight or Obese Patients with Type 2 Diabetes: A Systematic Review of the Literature. *Curr Obes Rep*. Jun 2024;13(2):224-241. doi:10.1007/s13679-024-00552-5

25.	Gudzune KA, Doshi RS, Mehta AK, et al. Efficacy of commercial weight-loss programs: an updated systematic review. *Ann Intern Med*. Apr 7 2015;162(7):501-12. doi:10.7326/M14-2238

26.	Harper C, Maher J, Grunseit A, Seimon RV, Sainsbury A. Experiences of using very low energy diets for weight loss by people with overweight or obesity: a review of qualitative research. *Obes Rev*. Oct 2018;19(10):1412-1423. doi:10.1111/obr.12715

27.	Hartmann-Boyce Jamie JSA, Fletcher Ben R., and Aveyard Paul. Self-Help for Weight Loss in Overweight and Obese Adults: Systematic Review and Meta-Analysis. *American Journal of Public Health*. 2015;105(e43–e57)doi:10. 2105/AJPH.2014.302389

28.	Johnson M, Campbell F, Messina J, Preston L, Buckley Woods H, Goyder E. Weight management during pregnancy: a systematic review of qualitative evidence. *Midwifery*. Dec 2013;29(12):1287-96. doi:10.1016/j.midw.2012.11.016

29.	Khaylis A, Yiaslas T, Bergstrom J, Gore-Felton C. A review of efficacious technology-based weight-loss interventions: five key components. *Telemed J E Health*. Nov 2010;16(9):931-8. doi:10.1089/tmj.2010.0065

30.	Makin H, Chisholm A, Fallon V, Goodwin L. Use of motivational interviewing in behavioural interventions among adults with obesity: A systematic review and meta-analysis. *Clin Obes*. Aug 2021;11(4):e12457. doi:10.1111/cob.12457

31.	Michalopoulou M, Ferrey AE, Harmer G, et al. Effectiveness of Motivational Interviewing in Managing Overweight and Obesity : A Systematic Review and Meta-analysis. *Ann Intern Med*. Jun 2022;175(6):838-850. doi:10.7326/M21-3128

32.	Manzoni GM, Pagnini F, Corti S, Molinari Ea, Castelnuovo G. Internet-Based Behavioral Interventions for Obesity: An Updated Systematic Review. *Clinical Practice & Epidemiology in Mental Health*. 2011;17:19-28.

33.	Marques ES, Interlenghi GS, Leite TH, et al. Primary care–based interventions for treatment of obesity: a systematic review. *Public Health*. 2021/06/01/ 2021;195:61-69. doi:<https://doi.org/10.1016/j.puhe.2021.04.012>

34.	McDonald MD, Hunt K, Sivaramakrishnan H, et al. A systematic review examining socioeconomic factors in trials of interventions for men that report weight as an outcome. *Obes Rev*. Jul 2022;23(7):e13436. doi:10.1111/obr.13436

35.	Morrow A, Walker K, Calder-MacPhee N, Ozakinci G. The active ingredients of physical activity and / or dietary workplace-based interventions to achieve weight loss in overweight and obese healthcare staff: a systematic review. *J Behav Med*. Jun 2022;45(3):331-349. doi:10.1007/s10865-021-00279-x

36.	Pearson ES. Goal setting as a health behavior change strategy in overweight and obese adults: a systematic literature review examining intervention components. *Patient Educ Couns*. Apr 2012;87(1):32-42. doi:10.1016/j.pec.2011.07.018

37.	Perperidi M, Saliari D, Christakis C, et al. Identifying the effective behaviour change techniques in nutrition and physical activity interventions for the treatment of overweight/obesity in post-treatment breast cancer survivors: a systematic review. *Cancer Causes Control*. Aug 2023;34(8):683-703. doi:10.1007/s10552-023-01707-w

38.	Pritchard EK, Kim HC, Nguyen N, van Vreden C, Xia T, Iles R. The effect of weight loss interventions in truck drivers: Systematic review. *PLoS One*. 2022;17(2):e0262893. doi:10.1371/journal.pone.0262893

39.	Robertson C, Archibald D, Avenell A, et al. Systematic reviews of and integrated report on the quantitative, qualitative and economic evidence base for the management of obesity in men. *Health Technol Assess*. May 2014;18(35):v-vi, xxiii-xxix, 1-424. doi:10.3310/hta18350

40.	Rose SA, Poynter PS, Anderson JW, Noar SM, Conigliaro J. Physician weight loss advice and patient weight loss behavior change: a literature review and meta-analysis of survey data. *Int J Obes (Lond)*. Jan 2013;37(1):118-28. doi:10.1038/ijo.2012.24

41.	Sherrington A, Newham JJ, Bell R, Adamson A, McColl E, Araujo-Soares V. Systematic review and meta-analysis of internet-delivered interventions providing personalized feedback for weight loss in overweight and obese adults. *Obes Rev*. Jun 2016;17(6):541-51. doi:10.1111/obr.12396

42.	Sherson EA, Yakes Jimenez E, Katalanos N. A review of the use of the 5 A's model for weight loss counselling: differences between physician practice and patient demand. *Fam Pract*. Aug 2014;31(4):389-98. doi:10.1093/fampra/cmu020

43.	Tang JC, Abraham C, Greaves CJ, Nikolaou V. Self-directed interventions to promote weight loss: a systematic review and meta-analysis. *Health Psychol Rev*. Sep 2016;10(3):358-72. doi:10.1080/17437199.2016.1172979

44.	Tapsell LC, Neale EP. The Effect of Interdisciplinary Interventions on Risk Factors for Lifestyle Disease: A Literature Review. *Health Educ Behav*. Jun 2016;43(3):271-85. doi:10.1177/1090198115601092

45.	Walsh MAF, Fahy KM. Interaction between primary health care professionals and people who are overweight or obese: A critical review. *Aust J Adv Nurs [Internet]*. 2012;doi:<https://doi.org/10.37464/2012.292.1637>

46.	Wieland LS, Falzon L, Sciamanna CN, et al. Interactive computer-based interventions for weight loss or weight maintenance in overweight or obese people. *Cochrane Database Syst Rev*. Aug 15 2012;8(8):CD007675. doi:10.1002/14651858.CD007675.pub2

47.	Young MD, Morgan PJ, Plotnikoff RC, Callister R, Collins CE. Effectiveness of male-only weight loss and weight loss maintenance interventions: a systematic review with meta-analysis. *Obes Rev*. May 2012;13(5):393-408. doi:10.1111/j.1467-789X.2011.00967.x

48.	Abbott S, Smith E, Tighe B, Lycett D. Group versus one-to-one multi-component lifestyle interventions for weight management: a systematic review and meta-analysis of randomised controlled trials. *J Hum Nutr Diet*. Jun 2021;34(3):485-493. doi:10.1111/jhn.12853

49.	Aguilar-Martinez A, Sole-Sedeno JM, Mancebo-Moreno G, Medina FX, Carreras-Collado R, Saigi-Rubio F. Use of mobile phones as a tool for weight loss: a systematic review. *J Telemed Telecare*. Sep 2014;20(6):339-49. doi:10.1177/1357633X14537777

50.	Alamnia TT, Tesfaye W, Kelly M. The effectiveness of text message delivered interventions for weight loss in developing countries: A systematic review and meta-analysis. *Obes Rev*. Jan 2022;23(1):e13339. doi:10.1111/obr.13339

51.	Balk EM, Earley A, Raman G, Avendano EA, Pittas AG, Remington PL. Combined Diet and Physical Activity Promotion Programs to Prevent Type 2 Diabetes Among Persons at Increased Risk: A Systematic Review for the Community Preventive Services Task Force. *Ann Intern Med*. Sep 15 2015;163(6):437-51. doi:10.7326/M15-0452

52.	Bassi N, Karagodin I, Wang S, et al. Lifestyle modification for metabolic syndrome: a systematic review. *Am J Med*. Dec 2014;127(12):1242 e1-10. doi:10.1016/j.amjmed.2014.06.035

53.	Beleigoli AM, Andrade AQ, Cancado AG, Paulo MN, Diniz MFH, Ribeiro AL. Web-Based Digital Health Interventions for Weight Loss and Lifestyle Habit Changes in Overweight and Obese Adults: Systematic Review and Meta-Analysis. *J Med Internet Res*. Jan 8 2019;21(1):e298. doi:10.2196/jmir.9609

54.	Blue CL, Black DR. Synthesis of intervention research to modifcy physical activity and dietary behaviours. *Research and Theory for Nursing Practice*. 2005;19(1)

55.	Booth HP, Prevost TA, Wright AJ, Gulliford MC. Effectiveness of behavioural weight loss interventions delivered in a primary care setting: a systematic review and meta-analysis. *Fam Pract*. Dec 2014;31(6):643-53. doi:10.1093/fampra/cmu064

56.	Brown I, Psarou A. Literature review of nursing practice in managing obesity in primary care: developments in the UK. *J Clin Nurs*. Jan 2008;17(1):17-28. doi:10.1111/j.1365-2702.2006.01862.x

57.	Brown I, Gould J. Decisions about weight management: a synthesis of qualitative studies of obesity. *Clin Obes*. Apr 2011;1(2-3):99-109. doi:10.1111/j.1758-8111.2011.00020.x

58.	Burgess E, Hassmen P, Pumpa KL. Determinants of adherence to lifestyle intervention in adults with obesity: a systematic review. *Clin Obes*. Jun 2017;7(3):123-135. doi:10.1111/cob.12183

59.	Byaruhanga J, Atorkey P, McLaughlin M, et al. Effectiveness of Individual Real-Time Video Counseling on Smoking, Nutrition, Alcohol, Physical Activity, and Obesity Health Risks: Systematic Review. *J Med Internet Res*. Sep 11 2020;22(9):e18621. doi:10.2196/18621

60.	Comsa L, David O, David D. Outcomes and mechanisms of change in cognitive-behavioral interventions for weight loss: A meta-analysis of randomized clinical trials. *Behav Res Ther*. Sep 2020;132:103654. doi:10.1016/j.brat.2020.103654

61.	Conley MM, McFarlane CM, Johnson DW, Kelly JT, Campbell KL, MacLaughlin HL. Interventions for weight loss in people with chronic kidney disease who are overweight or obese. *Cochrane Database Syst Rev*. Mar 30 2021;3(3):CD013119. doi:10.1002/14651858.CD013119.pub2

62.	Cradock KA, G OL, Finucane FM, Gainforth HL, Quinlan LR, Ginis KA. Behaviour change techniques targeting both diet and physical activity in type 2 diabetes: A systematic review and meta-analysis. *Int J Behav Nutr Phys Act*. Feb 8 2017;14(1):18. doi:10.1186/s12966-016-0436-0

63.	Crawford R, Glover L. The impact of pre-treatment weight-loss expectations on weight loss, weight regain, and attrition in people who are overweight and obese: a systematic review of the literature. *Br J Health Psychol*. Sep 2012;17(3):609-30. doi:10.1111/j.2044-8287.2011.02059.x

64.	Dombrowski SU, Sniehotta FF, Avenell A, Johnston M, MacLennan G, Araújo-Soares V. Identifying active ingredients in complex behavioural interventions for obese adults with obesity-related co-morbidities or additional risk factors for co-morbidities: a systematic review. *Health Psychology Review*. 2012;6(1):7-32. doi:10.1080/17437199.2010.513298

65.	Dombrowski SU, Knittle K, Avenell A, Araujo-Soares V, Sniehotta FF. Long term maintenance of weight loss with non-surgical interventions in obese adults: systematic review and meta-analyses of randomised controlled trials. *BMJ*. May 14 2014;348:g2646. doi:10.1136/bmj.g2646

66.	Dounavi K, Tsoumani O. Mobile Health Applications in Weight Management: A Systematic Literature Review. *Am J Prev Med*. Jun 2019;56(6):894-903. doi:10.1016/j.amepre.2018.12.005

67.	Everitt JD, Battista-Dowds EM, Heggs D, Squire ALM. Attrition and Retention in Multidisciplinary Weight Management Interventions for Adults with Obesity: A Systematic Review. 2021;doi:10.21203/rs.3.rs-1128262/v1

68.	Ge L, Sadeghirad B, Ball GDC, et al. Comparison of dietary macronutrient patterns of 14 popular named dietary programmes for weight and cardiovascular risk factor reduction in adults: systematic review and network meta-analysis of randomised trials. *BMJ*. Apr 1 2020;369:m696. doi:10.1136/bmj.m696

69.	Gilinsky AS, Kirk AF, Hughes AR, Lindsay RS. Lifestyle interventions for type 2 diabetes prevention in women with prior gestational diabetes: A systematic review and meta-analysis of behavioural, anthropometric and metabolic outcomes. *Prev Med Rep*. 2015;2:448-61. doi:10.1016/j.pmedr.2015.05.009

70.	Hartmann-Boyce J, Theodoulou A, Oke JL, et al. Association between characteristics of behavioural weight loss programmes and weight change after programme end: systematic review and meta-analysis. *BMJ*. Aug 17 2021;374:n1840. doi:10.1136/bmj.n1840

71.	Hutchesson MJ, Rollo ME, Krukowski R, et al. eHealth interventions for the prevention and treatment of overweight and obesity in adults: a systematic review with meta-analysis. *Obes Rev*. May 2015;16(5):376-92. doi:10.1111/obr.12268

72.	Lemstra M, Bird Y, Nwankwo C, Rogers M, Moraros J. Weight loss intervention adherence and factors promoting adherence: a meta-analysis. *Patient Prefer Adherence*. 2016;10:1547-59. doi:10.2147/PPA.S103649

73.	Leung AWY, Chan RSM, Sea MMM, Woo J. An Overview of Factors Associated with Adherence to Lifestyle Modification Programs for Weight Management in Adults. *Int J Environ Res Public Health*. Aug 16 2017;14(8)doi:10.3390/ijerph14080922

74.	Malik M, Kanwal N, Hussain A. Role of Community Pharmacist and Nutritionists in Obesity Management: A Literature Review. *Asian Journal of Pharmaceutical and Clinical Research*. 2017;10(2)doi:10.22159/ajpcr.2017.v10i2.15603

75.	Mastellos N, Gunn LH, Felix LM, Car J, Majeed A. Transtheoretical model stages of change for dietary and physical exercise modification in weight loss management for overweight and obese adults. *Cochrane Database Syst Rev*. Feb 5 2014;2014(2):Cd008066. doi:10.1002/14651858.CD008066.pub3

76.	Metzendorf MI, Wieland LS, Richter B. Mobile health (m-health) smartphone interventions for adolescents and adults with overweight or obesity. *Cochrane Database Syst Rev*. Feb 20 2024;2(2):Cd013591. doi:10.1002/14651858.CD013591.pub2

77.	Moroshko I, Brennan L, O'Brien P. Predictors of dropout in weight loss interventions: a systematic review of the literature. *Obes Rev*. Nov 2011;12(11):912-34. doi:10.1111/j.1467-789X.2011.00915.x

78.	Naude CE, Brand A, Schoonees A, Nguyen KA, Chaplin M, Volmink J. Low-carbohydrate versus balanced-carbohydrate diets for reducing weight and cardiovascular risk. *Cochrane Database Syst Rev*. Jan 28 2022;1(1):CD013334. doi:10.1002/14651858.CD013334.pub2

79.	Park SH, Hwang J, Choi YK. Effect of Mobile Health on Obese Adults: A Systematic Review and Meta-Analysis. *Healthc Inform Res*. Jan 2019;25(1):12-26. doi:10.4258/hir.2019.25.1.12

80.	Plotnikoff R, Collins CE, Williams R, Germov J, Callister R. Effectiveness of interventions targeting health behaviors in university and college staff: a systematic review. *Am J Health Promot*. May-Jun 2015;29(5):e169-87. doi:10.4278/ajhp.130619-LIT-313

81.	Power D, Jones A, Keyworth C, et al. Emotional Eating Interventions for Adults Living With Overweight and Obesity: A Systematic Review and Meta-Analysis of Behaviour Change Techniques. *J Hum Nutr Diet*. Feb 2025;38(1):e13410. doi:10.1111/jhn.13410

82.	Puigdomenech Puig E, Robles N, Saigí-Rubió F, et al. Assessment of the Efficacy, Safety, and Effectiveness of Weight Control and Obesity Management Mobile Health Interventions: Systematic Review. *JMIR Mhealth Uhealth*. Oct 25 2019;7(10):e12612. doi:10.2196/12612

83.	Reed VA, Schifferdecker KE, Rezaee ME, O'Connor S, Larson RJ. The effect of computers for weight loss: a systematic review and meta-analysis of randomized trials. *J Gen Intern Med*. Jan 2012;27(1):99-108. doi:10.1007/s11606-011-1803-9

84.	Renders CM, Valk GD, Griffin S, Wagner EH, Eijk JT, Assendelft WJ. Interventions to improve the management of diabetes mellitus in primary care, outpatient and community settings. *Cochrane Database Syst Rev*. 2001;2000(1):CD001481. doi:10.1002/14651858.CD001481

85.	Shaikh H, Bradhurst P, Ma LX, Tan SYC, Egger SJ, Vardy JL. Body weight management in overweight and obese breast cancer survivors. *Cochrane Database Syst Rev*. Dec 11 2020;12(12):CD012110. doi:10.1002/14651858.CD012110.pub2

86.	Shannon HH JR, Puro N, Darrell E. Use of Technology in the Management of Obesity: A Literature Review. *Perspect Health Inf Manag*. 2009;1(16)

87.	Shaw R, Bosworth H. Short message service (SMS) text messaging as an intervention medium for weight loss: A literature review. *Health Informatics J*. Dec 2012;18(4):235-50. doi:10.1177/1460458212442422

88.	Smith N, Liu S. A systematic review of the dose-response relationship between usage and outcomes of online physical activity weight-loss interventions. *Internet Interv*. Dec 2020;22:100344. doi:10.1016/j.invent.2020.100344

89.	Spanos D, Melville, C.A. & Hankey, C.R. Weight management interventions in adults with intellectual disabilities and obesity: a systematic review of the evidence. *Nutr J*. 2013;12(132)

90.	Streb AR, Tozetto WR, da Silva CS, Bertuol C, Minatto G, Del Duca GF. Characteristics of interventions with exercise according to the adherence of adults with obesity: A systematic review. *Clin Obes*. Jun 2025;15(3):e12733. doi:10.1111/cob.12733

91.	Suire KB, Kavookjian J, Feiss R, Wadsworth DD. Motivational Interviewing for Weight Management Among Women: a Meta-Analysis and Systematic Review of RCTs. *Int J Behav Med*. Aug 2021;28(4):403-416. doi:10.1007/s12529-020-09934-0

92.	Varela C, Oda-Montecinos C, Andres A, Saldana C. Effectiveness of web-based feedback interventions for people with overweight and obesity: systematic review and network meta-analysis of randomized controlled trials. *J Eat Disord*. Jun 26 2021;9(1):75. doi:10.1186/s40337-021-00432-6

93.	Weerasekara YK, Roberts SB, Kahn MA, LaVertu AE, Hoffman B, Das SK. Effectiveness of Workplace Weight Management Interventions: a Systematic Review. *Curr Obes Rep*. Jun 2016;5(2):298-306. doi:10.1007/s13679-016-0205-z

94.	Wong SH, Tan ZYA, Cheng LJ, Lau ST. Wearable technology-delivered lifestyle intervention amongst adults with overweight and obese: A systematic review and meta-regression. *Int J Nurs Stud*. Mar 2022;127:104163. doi:10.1016/j.ijnurstu.2021.104163

95.	Zheng Y, Klem ML, Sereika SM, Danford CA, Ewing LJ, Burke LE. Self-weighing in weight management: a systematic literature review. *Obesity (Silver Spring)*. Feb 2015;23(2):256-65. doi:10.1002/oby.20946

96.	Whitehall JM, Cook EJ, Vseteckova J, et al. A systematic review of influences on engagement with remote health interventions targeting weight management for individuals living with excess weight. *Int J Obes (Lond)*. Aug 2025;49(8):1427-1468. doi:10.1038/s41366-025-01811-8

97.	Lee C, Piernas C, Stewart C, et al. Identifying effective characteristics of behavioral weight management interventions for people with serious mental illness: A systematic review with a qualitative comparative analysis. *Obes Rev*. Jan 2022;23(1):e13355. doi:10.1111/obr.13355

98.	Ryan K, Dockray S, Linehan C. A systematic review of tailored eHealth interventions for weight loss. *Digit Health*. Jan-Dec 2019;5:2055207619826685. doi:10.1177/2055207619826685

99.	Patel ML, Wakayama LN, Bass MB, Breland JY. Motivational interviewing in eHealth and telehealth interventions for weight loss: A systematic review. *Prev Med*. Sep 2019;126:105738. doi:10.1016/j.ypmed.2019.05.026

100.	Mold F, Forbes A. Patients' and professionals' experiences and perspectives of obesity in health-care settings: a synthesis of current research. *Health Expect*. Jun 2013;16(2):119-42. doi:10.1111/j.1369-7625.2011.00699.x

101.	Henderson E. Obesity in primary care: a qualitative synthesis of patient and practitioner perspectives on roles and responsibilities. *Br J Gen Pract*. Apr 2015;65(633):e240-7. doi:10.3399/bjgp15X684397

102.	Ananthakumar T, Jones NR, Hinton L, Aveyard P. Clinical encounters about obesity: Systematic review of patients' perspectives. *Clin Obes*. Feb 2020;10(1):e12347. doi:10.1111/cob.12347

103.	Warr W, Aveyard P, Albury C, et al. A systematic review and thematic synthesis of qualitative studies exploring GPs' and nurses' perspectives on discussing weight with patients with overweight and obesity in primary care. *Obes Rev*. Apr 2021;22(4):e13151. doi:10.1111/obr.13151

104.	Jeffers L, Manner J, Jepson R, McAteer J. Healthcare professionals' perceptions and experiences of obesity and overweight and its management in primary care settings: a qualitative systematic review. *Prim Health Care Res Dev*. Jan 17 2024;25:e5. doi:10.1017/S1463423623000683

105.	Teixeira FV, Pais-Ribeiro JL, da Costa Maia ÂRP. Beliefs and practices of healthcare providers regarding obesity: a systematic review. *Revista da Associação Médica Brasileira (English Edition)*. 2012;58(2):254-262. doi:10.1016/s2255-4823(12)70189-3

106.	Dieterich R, Demirci J. Communication practices of healthcare professionals when caring for overweight/obese pregnant women: A scoping review. *Patient Educ Couns*. Oct 2020;103(10):1902-1912. doi:10.1016/j.pec.2020.05.011

107.	Reading JM, Snell LM, LaRose JG. A systematic review of weight-related communication trainings for physicians. *Transl Behav Med*. Oct 12 2020;10(5):1110-1119. doi:10.1093/tbm/ibaa014

108.	McHale CT, Laidlaw AH, Cecil JE. Direct observation of weight-related communication in primary care: a systematic review. *Fam Pract*. Aug 2016;33(4):327-45. doi:10.1093/fampra/cmw045

109.	Avenell A, Robertson C, Skea Z, et al. Bariatric surgery, lifestyle interventions and orlistat for severe obesity: the REBALANCE mixed-methods systematic review and economic evaluation. *Health Technol Assess*. Nov 2018;22(68):1-246. doi:10.3310/hta22680

110.	Version1.2. NHS Long Term Plan (2019).

111.	Samdal GB, Eide GE, Barth T, Williams G, Meland E. Effective behaviour change techniques for physical activity and healthy eating in overweight and obese adults; systematic review and meta-regression analyses. *Int J Behav Nutr Phys Act*. Mar 28 2017;14(1):42. doi:10.1186/s12966-017-0494-y

112.	Munoz Obino KF, Aguiar Pereira C, Caron-Lienert RS. Coaching and barriers to weight loss: an integrative review. *Diabetes Metab Syndr Obes*. 2017;10:1-11. doi:10.2147/DMSO.S113874

113.	Sutcliffe K, Richardson M, Rees R, et al. *What are the critical features of successful Tier 2 weight management programmes? A systematic review to identify the programme characteristics, and combinations of characteristics, that are associated with successful weight loss*. 2016.

114.	Lunny C, Pieper D, Thabet P, Kanji S. Managing Overlap of Primary Studies Results Across Systematic Reviews: Practical Considerations for Authors of Overviews of Reviews. 2021;doi:10.21203/rs.3.rs-145573/v1

115.	Pérez-Bracchiglione J, Meza N, Bangdiwala SI, et al. Graphical Representation of Overlap for OVErviews: GROOVE tool. *Res Synth Methods*. May 2022;13(3):381-388. doi:10.1002/jrsm.1557
